# Draft genome of the milu (*Elaphurus davidianus*)

**DOI:** 10.1093/gigascience/gix130

**Published:** 2017-12-18

**Authors:** Chenzhou Zhang, Lei Chen, Yang Zhou, Kun Wang, Leona G Chemnick, Oliver A Ryder, Wen Wang, Guojie Zhang, Qiang Qiu

**Affiliations:** 1 Center for Ecological and Environmental Sciences, Key Laboratory for Space Bioscience and Biotechnology, Northwestern Polytechnical University, Xi’an, 710072, China; 2 China National Genebank, BGI-Shenzhen, Shenzhen 518083, China; 3 BGI-Shenzhen, Shenzhen 518083, China; 4 San Diego Zoo Institute for Conservation Research, Escondido, CA 92027, USA; 5 Centre for Social Evolution, Department of Biology, Universitetsparken 15, University of Copenhagen, Copenhagen 2100, Denmark

**Keywords:** *Elaphurus davidianus*, Reference genome, Evolution

## Abstract

**Background:**

Milu, also known as Père David's deer (*Elaphurus davidianus*), was widely distributed in East Asia but recently experienced a severe bottleneck. Only 18 survived by the end of the 19th century, and the current population of 4500 individuals was propagated from just 11 kept by the 11th British Duke of Bedford. This species is known for its distinguishable appearance, the driving force behind which is still a mystery. To aid efforts to explore these phenomena, we constructed a draft genome of the species.

**Findings:**

In total, we generated 321.86 gigabases (Gb) of raw DNA sequence from whole-genome sequencing of a male milu deer using an Illumina HiSeq 2000 platform. Assembly yielded a final genome with a scaffold N50 size of 3.03 megabases (Mb) and a total length of 2.52 Gb. Moreover, we identified 20 125 protein-coding genes and 988.1 Mb of repetitive sequences. In addition, homology-based searches detected 280 rRNA, 1335 miRNA, 1441 snRNA, and 893 tRNA sequences in the milu genome. The divergence time between *E. davidianus* and *Bos taurus* was estimated to be about 28.20 million years ago (Mya). We identified 167 species-specific genes and 293 expanded gene families in the milu lineage.

**Conclusions:**

We report the first reference genome of milu, which will provide a valuable resource for studying the species’ demographic history of severe bottleneck and the genetic mechanism(s) of special phenotypic evolution.

## Data Description

### Background

**Figure 1:**
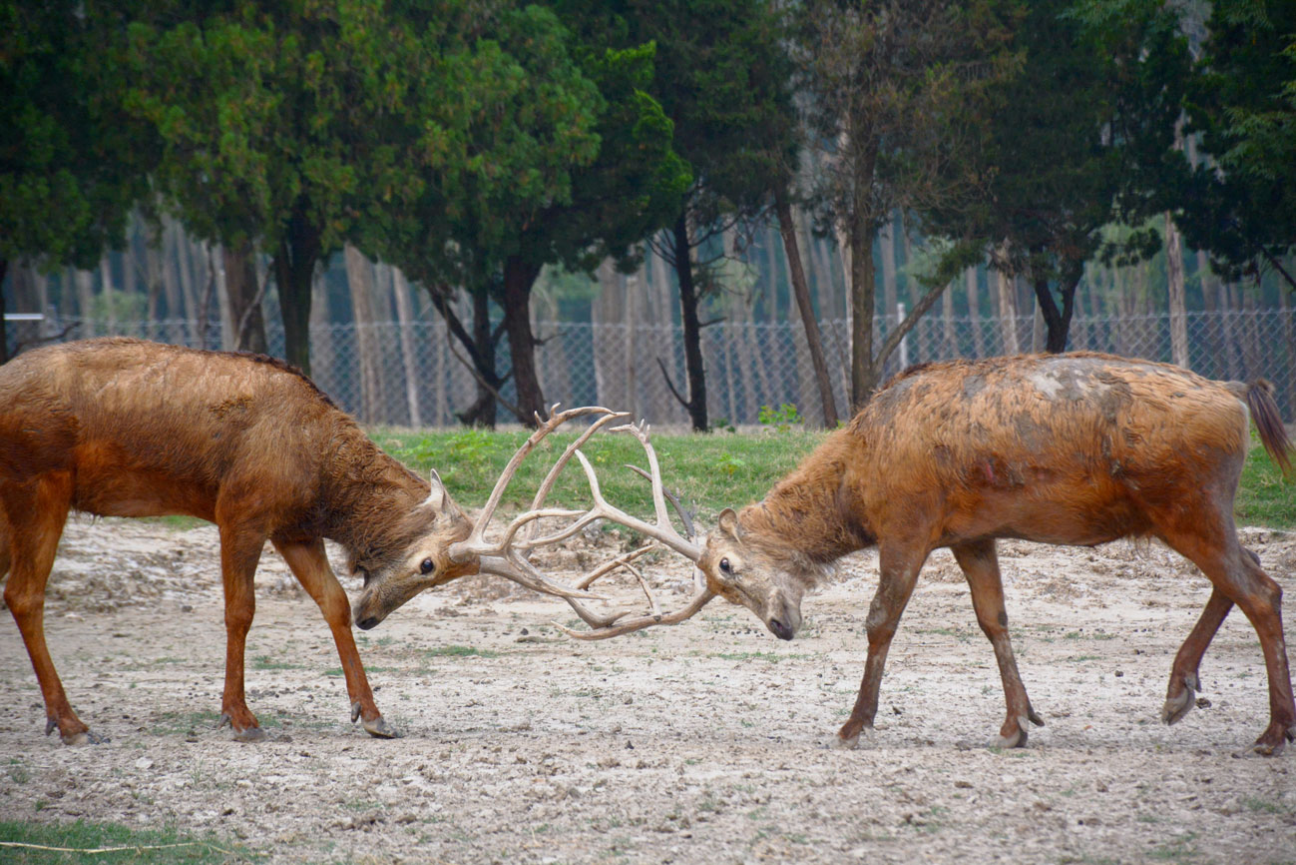
Photo of 2 fighting Père David's deer in Dafeng Milu National Reserves, Jiangsu, China. A red wound was spotted on the body of the deer to the right, and winning such fights generally increases mating chances.

Père David's deer (*Elaphurus davidianus*), named after its western finder (Father Armand David) and called “milu” in China, was an endemic species that was once widely distributed in East Asia [1, 2]. Milu also has a colloquial name in China, *Sibuxiang*, which could be translated as “the 4 unlikes,” because it has the hooves of a cow, head of a horse, antlers of a deer, and tail of a donkey, but is not any of these animals (Fig. 1). Due to intense human and natural pressures, such as excessive hunting by humans and habitat degradation, milu became extinct in China by the end of the 19th century, and only 18 individuals survived in several European zoos at that time. The 18 surviving individuals were collected by the 11th British Duke of Bedford and kept at Woburn Abbey (UK), and only 11 participated in subsequent reproduction [3]. After this severe bottleneck, the milu population started to recover. In the 1980s, dozens were reintroduced into China, and there were more than 1500 in China and more than 3000 globally by 2004 [4]. Milu is highly interesting partly because of this bottleneck and partly because it has atypical features for a cervid, such as a relatively long tail and unique branched antlers. Due to these traits, scientists once identified it as the root of the subfamily Cervinae, but subsequent molecular analysis indicated that milu is closer to the genus *Cervus* [5–9]. However, little is known about the genetic architecture underlying milu's unique phenotypic features and the population dynamics during its recovery from the severe bottleneck. Thus, we constructed a draft genome for the species to facilitate investigation of effects of the severe recent bottleneck and the molecular mechanisms involved in its phenotypic evolution.

### Library construction and filtering

Genomic DNA was extracted from a male milu bred at the San Diego Zoo Safari Park, Escondido, California, USA, utilizing heart tissue collected at necropsy (NCBI Taxonomy ID, 43 332). The extracted DNA was used to construct short-insert libraries (170, 500, and 800 base pairs [bp]), and subsequently long-insert libraries (2, 5, 10, and 20 kilo bases [kb]). A HiSeq 2000 platform (Illumina, San Diego, CA, USA) was subsequently used to sequence paired-end reads of each library based on a whole-genome shotgun sequencing strategy, generating 100-bp and 49-bp reads from the short-insert and long-insert libraries, respectively. In total, a 321.86-Gb raw dataset was obtained (Table S1).

Raw reads were filtered out that had: (1) >5% uncalled (“N”) bases or polyA structure; (2) ≥60 bases with quality scores ≤7 for reads generated from the short-insert library sequences; (3) ≥30 such bases for reads generated from the long-insert library sequences; (4) more than 10 bp aligned to the adapter sequence; (5) read1 and read2 (of a short-insert PE read) overlapping by ≥10 bp, allowing 10% mismatch; (6) duplicated polymerase chain reaction (PCR) sequences. The low-quality bases at heads or tails of reads were also trimmed. After that, the short-insert library reads were corrected using SOAPec [10], a k-mer-based error correction package. This resulted in a 244.84-Gb qualified dataset, representing about 82-fold genome coverage (Table S1).

### Estimation of milu genome size

The milu genome size (G) was estimated by k-mer frequency distribution analysis of the short-insert library, with a 1-bp slide and k set at 17, using the formula G = k-mer_number/k-mer_depth [10]. Here, “k-mer_number” is 1 592 668 741 and the expected “k-mer_depth” is 25 (Fig. S1). The estimated milu genome size with these parameters is about 3.04 Gb (Table S2). For comparison, we also used GCE software and the GenomeScope package to estimate the milu genome size and obtained estimates of 3.00 Gb and 2.78 Gb, respectively [11–13] (Fig. S2). All these estimated genome sizes are within the range of C values (2.22 to 3.44) reported for Cervidae in the Animal Genome Size Database [14], indicating that our estimations are credible (Table S3).

### Genome assembly

SOAPdenovo software, version 2.04 (SOAPdenovo, RRID:SCR_010752) [15], was applied (with parameter settings pregraph-K 79; contig -M 1; scaff –L 200 -b 1.5 -p 40) to construct the original contigs and initial scaffolds using corrected reads for the milu genome assembly. Then we used GapCloser, version 1.12 (GapCloser, RRID:SCR_015026) [10], to fill the gaps of initial scaffolds using short-insert size PE reads (170, 500, and 800 bp). The initial scaffolds were then divided into scaff-tigs by the unfilled gaps. The divided scaff-tigs were connected to final scaffolds using SSPACE, version 3.0 (SSPACE, RRID:SCR_005056) [16], with the following parameters: -x 0, -z 200, -g 2, -k 2, -n 10. These final scaffolds’ gaps were also closed by GapCloser. The total length of our final milu genome assembly is 2.52 Gb, accounting for 85.71% of the estimated genome size. The final contig N50 and scaffold N50 (>2 kb) sizes are 32.71 kb and 3.03 Mb, respectively (Table 1).

**Table 1:** Statistics of the assembled sequence length

	Contig		Scaffold	
	Size, bp	Number	Size, bp	Number
N90	8530	77 768	520 987	978
N80	14 483	55 968	1 045 447	647
N70	20 193	41 646	1 614 103	455
N60	26 169	30 975	2 222 401	322
N50	32 707	22 564	3 039 716	223
Longest	292 964	–	17 945 643	–
Total size	2 460 119 591	–	2 524 831 955	–
Percentage of unknown bases	–	–		2.56%
Total number (≥100 bp)	–	189 067	–	46 381
Total number (≥2 kb)	–	118 986	–	4772

### Quality assessment

To evaluate the quality of the milu genome assembly, the filtered reads (≥49 bp) were aligned to the assembled genome sequences using SOAPaligner, version 2.20 (SOAPaligner/soap2, RRID:SCR_005503) [15], allowing 3 mismatches. We also sequenced the genome of another male milu deer. The clean reads obtained from this sequencing were also aligned to the assembled genome by SOAPaligner with the same parameters. Both alignments showed high coverage of each genome base, confirming accuracy at the base level (Fig. S3 and Fig. S4). In addition, analysis with Benchmarking Universal Single-Copy Orthologs, version 2.0 (BUSCO, RRID:SCR_015008) [17], showed that the assembly included complete matches for 3820 of 4104 mammalian BUSCOs (indicating 93.00% completeness) (Table S4). Feature-Response Curves (FRC; version 1.3.0) [18] was then used to evaluate the trade-off between its contiguity and correctness. FRC generated by the software showed that our milu genome assembly has similar correctness to published genomes of another 3 ruminants: domestic goat (*Capra hircus*, ARS1, GenBank ID: GCF_0 017 04415.1) [19], sheep (*Ovis aries*, Oar_v3.1), and cattle (*Bos taurus* UMD3.1) (Fig. S5) [20, 21]. Subsequently, synteny analysis was applied to identify differences between the assembled genome and the domestic goat (*Capra hircus*) genome using MUMmer (version 3.23) [22], with a 50% identity cutoff for MUMs in the NUCmer alignments used to determine synteny (Fig. S6); 99.35% of the 2 genome sequences could be 1:1 aligned. In addition, we compared the milu and goat genomes using LAST, version 3 (LAST, RRID:SCR_006119) [23], to find the breakpoints (edges of structural variation). The overall density of different types of breakpoints was about 54.76 per Mb, comparable to densities reported in another study (Table S5) [24], and the average nuclear distance (percentage of different base pairs in the syntenic regions) was 6.56% (Fig. S7). The results indicated that the milu genome assembly has good completeness and continuity.

### Repeat annotation

To annotate repeats, we first searched the milu genome for tandem repeats using Tandem Repeats Finder (version 4.04) [25] with the following settings: Match = 2, Mismatch = 7, Delta = 7, PM = 80, PI = 10, Minscore = 50. Then, RepeatMasker version 3.3.0 (RepeatMasker, RRID:SCR_012954) and RepeatProteinMask (version 3.3.0, a package in RepeatMasker) [26] were used to find known transposable element (TE) repeats in the Repbase TE library (version 16.01) [27]. In addition, RepeatModeler version 1.0.5 (RepeatModeler, RRID:SCR_015027) and LTR_FINDER version 1.0.5 (LTR_Finder, RRID:SCR_015247) [28] were used to construct a *de novo* repeat library, and RepeatMasker was employed to find homolog repeats in the genome and classify the detected repeats. The results indicated that long interspersed elements accounted for 27.05% of the milu genome, and other identified repeat sequences accounted for a further 13.99% (Table S6).

### Gene annotation

To annotate structures and functions of putative genes in our milu genome assembly, we used both homology-based and *de novo* predictions. For homology-based predictions, homologous proteins of *Homo sapiens* (Ensembl 89 release), *Bos taurus*, and *Sus scrofa* (Ensembl 89 release) were aligned to the repeat-masked milu genome using TblastN (Blastall 2.2.23) with an E-value cutoff of 1e-5. Then aligned sequences and corresponding query proteins were filtered and passed to GeneWise, version 2.2.0 (GeneWise, RRID:SCR_015054) [29], for accurate spliced alignments. Gene sequences shorter than 150 bp and frame-shifted or prematurely terminated genes were removed. *De novo* predictions were obtained from analysis of the repeat-masked genome using Augustus, version 2.5.5 (Augustus: Gene Prediction, RRID:SCR_008417) [30], and GENSCAN, version 1.0 (GENSCAN, RRID:SCR_012902) [31], with parameters generated from training with *Homo sapiens* genes. The filter processes applied in the homology-based prediction procedure were also applied in the *de novo* predictions. Next, the obtained results were integrated using GLEAN (version 1.0.1) [32], and then genes with few exons (≤3), which could not be aligned well in SwissProt or TrEMBL, were filtered to produce a final consensus gene set containing 20 125 genes. The number of genes, gene length distribution, exon number per gene, and intron length distribution were similar to those of other mammals (Fig. S8 and Table S7). We also identified a total of 2803 pseudogenes from GeneWise alignment, of which 2801 had prematurely terminating mutations and 1358 had frame-shifted mutations (Tables S8 and S9) [29].

Then, the KEGG, SwissProt, and TrEMBL databases were searched for best matches to the final gene set using BLASTP (version 2.2.26) with an E-value of 1e-5. Subsequently, InterProScan software, version 5.18–57.0 (InterProScan, RRID:SCR_005829), was applied to map putative encoded protein sequences against entries in the Pfam, PRINTS, ProDom, and SMART databases to identify known motifs and domains. In total, at least 1 function was allocated to 17 913 (89.31%) of the genes in this manner (Table S10). Next, reads from the short-insert library with about 27-fold genome coverage were mapped to the milu genome using BWA, version 0.7.15-r1140 (BWA, RRID:SCR_010910) [33], and subsequently called variants by SAMtools (version 1.3.1) [34]. Finally, SnpEff (version 4.10) [35] was applied to identify the distribution of single nucleotide variants (SNVs) in the milu genome (Table S11).

In addition, putative short noncoding RNAs were identified by BLASTN alignment of human rRNA sequences with milu homologs. We employed Infernal, version 0.81 (Infernal, RRID:SCR_011809), with the Rfam database (release 9.1) to annotate the miRNA and snRNA genes. The tRNAs were annotated using tRNAscan-SE, version 1.3.1 (tRNAscan-SE, RRID:SCR_010835), with default parameters. In total, 3949 short noncoding RNA sequences were identified in the milu deer genome (Table S12).

### Species-specific genes and phylogenetic relationships

**Figure 2:**
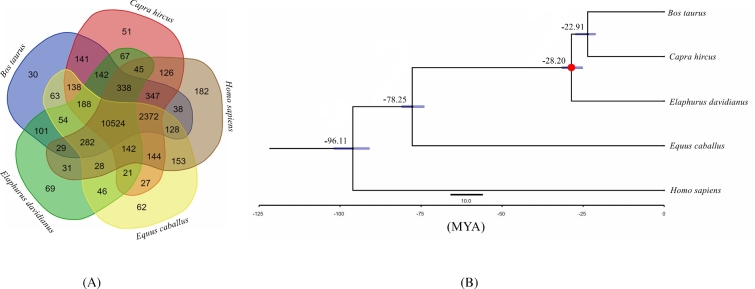
Phylogenetic relationships and genomic comparisons. (A) A Venn diagram of the orthologues shared among *Elaphurus davidianus, Equus caballus, Capra hircus, Bos taurus*, and *Homo sapiens.* Each number represents a gene family number, and the sum of the numbers in the green, yellow, brown, red, and blue areas indicate total numbers of the gene families in milu, horse, human, goat, and cattle genomes, respectively. (B) Divergence time estimates for the 5 species generated using MCMCtree and the 4-fold degenerate sites; the dots correspond to calibration points, and the divergence times were obtained from http://www.timetree.org/; blue nodal bars indicate 95% confidence intervals.

The detected milu genes were clustered in families using OrthoMCL, version 2.0.9 (OrthoMCL DB: Ortholog Groups of Protein Sequences, RRID:SCR_007839) [36], with an E-value cutoff of 1e-5, and Markov Chain Clustering with a default inflation parameter in an all-to-all BLASTP analysis of entries for 5 species (*Homo sapiens, Equus caballus, Capra hircus, Bos taurus*, and *Elaphurus davidianus*). The results indicated that 69 gene families and 167 genes were specific to milu (Fig. 2A, Table S13). We also detected 293 gene families that had apparently expanded in the milu lineage using Computational Analysis of gene Family Evolution (CAFÉ; version 4.0.1) [37]. The milu species-specific gene families were enriched in 4 gene ontology (GO) categories related to hormone activity, pinocytosis, ribosomes, and structural constituents of ribosomes (Table S14). The expanded gene families were enriched in 34 GO categories: motor activity, ATPase activity, calcium ion binding, and 31 others (for details, see Table S15). Subsequently, 7906 1:1 orthologs were identified from these species and aligned using PRANK (version 3.8.31) [38]. Next, we extracted 4D sites (4-fold degenerate sites) to construct a phylogenetic tree by RAxML, version 7.2.8 (RAxML, RRID:SCR_006086) [39], with the GTR+G+I model. Finally, phylogenetic analysis by PAML MCMCtree (version 4.5) [40], calibrated with published timings for the divergence of the reference species [41], showed that *Elaphurus davidianus, Bos taurus*, and *Capra hircus* diverged from a common ancestor approximately 28.20 million years ago (Fig. 2B).

In summary, we report the first sequencing, assembly, and annotation of the milu genome. The assembled draft genome will provide a valuable resource for studying the species’ evolutionary history, as well as genetic changes and associated phenomena, such as genetic load and selection pressures that occurred during its severe bottleneck or other unknown historical events. It should be noted that this draft assembly was generated by next-generation sequencing data and there may be some errors in highly guanosine and cytosine (GC)-biased or repeated regions. Moreover, this genome assembly should be elevated to the chromosomal level in the future with Hi-C, optical mapping, or genetic mapping technologies.

### Supporting data

The raw reads of each sequencing library have been deposited at NCBI, Project ID: PRJNA391565. For the assembled individual (Sample ID: SAMN07270940), please refer to the SRA accession numbers in Table S1. The SRA accession number for the other sequenced individual (Sample ID: SAMN08014286) is SRR6287186. The assembly and annotation of the milu genome, together with further supporting data, are available via the *GigaScience* database, *Giga*DB [42]. Supplementary figures and tables are provided in Additional file 1.

## Additional files

Figure S1: K-mer (k = 25) distribution in the milu genome.

Figure S2: GenomeScope K-mer profile plot of the milu genome.

Figure S3: Sequence depth distribution of the assembly data.

Figure S4: Sequence depth distribution of the assembly data for the genome of the other sequenced individual.

Figure S5: Feature-response curves of 4 ruminant genome assemblies.

Figure S6: Visualized synteny between the milu and goat genomes.

Figure S7: DNA sequence divergence between milu and goat.

Figure S8: Comparison of gene lengths, intron lengths, exon lengths, and exon numbers in milu, cattle, human, and sheep genomes.

Table S1: Summary of sequenced reads.

Table S2: 17-mer depth distribution.

Table S3: Summary of the C values of Cervidae and estimated milu genome sizes.

Table S4: Summary of BUSCO analysis of matches to the 4104 mammalian BUSCOs.

Table S5: Summary of breakpoints between milu and goat genomes.

Table S6: TE contents in the assembled milu genome.

Table S7: General statistics of predicted protein-coding genes.

Table S8: Summary of the predicted pseudogenes.

Table S9: List of predicted pseudogenes.

Table S10: Summary statistics of gene function annotation.

Table S11: Distribution of single nucleotide variants in the milu genome.

Table S12: Summary of short ncRNA annotation.

Table S13: List of milu species-specific genes.

Table S14: GO term enrichment in milu species-specific genes.

Table S15: GO term enrichment in gene families that have expanded in milu.

## Abbreviations

bp: base pair; BUSCO: Benchmarking Universal Single-Copy Orthologs; FRC: feature-response curves; Gb: giga base; GO: gene ontology; kb: kilo base; Mb: mega base; SNV: single nucleotide variant; TE: transposable element.

## Ethics approval

Animal collection and utility protocols were approved by the Northwestern Polytechnical University and BGI-Shenzhen Laboratory Animal Care and Use Committee and were in accordance with guidelines from the China Council on Animal Care. Samples provided by San Diego Zoo Global (SDZG) were collected in accordance with SDZG’s Institutional Animal Care and Use Committee policies, which meet or exceed US regulatory standards for the humane care and treatment of animals in research.

## Competing interests

The authors declare that they have no competing interests.

## Author contributions

Q.Q., W.W., and G.Z. conceived the study. C.Z. and L.C. designed the scientific objectives. L.G.C. and O.A.R. evaluated and provided samples from San Diego Zoo Global. Y.Z. collected the samples, extracted the genomic DNA, and constructed the DNA libraries. C.Z. and Y.Z. estimated the milu genome size and assembled the genome. C.Z., L.C., and Y.Z. carried out the quality assessment, repeat annotation, and gene annotation. C.Z. and K.W. were responsible for finding species-specific genes and phylogenetic relationship construction. L.C. and C.Z. uploaded the raw read data, genome assembly, and annotation in the NCBI and *GigaScience* (*Giga*DB) databases. C.Z., Q.Q., and W.W. wrote the manuscript. Q.Q., W.W., and G.Z. supervised all aspects of the work to ensure the accuracy and integrity of the research and data. O.A.R. contributed to editing the final manuscript. All authors read and approved the final manuscript.

## Supplementary Material

GIGA-D-17-00161_Original_Submission.pdf

GIGA-D-17-00161_Revision_1.pdf

GIGA-D-17-00161_Revision_2.pdf

Response_to_Reviewer_Comments_Original_Submission.pdf

Response_to_Reviewer_Comments_Revision_1.pdf

Reviewer_1_Report_(Original_Submission) -- Derek Bickhart 11 Jul 2017 Reviewed

Reviewer_1_Report_(Revision_1) -- Derek Bickhart 24 Oct 2017 Reviewed

Reviewer_2_Report_(Original_Submission) -- Jane Loveland 17 Jul 2017 Reviewed

Reviewer_2_Report_(Revision_1) -- Jane Loveland 06 Nov 2017 Reviewed

Supplemental Figures and Tables

## References

[1] Harrison RJ, Hamilton WJ The reproductive tract and the placenta and membranes of Père David's deer (*Elaphurus davidianus* Milne Edwards). J Anat 1952;86(2):203–25. 14946073 PMC1273772

[2] Cao K On the time of extinction of the wild Mi-deer in China [Chinese]. Acta Zoologica Sinica 1978;24(3):289–91.

[3] JONES F A contribution to the history and anatomy of Père David's Deer (*Elaphurus davidianus*). J Zool 1951;2(121):319–70.

[4] Ding Y Chinese Milu Research [Chinese]. Changchun, China: Jilin Publishing House for the Science and Technology; 2004.

[5] Tate ML, Mathias HC, Fennessy PF A new gene mapping resource: interspecies hybrids between Pere David's deer (*Elaphurus davidianus*) and red deer (*Cervus elaphus*). Genetics 1995;139(3):1383–91. 7768446 10.1093/genetics/139.3.1383 PMC1206464

[6] Slate J, Van Stijn TC, Anderson RM A deer (subfamily Cervinae) genetic linkage map and the evolution of ruminant genomes. Genetics 2002;160(4):1587–97. 11973312 10.1093/genetics/160.4.1587 PMC1462045

[7] Pitra C, Fickel J, Meijaard E Evolution and phylogeny of old world deer. Mol Phylogenet Evol 2004;33(3):880–95. 15522810 10.1016/j.ympev.2004.07.013

[8] Maqbool NJ, Tate ML, Dodds KG A QTL study of growth and body shape in the inter-species hybrid of Pere David's deer (*Elaphurus davidianus*) and red deer (*Cervus elaphus*). Anim Genet 2007;38(3):270–6. 17433011 10.1111/j.1365-2052.2007.01597.x

[9] Emerson BC, Tate ML Genetic analysis of evolutionary relationships among deer (subfamily Cervinae). J Hered 1993;84(4):266–73. 8340615 10.1093/oxfordjournals.jhered.a111338

[10] Li R, Fan W, Tian G The sequence and de novo assembly of the giant panda genome. Nature 2010;463(7279):311–7. 20010809 10.1038/nature08696 PMC3951497

[11] Liu B, Shi Y, Fan W Estimation of genomic characteristics by analyzing k-mer frequency in de novo estimation of genomic characteristics by analyzing k-mer frequency in de novo genome projects. arXiv preprint 2013 arXiv:1308.2012.

[12] Vurture GW, Sedlazeck FJ, Nattestad M GenomeScope: fast reference-free genome profiling from short reads. Bioinformatics 2017;33(14):2202–4. 28369201 10.1093/bioinformatics/btx153 PMC5870704

[13] Marcais G, Kingsford C A fast, lock-free approach for efficient parallel counting of occurrences of k-mers. Bioinformatics 2011;27(6):764–70. 21217122 10.1093/bioinformatics/btr011 PMC3051319

[14] Gregory TR Animal Genome Size Database. 2017 http://www.genomesize.com.

[15] Li R, Zhu H, Ruan J De novo assembly of human genomes with massively parallel short read sequencing. Genome Res 2010;20(2):265–72. 20019144 10.1101/gr.097261.109 PMC2813482

[16] Boetzer M, Henkel CV, Jansen HJ Scaffolding pre-assembled contigs using SSPACE. Bioinformatics 2011;27(4):578–9. 21149342 10.1093/bioinformatics/btq683

[17] Simao FA, Waterhouse RM, Ioannidis P BUSCO: assessing genome assembly and annotation completeness with single-copy orthologs. Bioinformatics 2015;31(19):3210–2. 26059717 10.1093/bioinformatics/btv351

[18] Vezzi F, Narzisi G, Mishra B Reevaluating assembly evaluations with feature response curves: GAGE and assemblathons. Plos One 2012;7(12):e52210. 23284938 10.1371/journal.pone.0052210 PMC3532452

[19] Bickhart DM, Rosen BD, Koren S Single-molecule sequencing and chromatin conformation capture enable de novo reference assembly of the domestic goat genome. Nat Genet 2017;49(4):643–50. 28263316 10.1038/ng.3802 PMC5909822

[20] Jiang Y, Xie M, Chen W The sheep genome illuminates biology of the rumen and lipid metabolism. Science 2014;344(6188):1168–73. 24904168 10.1126/science.1252806 PMC4157056

[21] Elsik CG, Tellam RL, Worley KC The genome sequence of taurine cattle: a window to ruminant biology and evolution. Science 2009;324(5926):522–8. 19390049 10.1126/science.1169588 PMC2943200

[22] Delcher AL, Salzberg SL, Phillippy AM Using MUMmer to identify similar regions in large sequence sets. Curr Protoc Bioinformatics 2003;Chapter 10:10–13. 10.1002/0471250953.bi1003s00 18428693

[23] Kielbasa SM, Wan R, Sato K Adaptive seeds tame genomic sequence comparison. Genome Res 2011;21(3):487–93. 21209072 10.1101/gr.113985.110 PMC3044862

[24] Wang K, Wang L, Lenstra JA The genome sequence of the wisent (*Bison bonasus*). Gigascience 2017;6(4):1–5. 10.1093/gigascience/gix016 PMC5530314 28327911

[25] Benson G. Tandem repeats finder: a program to analyze DNA sequences. Nucleic Acids Res 1999;27(2):573–80. 9862982 10.1093/nar/27.2.573 PMC148217

[26] Tarailo-Graovac M, Chen N Using RepeatMasker to identify repetitive elements in genomic sequences. Curr Protoc Bioinformatics 2009;Chapter 4:4–10. 10.1002/0471250953.bi0410s25 19274634

[27] Jurka J, Kapitonov VV, Pavlicek A Repbase Update, a database of eukaryotic repetitive elements. Cytogenet Genome Res 2005;110(1–4):462–7. 16093699 10.1159/000084979

[28] Xu Z, Wang H LTR_FINDER: an efficient tool for the prediction of full-length LTR retrotransposons. Nucleic Acids Res 2007;35(Web Server):W265–8. 17485477 10.1093/nar/gkm286 PMC1933203

[29] Birney E, Clamp M, Durbin R GeneWise and Genomewise. Genome Res 2004;14(5):988–95. 15123596 10.1101/gr.1865504 PMC479130

[30] Stanke M, Keller O, Gunduz I AUGUSTUS: ab initio prediction of alternative transcripts. Nucleic Acids Res 2006;34(Web Server):W435–9. 16845043 10.1093/nar/gkl200 PMC1538822

[31] Burge C, Karlin S Prediction of complete gene structures in human genomic DNA. J Mol Biol 1997;268(1):78–94. 9149143 10.1006/jmbi.1997.0951

[32] Elsik CG, Mackey AJ, Reese JT Creating a honey bee consensus gene set. Genome Biol 2007;8(1):R13. 17241472 10.1186/gb-2007-8-1-r13 PMC1839126

[33] Li H Aligning sequence reads, clone sequences and assembly contigs with BWA-MEM. arXiv preprint 2013 arXiv:13033997.

[34] Li H, Handsaker B, Wysoker A The Sequence Alignment/Map format and SAMtools. Bioinformatics 2009;25(16):2078–9. 19505943 10.1093/bioinformatics/btp352 PMC2723002

[35] Cingolani P, Platts A, Wang LL A program for annotating and predicting the effects of single nucleotide polymorphisms, SnpEff. Fly 2012;6(2):80–92. 22728672 10.4161/fly.19695 PMC3679285

[36] Li L, Stoeckert CJ, Roos DS OrthoMCL: identification of ortholog groups for eukaryotic genomes. Genome Res 2003;13(9):2178–89. 12952885 10.1101/gr.1224503 PMC403725

[37] De Bie T, Cristianini N, Demuth JP CAFE: a computational tool for the study of gene family evolution. Bioinformatics 2006;22(10):1269–71. 16543274 10.1093/bioinformatics/btl097

[38] Loytynoja A, Goldman N From the cover: an algorithm for progressive multiple alignment of sequences with insertions. Proc Natl Acad Sci U S A 2005;102(30):10557–62. 16000407 10.1073/pnas.0409137102 PMC1180752

[39] Stamatakis A. RAxML version 8: a tool for phylogenetic analysis and post-analysis of large phylogenies. Bioinformatics 2014;30(9):1312–3. 24451623 10.1093/bioinformatics/btu033 PMC3998144

[40] Yang Z. PAML 4: phylogenetic analysis by maximum likelihood. Mol Biol Evol 2007;24(8):1586–91. 17483113 10.1093/molbev/msm088

[41] Kumar S, Stecher G, Suleski M, Hedges SB TimeTree: A Resource for Timelines, Timetrees, and Divergence Times. Mol Biol Evol 2017;34(7):1812–9. 28387841 10.1093/molbev/msx116

[42] Zhang C, Chen L, Zhou Y Supporting data for “Draft genome of the milu (*Elaphurus davidianus*).” GigaScience Database 2017 http://dx.doi.org/10.5524/100383. 10.1093/gigascience/gix130 PMC5824821 29267854

