# Supplementary material for: Draft genome of the milu (Elaphurus davidianus)
Source: Gigascience. 2017 Dec 18;7(2):gix130. doi: 10.1093/gigascience/gix130 (PMC5824821; doi:10.1093/gigascience/gix130)
Supplement: GIGA-D-17-00161_Original_Submission.pdf [file gix130_giga-d-17-00161_original_submission.pdf]

GigaScience

Draft genome of the milu (Elaphurus davidianus)

--Manuscript Draft--

**Manuscript Number:**	GIGA-D-17-00161
**Full Title:**	Draft genome of the milu (Elaphurus davidianus)
**Article Type:**	Data Note
**Funding Information:**	Talents Team Construction Fund of Northwestern Polytechnical University (NWPU) — Dr. Wen Wang<br>Dr. Qiang Qiu
**Abstract:**	Abstract<br>Background: Milu, also known as Père David's deer (Elaphurus davidianus), had been widely distributed in East Asia but experienced sever bottleneck (only 18 survived by the end of 19th century), and the current 4500 individual population was propagated from only 11 individuals reared by the 11th British Duke of Bedford. This species is known for its distinguishable appearance, the driving force behind which is still a mystery. The draft genome reported in this study will provide valuable resources to investigate deeper into its evolutionary history and population dynamics of severely bottlenecked species.<br>Findings: In total, we generated 321.86 gigabases (Gb) of raw DNA sequence from whole-genome sequencing of the male milu deer using an Illumina HiSeq 2000 platform. Assembly gave a final genome with scaffold N50 of 3.03 megabases (Mb), and total length of 2.5 Gb. Moreover, we identified 20,521 protein-coding genes and 988.1 Mb of repetitive sequences. In addition, homology-based searches detected 280 rRNA, 1,335 miRNA, 1,441 snRNA and 893 tRNA sequences in the genome of E. davidianus. The divergence time between E. davidianus. and ancestors Bos taurus and Capra hircus, was estimated to be 28.60 million years ago.<br>Conclusions: We report the first reference genome of milu. The genome will provide a valuable resource for studying the species' demographic history and the population genetic dynamics for severely bottlenecked species.<br><br>Keywords: Elaphurus davidianus, Reference genome, Evolution
**Corresponding Author:**	Qiang Qiu, Ph.D.<br><br>CHINA
**Corresponding Author Secondary Information:**	
**Corresponding Author's Institution:**	
**Corresponding Author's Secondary Institution:**	
**First Author:**	Chenzhou Zhang
**First Author Secondary Information:**	
**Order of Authors:**	Chenzhou Zhang
	Lei Chen
	Yang Zhou
	Kun Wang
	Wen Wang
	Guojie Zhang
	Qiang Qiu, Ph.D.
**Order of Authors Secondary Information:**	
**Opposed Reviewers:**	Lifeng Zhu, Ph.D.

Powered by Editorial Manager® and ProduXion Manager® from Aries Systems Corporation

	Nanjing Normal University<br>zhulf@ioz.ac.cn
	Guoqing Lu, Ph.D.<br>University of Nebraska at Omaha<br>glu3@unomaha.edu
	Jianquan Liu, Ph.D.<br>Lanzhou University<br>liujq@lzu.edu.cn

**Additional Information:**	
**Question**	**Response**
Are you submitting this manuscript to a special series or article collection?	No
**Experimental design and statistics**<br><br>Full details of the experimental design and statistical methods used should be given in the Methods section, as detailed in our Minimum Standards Reporting Checklist. Information essential to interpreting the data presented should be made available in the figure legends.<br><br>Have you included all the information requested in your manuscript?	Yes
**Resources**<br><br>A description of all resources used, including antibodies, cell lines, animals and software tools, with enough information to allow them to be uniquely identified, should be included in the Methods section. Authors are strongly encouraged to cite Research Resource Identifiers (RRIDs) for antibodies, model organisms and tools, where possible.<br><br>Have you included the information requested as detailed in our Minimum Standards Reporting Checklist?	Yes
**Availability of data and materials**<br><br>All datasets and code on which the conclusions of the paper rely must be either included in your submission or deposited in publicly available repositories (where available and ethically appropriate), referencing such data using a unique identifier in the references and in the "Availability of Data and Materials" section of your manuscript.	Yes

Have you have met the above requirement as detailed in our Minimum Standards Reporting Checklist?	

Manuscript Click here to download Manuscript MS-Milu.docx

Draft genome of the milu (*Elaphurus davidianus*)

Chenzhou Zhang[1, †], Lei Chen[1, †], Yang Zhou[2, 3, †], Kun Wang[1], Wen Wang[1, *], Guojie Zhang[2, 3, 4, *], Qiang Qiu[1, *]

[1] Center for Ecological and Environmental Sciences, Key Laboratory for Space Bioscience & Biotechnology, Northwestern Polytechnical University, Xi'an, 710072, China

[2] China National Genebank, BGI-Shenzhen, Shenzhen 518083, China

[3] BGI-Shenzhen, Shenzhen 518083, China

[4] Centre for Social Evolution, Department of Biology, Universitetsparken 15, University of Copenhagen, Copenhagen 2100, Denmark

*Correspondence: qiuqiang@lzu.edu.cn (QQ), wwang@mail.kiz.ac.cn (WW), zhanggj@genomics.cn (GZ)

†These authors contributed equally to this work.

Abstract

Background: Milu, also known as Père David's deer (*Elaphurus davidianus*), had been widely distributed in East Asia but experienced sever bottleneck (only 18 survived by the end of 19th century), and the current 4500 individual population was propagated from only 11 individuals reared by the 11th British Duke of Bedford. This species is known for its distinguishable appearance, the driving force behind which is still a mystery. The draft genome reported in this study will provide valuable resources to investigate deeper into its evolutionary history and population dynamics of severely bottlenecked species.

Findings: In total, we generated 321.86 gigabases (Gb) of raw DNA sequence from whole-genome sequencing of the male milu deer using an Illumina HiSeq 2000 platform. Assembly gave a final genome with scaffold N50 of 3.03 megabases (Mb), and total length of 2.5 Gb. Moreover, we identified 20,521 protein-coding genes and 988.1 Mb of repetitive sequences. In addition, homology-based searches detected 280 rRNA, 1,335 miRNA, 1,441 snRNA and 893 tRNA sequences in the genome of *E. davidianus.* The divergence time between *E. davidianus.* and ancestors *Bos taurus* and *Capra hircus*, was estimated to be 28.60 million years ago.

Conclusions: We report the first reference genome of milu. The genome will provide a valuable resource for studying the species' demographic history and the population genetic dynamics for severely bottlenecked species.

Keywords: *Elaphurus davidianus*, Reference genome, Evolution

Data description

Background

Père David's deer (*Elaphurus davidianus*), named after its western finder (Father *Armand David*) and called "milu" in China, was an endemic species that was once widely distributed in East Asia[1, 2]. Milu also has an informal Chinese name "*Sibuxiang*", meaning its unusual appearance which could be explained by four distinct characteristics, including the antlers similar to genus *Cervus*, the head similar to horse, the similar hoofs with cow, and the tail like to donkey (**Fig. 1**). Due to intense human and natural pressures, milu became extinct in China by the end of the 19th century and only 18 individuals survived in several European zoos at that time, all of which were subsequently collected by the 11th British Duke of Bedford and kept at Woburn Abbey (UK) and 11 of the 18 milu took part in subsequent reproduction [3]. After this severe bottle-neck, the milu population started to recover. In the 1980s, dozens were reintroduced into China, and there were over 1,500 in China and more than 3,000 worldwide by 2004 [4]. As a survived cervid that luckily escaped extinction, milu has highly specialized traits compared with other Cervinae species such as relatively long tail and unique branched antler. Due to these specific characteristics, scientists once identified it as the root of the subfamily Cervinae, but subsequent molecular analysis indicated that milu is closer to genus *Cervus* [5-9]. However, little is still known about the genetic architecture underlying the special phenotypes and the population genetic dynamics during the milu's recovery from the severe bottleneck. A good quality reference genome of milu will provide an opportunity to investigate effects of the severe recent bottleneck and the molecular mechanism of special phenotypic evolution.

Library construction, and filtering

Genomic DNA was extracted from a male milu tissue obtained from the San Diego Zoo, USA (NCBI Taxonomy ID, 43332). The extracted DNA was used to construct short-insert libraries (170, 500 and 800 base pair, bp) and subsequently long-insert libraries (2, 5, 10 and 20 kilo base, kb). After that, a HiSeq 2000 platform (Illumina; CA, USA) was used to sequence paired end reads of each library based on a whole genome shotgun sequencing strategy, generating 49 bp and 100 bp reads from the short-insert and long-insert libraries, respectively. In total, 321.86 Gb raw data was obtained.

Raw reads were filtered according to the following criteria: (1) reads had > 5% uncalled ("N") bases or polyA structure; (2) reads had 30 and 60 bases with quality scores ≤ 7 for reads generated from the long-insert and short-library sequences, respectively; (3) more than 10 bp aligned to the adapter sequence; (4) read1 and read2 (of a short-insert PE read) overlapped by ≥ 10bp, allowing 10% mismatch; (5) duplicated PCR sequences. The low quality bases at heads or tails of reads were also trimmed. Then, a 249.01 Gb clean data was obtained, representing about 100-fold genome coverage. After that, the short-insert library reads were corrected using SOAPec [10], a k-mer-based error correction package.

Estimation of milu genome size

The milu genome size (G) was estimated by K-mer frequency distribution analysis of the short-insert libraries, with a 1-bp slide and k set at 17, using the formula G = k-

mer_number/k-mer_depth [11]. Here, N is 1,592,668,741 and the expected K_depth is 25 (**Fig. S1**). The estimated milu genome size, with these parameters, is 3.04 Gb (**Table S1**).

Genome assembly

SOAPdenovo software (version 2.04) [12] with parameters (pregraph-K 79; contig -M 1; scaff –L 200 -b 1.5 -p 40) were applied to construct the original contigs and scaffolds using corrected reads for the assembly of milu genome. Then we used GapCloser (version 1.12) [11] to fill the gaps using short-insert sizes PE reads (170, 500 and 800 bp). Next, the resulting gap-filled scaffolds were split into contigs. Subsequently, SSPACE (version 3.0) [13] was employed to build scaffolds using the contigs and all corrected reads with the following parameters: -x 0, -z 200, -g 2, -k 2, -n 10. These scaffolds' gaps were also closed by GapCloser. The total length of our final milu genome assembly is 2.52 Gb accounting for 83.05 % of the estimated genome size. The contig N50 and scaffold N50 (>2 kb) sizes are 32.71 kb and 3.03 Mb, respectively (**Table 1**).

Quality assessment

To evaluate the quality of the milu genome assembly, the filtered reads (≥ 49 bp) were aligned to the assembled genome sequences using SOAPaligner (version 2.20) [12] allowing 3 mismatches. The high coverage of each genome base ensured the accuracy at base level (**Fig. S2**). In addition, analysis with BUSCO (benchmarking universal

single-copy orthologs, version 2.0) [14] showed that the assembly included complete matches for 3820 of 4104 mammalian BUSCOs (indicating 93 % completeness) (**Table S2**). FRC (Feature-response curves, version 1.3.0) [15] was then used to evaluate the trade-off between its contiguity and correctness. FRC curves generated by the software showed that our milu genome assembly has similar correctness to the published genomes of another three ruminants: domestic goat (*Capra hircus*), sheep (*Ovis aries* Oar_v3.1) and cattle (*Bos taurus* UMD3.1) (**Fig. S3**). Subsequently, synteny analysis was applied to identify differences between the assembled genome and the domestic goat (*Capra hircus*) genome using MUMmer (version 3.23) [16] **(Fig. S4**). 99.35% of two genome sequences could be 1:1 aligned. In addition, the density of different types of break points (edges of structural variation) are about 54.76 per Mb (**Table S3**) which was compared by LAST (version 3) and the average nuclear distance (percentage of different base pairs in the syntenic regions) was 6.56% (**Fig. S5**). The results indicated that the milu genome assembly has good completeness and continuity.

Repeat annotation

To annotate repeats, we first searched the milu genome for tandem repeats using Tandem Repeats Finder (version 4.04) [17] with the following settings: Match = 2, Mismatch = 7, Delta = 7, PM = 80, PI = 10, Minscore = 50. Then, RepeatMasker (version 3.3.0) and RepeatProteinMask (version 3.3.0, a package within RepeatMasker) [18] were used to find known transposable element (TE) repeats in the Repbase TE library (version 16.01) [19]. In addition, RepeatModeler (version 1.0.5) and

LTR_FINDER (version 1.0.5) [20] were used to construct a *de novo* repeat library and RepeatMasker was employed to find homolog repeats in the genome and to classify the detected repeats. The results indicated that the identified repeat sequences accounted for 41.04 % milu genome, and predominated by the long interspersed elements (27.05 %) (**Table S4**).

Gene annotation

To annotate structures and functions of putative genes in our milu genome assembly we used both homology-based and *de novo* predictions. For homology-based predictions, homologous proteins of *Homo sapiens* (Ensembl 60 release), *Bos taurus* and *Sus scrofa* (Ensembl 63 release) were aligned to the repeat-masked milu genome using TblastN (Blastall 2.2.23) with an E-value cutoff 1e-5. Then aligned sequences and corresponding query proteins were filtered and passed to GeneWise (version 2.2.0) [21] for accurate spliced alignments. Gene sequences shorter than 150 bp, and frame-shifted or prematurely terminated genes, were removed. *De novo* predictions were obtained from analysis of the repeat-masked genome using Augustus (version 2.5.5) [22] and Genscan (version 1.0) [23], with parameters generated from training with *Homo sapiens* genes. The filter processes applied in the homology-based prediction procedure were also applied in the *de novo* predictions. Next, the obtained results were integrated using GLEAN (version 1.0.1) [24], then genes with few exons (≤ 3), which could not be aligned well in SwissProt or TrEMBL were filtered to produce a final consensus gene set containing 20,521 genes (**Table S5**).

Then, the KEGG, SwissProt and TrEMBL databases were searched for best matches to the final gene set using BLASTP (version 2.2.26) with an E-value of 1e-5. Subsequently, InterProScan software (version 5.18-57.0) was applied to map putative encoded protein sequences against entries in the Pfam, PRINTS, ProDom and SMART databases to identify known motifs and domains. In total, at least one function was allocated to 19,875 (96.85%) of the genes in this manner (**Table S6**). Next, about 36-fold genome coverage reads form short-insert libraries were mapped to the milu genome with BWA (version 0.7.15-r1140) [25] and called variants by SAMtools (version 1.3.1) [26] subsequently. Finally, SnpEff (version 4.10) [27] was applied to identify the distribution of single nucleotide variant (SNV) in the milu genome (**Table S7**).

In addition, putative noncoding RNAs were identified by BLASTN alignment of human rRNA sequences with milu homologs. We employed Infernal (version 0.81) with Rfam database (release 9.1) to annotate the miRNA and snRNA genes. The tRNAs were annotated using tRNAscan-SE (version 1.3.1) software with default parameters. In total, 3,949 noncoding RNA sequences were identified in the milu deer genome (**Table S8**).

Species-specific genes and phylogenetic relationship

The detected milu genes were clustered in families by employing OrthoMCL (version 2.0.9) [28] with an E-value cutoff of 1e-5, and a Markov Chain Clustering with default inflation parameter in an all-to-all BLASTP analysis of entries for five species (*Homo sapiens, Equus caballus, Capra hircus, Bos taurus*, and *Elaphurus davidianus*). The

result indicated that 96 gene families were specific to milu (**Fig. 2a**). Subsequently, 8,062 one to one single orthologs were identified from these species and were aligned using PRANK (version 3.8.31) [29]. Next, we extracted 4D-sites (four-fold degenerated sites) to construct a phylogenetic tree by RAxML (version 7.2.8) [30] with GTR+G+I model. Finally, phylogenetic analysis applying PAML MCMCtree (version 4.5) [31], calibrated with published timings of the divergence of the reference species (http://www.timetree.org/), showed that *Elaphurus davidianus*, *Bos taurus* and *Capra hircus* diverged from a common ancestor approximately 28.60 million years ago (**Fig. 2b**).

In summary, we report the first sequencing, assembly, and annotation of the milu genome. The assembled draft genome will provide a valuable resource for studying the species' evolutional history, as well as genetic changes and associated phenomena, such as genetic load and selection pressures that occurred during its severe bottle-neck period or other unknown historical events.

Supporting data

The raw reads of each sequencing library have been deposited at NCBI with the project ID: PRJNA391565, Sample ID: SAMN07270940 and the Genome Sequence Archive [32] under BIG Data Center [33], Beijing Institute Genomics (BIG), Chinese Academy of Science, Project ID: PRJCA000448, Sample ID: SAMC013083. The assembly and

annotation of the milu genome are available in the the *GigaScience* GigaDB database. Supplementary Figures and Tables are provided in Additional file 1.

Abbreviations

Gb: giga base; bp: base pair; kb: kilo base; Mb: mega base; TE: transposable element; BUSCO: benchmarking universal single-copy orthologs; FRC: feature-response curves; SNV: single nucleotide variant;

Acknowledgements

This study was supported by Talents Team Construction Fund of Northwestern Polytechnical University (NWPU) to QQ and WW. We thank Nowbio Biotech lnc,. Kunming, China for the remarkable work on DNA libraries constructions and the assistance during the genome sequencing.

Authors' contributions

LC and KW designed the study. YZ collected the samples and extracted the genomic DNA. YZ, KW and CZ conducted the genome analysis. QQ, WW and GZ wrote the paper. All authors read and approved the final manuscript.

Competing interests

The authors declare that they have no competing interests

References:

1. Harrison RJ, Hamilton WJ. The reproductive tract and the placenta and membranes of Père David's deer (*Elaphurus davidianus* Milne Edwards). Journal of Anatomy. 1952;86 2:203-225.
2. Cao K. On the time of extinction of the wild Mi-deer in China (in Chinese). ACTA ZOOLOGICA SINICA. 1978;24 3:289-291.
3. JONES F. A contribution to the history and anatomy of Père David's Deer (*Elaphurus davidianus*). Journal of Zoology. 1951;2 121:319-370, doi:10.1111/j.1096-3642.1951.tb00800.x.
4. Ding Y. Chinese milu research (in Chinese). Changchun, China: Jilin Publishing House for the Science and Technology; 2004.
5. Tate ML, Mathias HC, Fennessy PF, Dodds KG, Penty JM, Hill DF. A new gene mapping resource: interspecies hybrids between Pere David's deer (*Elaphurus davidianus*) and red deer (*Cervus elaphus*). Genetics. 1995;139 3:1383-1391.
6. Slate J, Van Stijn TC, Anderson RM, McEwan KM, Maqbool NJ, Mathias HC, Bixley MJ, Stevens DR, Molenaar AJ, Beever JE *et al*. A deer (subfamily Cervinae) genetic linkage map and the evolution of ruminant genomes. Genetics. 2002;160 4:1587-1597.
7. Pitra C, Fickel J, Meijaard E, Groves PC. Evolution and phylogeny of old world deer. Molecular Phylogenetics and Evolution. 2004;33 3:880-895, doi:10.1016/j.ympev.2004.07.013.
8. Maqbool NJ, Tate ML, Dodds KG, Anderson RM, McEwan KM, Mathias HC, McEwan JC, Hall RJ. A QTL study of growth and body shape in the inter-species hybrid of Pere David's deer (*Elaphurus davidianus*) and red deer (*Cervus elaphus*). Animal Genetics. 2007;38 3:270-276, doi:10.1111/j.1365-2052.2007.01597.x.
9. Emerson BC, Tate ML. Genetic analysis of evolutionary relationships among deer (subfamily Cervinae). Journal of Heredity. 1993;84 4:266-273.
10. Luo R, Liu B, Xie Y, Li Z, Huang W, Yuan J, He G, Chen Y, Pan Q, Liu Y *et al*. SOAPdenovo2: an empirically improved memory-efficient short-read *de novo* assembler. Gigascience. 2012;1 1:18, doi:10.1186/2047-217X-1-18.
11. Li R, Fan W, Tian G, Zhu H, He L, Cai J, Huang Q, Cai Q, Li B, Bai Y *et al*. The sequence and *de novo* assembly of the giant panda genome. Nature. 2010;463 7279:311-317, doi:10.1038/nature08696.
12. Li R, Zhu H, Ruan J, Qian W, Fang X, Shi Z, Li Y, Li S, Shan G, Kristiansen K *et al*. *De novo* assembly of human genomes with massively parallel short read sequencing. Genome Research. 2010;20 2:265-272, doi:10.1101/gr.097261.109.
13. Boetzer M, Henkel CV, Jansen HJ, Butler D, Pirovano W. Scaffolding pre-assembled contigs using SSPACE. Bioinformatics. 2011;27 4:578-579, doi:10.1093/bioinformatics/btq683.
14. Simao FA, Waterhouse RM, Ioannidis P, Kriventseva EV, Zdobnov EM. BUSCO: assessing genome assembly and annotation completeness with

single-copy orthologs. Bioinformatics. 2015;31 19:3210-3212, doi:10.1093/bioinformatics/btv351.
15. Vezzi F, Narzisi G, Mishra B. Reevaluating assembly evaluations with feature response curves: GAGE and assemblathons. PLoS One. 2012;7 12:e52210, doi:10.1371/journal.pone.0052210.
16. Delcher AL, Salzberg SL, Phillippy AM: Using MUMmer to identify similar regions in large sequence sets. Curr Protoc Bioinformatics. 2003;Chapter 10:10-13, doi:10.1002/0471250953.bi1003s00.
17. Benson G. Tandem repeats finder: a program to analyze DNA sequences. Nucleic Acids Research. 1999;27 2:573-580.
18. Tarailo-Graovac M, Chen N. Using RepeatMasker to identify repetitive elements in genomic sequences. Curr Protoc Bioinformatics. 2009;Chapter 4:4-10, doi:10.1002/0471250953.bi0410s25.
19. Jurka J, Kapitonov VV, Pavlicek A, Klonowski P, Kohany O, Walichiewicz J. Repbase Update, a database of eukaryotic repetitive elements. Cytogenetic and Genome Research. 2005;110 1-4:462-467, doi:10.1159/000084979.
20. Xu Z, Wang H. LTR_FINDER an efficient tool for the prediction of full-length LTR retrotransposons. Nucleic Acids Research. 2007;35 Web Server issue:W265-W268, doi:10.1093/nar/gkm286.
21. Birney E, Clamp M, Durbin R. GeneWise and Genomewise. Genome Research. 2004;14 5:988-995, doi:10.1101/gr.1865504.
22. Stanke M, Keller O, Gunduz I, Hayes A, Waack S, Morgenstern B. AUGUSTUS: ab initio prediction of alternative transcripts. Nucleic Acids Research. 2006;34 Web Server issue:W435-W439, doi:10.1093/nar/gkl200.
23. Burge C, Karlin S. Prediction of complete gene structures in human genomic DNA. Journal of Molecular Biology. 1997;268 1:78-94, doi:10.1006/jmbi.1997.0951.
24. Elsik CG, Mackey AJ, Reese JT, Milshina NV, Roos DS, Weinstock GM. Creating a honey bee consensus gene set. Genome Biology. 2007;8 1:R13, doi:10.1186/gb-2007-8-1-r13.
25. Li H. Aligning sequence reads, clone sequences and assembly contigs with BWA-MEM. arXiv preprint arXiv:13033997. 2013.
26. Li H, Handsaker B, Wysoker A, Fennell T, Ruan J, Homer N, Marth G, Abecasis G, Durbin R. The Sequence Alignment/Map format and SAMtools. Bioinformatics. 2009;25 16:2078-2079, doi:10.1093/bioinformatics/btp352.
27. Cingolani P, Platts A, Wang LL, Coon M, Nguyen T, Wang L, Land SJ, Lu X, Ruden DM. A program for annotating and predicting the effects of single nucleotide polymorphisms, SnpEff: SNPs in the genome of Drosophila melanogaster strain w1118; iso-2; iso-3. Fly (Austin). 2012;6 2:80-92, doi:10.4161/fly.19695.
28. Li L, Stoeckert CJ, Roos DS. OrthoMCL: identification of ortholog groups for eukaryotic genomes. Genome Research. 2003;13 9:2178-2189, doi:10.1101/gr.1224503.
29. Loytynoja A, Goldman N. An algorithm for progressive multiple alignment of

sequences with insertions. Proc Natl Acad Sci U S A. 2005;102 30:10557-10562, doi:10.1073/pnas.0409137102.

30. Stamatakis A. RAxML version 8: a tool for phylogenetic analysis and post-analysis of large phylogenies. Bioinformatics. 2014;30 9:1312-1313, doi:10.1093/bioinformatics/btu033.

31. Yang Z. PAML 4: phylogenetic analysis by maximum likelihood. Molecular Biology and Evolution. 2007;24 8:1586-1591, doi:10.1093/molbev/msm088.

32. Wang Y, Song F, Zhu J, Zhang S, Yang Y, Chen T, Tang B, Dong L, Ding N, Zhang Q *et al*. GSA: Genome Sequence Archive. Genomics Proteomics Bioinformatics. 2017;15 1:14-18, doi:10.1016/j.gpb.2017.01.001.

33. Members BIGDC. The BIG Data Center: from deposition to integration to translation. Nucleic Acids Research. 2017;45 D1:D18-D24, doi:10.1093/nar/gkw1060.

Figure legends

Figure 1: Photo of two fighting Père David's deer in Dafeng Milu National Reserves, Jiangsu, China. A red wound was spotted on the body of the right one and the winner could get the most mating chance.

Figure 2. Phylogenetic relationships and genomic comparisons between *Elaphurus davidianus. Equus caballus, Capra hircus, Bos taurus*, and *Homo sapiens.* (a) A Veen diagram of the shared orthologues among *Elaphurus davidianus. Equus caballus, Capra hircus, Bos taurus* and *Homo sapiens.* Each number represents a gene family number. (b) Divergence time estimates for the five species generated using MCMCtree and the 4-fold degenerate sites; the dots correspond to calibration points and the divergence times were obtained from http:// www.timetree.org/; blue nodal bars indicate 95 % confidence intervals.

Table 1: Statistics of the assembled sequence length.

	Contig		Scaffold	
	**Size(bp)**	**Number**	**Size(bp)**	**Number**
**N90**	8,530	77,768	520,987	978
**N80**	14,483	55,968	1,045,447	647
**N70**	20,193	41,646	1,614,103	455
**N60**	26,169	30,975	2,222,401	322
**N50**	32,707	22,564	3,039,716	223
**Longest**	292,964	----	17,945,643	----
**Total Size**	2,460,119,591	----	2,524,831,955	----
**Percent of unknown bases**	----	----	2.56 %	
**TotalNumber(>=100bp)**	----	189,067	----	46,381
**Total Number(>=2kb)**	----	118,986	----	4,772

Additional files

Figure S1: K-mer (k=25) distribution in the milu genome.

Figure S2: Sequence depth distribution.

Figure S3: Feature-response (FR) curves of four ruminant genome assemblies.

Figure S4: Visualized synteny between the milu and goat genome.

Figure S5: DNA sequence divergence between milu and goat.

Figure S6: Comparison of gene lengths, intron lengths, exon lengths and exon numbers in the genomes of milu, cattle, human and sheep.

Table S1: 17-mer depth distribution.

Table S2: Summary of BUSCO analysis of matches to the 4,104 mammalian BUSCOs.

Table S3: Summary of breakpoints between milu and goat.

Table S4: TE contents in the assembled milu genome.

Table S5: General statistics of predicted protein-coding genes.

Table S6: Summary statistics of gene function annotation.

Table S7: The distribution of single nucleotide variant (SNV) in the milu genome.

Table S8: Summary of ncRNA annotation.

Figure 1

Click here to download Figure Figure 1.jpg

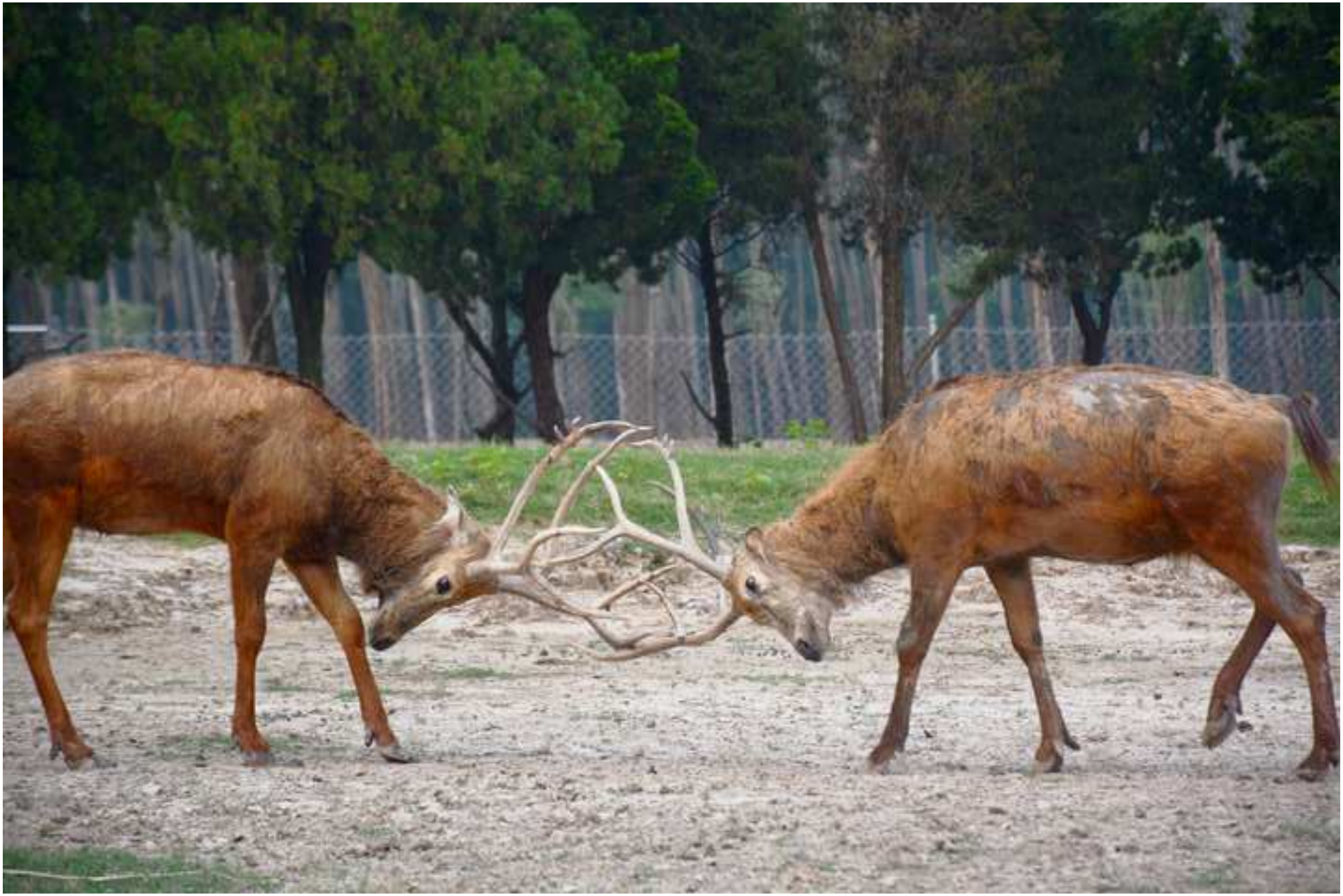

Figure 2 Click here to download Figure Figure 2.jpg

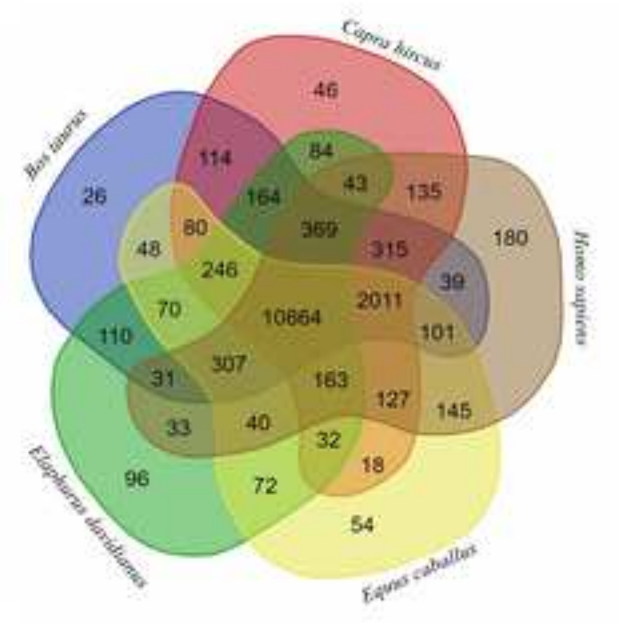

(a)

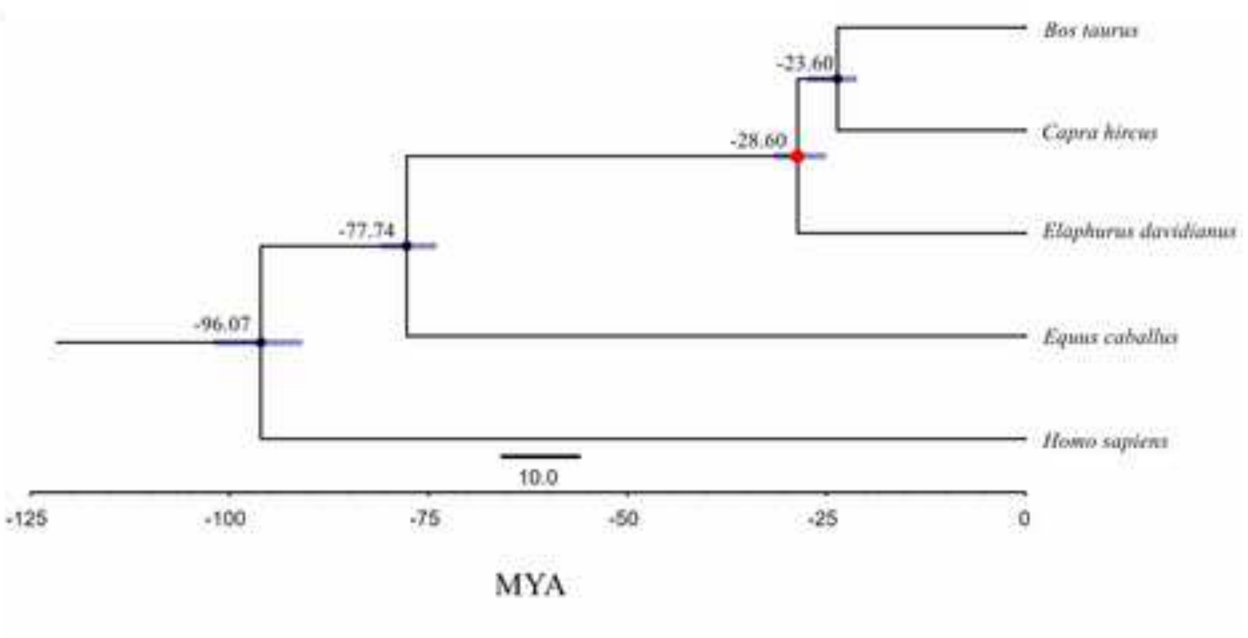

(b)

Click here to access/download
Supplementary Material
Suplementary files.pdf

Personal Cover Click here to download Personal Cover Coverletter.docx

GigaScience

Xi’an, 29 June 2017

Dear Laurie,

We submit two manuscripts entitled “Draft genome of the Marco Polo Sheep (*Ovis ammon polii*)” and “Draft genome of the milu (*Elaphurus davidianus*)” through regular submission system, and one manuscript entitled “Draft genome of the Reindeer (*Rangifer tarandus*)” through bioRxiv transfer, for your consideration if they can be published by *GigaScience* as Data Note articles.

If you have more questions, please do not hesitate to contact me.

Sincerely yours,

Qiang Qiu
Center for Ecological and Environmental Sciences,
Northwestern Polytechnical University,
Xi’an, China.

Reviewers whom we would like to exclude due to conflict of interest:

Draft genome of the Marco Polo Sheep (*Ovis ammon polii*)

1. Jianquan Liu, PhD, Professor
liujq@lzu.edu.cn / liujq@nwipb.cas.cn
School of Life Sciences
Sichuan University / Lanzhou University, China

Draft genome of the Reindeer (*Rangifer tarandus*):

1. Glenn Yannic, PhD, Associate Professor
glenn.yannic@univ-smb.fr / glenn.yannic@gmail.com
Laboratoire d’Ecologie Alpine (LECA) - UMR CNRS 5553
Université Savoie Mont Blanc

2. Steeve Côté, PhD, Professor
Steeve.Cote@bio.ulaval.ca
Department of biology, Université Laval
Sainte-Foy, Québec Canada

3. Louis Bernatchezclose, PhD, Professor

louis.bernatchez@bio.ulaval.ca
Department of biology, Université Laval

4. Knut Røed , PhD, Professor
knut.roed@nmbu.no
Department Of Basic Sciences and Aquatic Medicine
Norwgian University of Life Sciences

5. Juha Kantanen, PhD, Professor
juha.kantanen@luke.fi
Natural Resources Institute Finland (Luke)
Helsinki, Uusima, Finland

6. Jianquan Liu, PhD, Professor
liujq@lzu.edu.cn / liujq@nwipb.cas.cn
School of Life Sciences
Sichuan University / Lanzhou University, China

Draft genome of the milu (*Elaphurus davidianus*)

1. Lifeng Zhu, Phd, Professor
zhulf@ioz.ac.cn
College of Life Sicence, Nanjing Normal University, Nanjing, China

2. Guoqing Lu, Professor
Email: glu3@unomaha.edu
Department of Biology and School of Interdisciplinary Informatics
University of Nebraska at Omaha, Omaha, USA.

3. Jianquan Liu, PhD, Professor
liujq@lzu.edu.cn / liujq@nwipb.cas.cn
School of Life Sciences
Sichuan University / Lanzhou University, China
