# Supplementary material for: Draft genome of the milu (Elaphurus davidianus)
Source: Gigascience. 2017 Dec 18;7(2):gix130. doi: 10.1093/gigascience/gix130 (PMC5824821; doi:10.1093/gigascience/gix130)
Supplement: GIGA-D-17-00161_Revision_1.pdf [file gix130_giga-d-17-00161_revision_1.pdf]

GigaScience

Draft genome of the milu (Elaphurus davidianus)

--Manuscript Draft--

**Manuscript Number:**	GIGA-D-17-00161R1
**Full Title:**	Draft genome of the milu (Elaphurus davidianus)
**Article Type:**	Data Note
**Funding Information:**	Talents Team Construction Fund of Northwestern Polytechnical University (NWPU) — Dr. Wen Wang, Dr. Qiang Qiu
**Abstract:**	Abstract<br>Background: Milu, also known as Père David's deer (Elaphurus davidianus), had been widely distributed in East Asia but experienced a severe bottleneck (only 18 survived by the end of 19th century), and the current 4500 individual population was propagated from only 11 individuals reared by the 11th British Duke of Bedford. This species is known for its distinguishable appearance, the driving force behind which is still a mystery. The draft genome reported in this study will provide valuable resources to investigate deeper into its evolutionary history and population dynamics of severely bottlenecked species.<br>Findings: In total, we generated 321.86 gigabases (Gb) of raw DNA sequence from whole-genome sequencing of the male milu deer using an Illumina HiSeq 2000 platform. Assembly gave a final genome with scaffold N50 of 3.03 megabases (Mb), and total length of 2.52 Gb. Moreover, we identified 20,125 protein-coding genes and 988.1 Mb of repetitive sequences. In addition, homology-based searches detected 280 rRNA, 1,335 miRNA, 1,441 snRNA and 893 tRNA sequences in the genome of E. davidianus. The divergence time between E. davidianus and the ancestors Bos taurus and Capra hircus, was estimated to be about 28.20 million years ago (Mya). We identified 167 species-specific genes and 293 expanded gene families in the milu lineage.<br>Conclusions: We report the first reference genome of milu. The genome will provide a valuable resource for studying the species' demographic history and the population genetic dynamics for severely bottlenecked species.<br><br>Keywords: Elaphurus davidianus, Reference genome, Evolution
**Corresponding Author:**	Qiang Qiu, Ph.D.<br><br>CHINA
**Corresponding Author Secondary Information:**	
**Corresponding Author's Institution:**	
**Corresponding Author's Secondary Institution:**	
**First Author:**	Chenzhou Zhang
**First Author Secondary Information:**	
**Order of Authors:**	Chenzhou Zhang<br>Lei Chen, Ph.D.<br>Yang Zhou<br>Kun Wang, Ph.D.<br>Leona G. Chemnick, Ph.D.<br>Oliver A. Ryder, Ph.D.<br>Wen Wang, Ph.D.<br>Guojie Zhang, Ph.D.

Powered by Editorial Manager® and ProduXion Manager® from Aries Systems Corporation

	Qiang Qiu, Ph.D.
**Order of Authors Secondary Information:**	
**Response to Reviewers:**	Editor's comments:<br>In particular, the reviewers raise some questions regarding the quality of the assembly and the annotation, and whether the validation steps follow best practice (for example, with regard to using the same short-read data in the assembly process as a validation dataset). Please carefully consider all points of the reviewers and revise your manuscript (and your analyses, e.g. annotations, where necessary) to address these concerns. We agree that the dataset will be useful for the community, but the methods, including potential shortcomings and limitations, need to be clearly explained and discussed in the manuscript.<br>Reply: We appreciate the editor's positive comments on the value of the genomic resources of milu in this work. In our revised manuscript, all the reviewers' suggestions have been fully considered. Firstly, we re-estimated the milu genome size using several more models, as described below. Secondly, we re-annotated the genes using the most recent Ensembl releases (version 89) for human, cattle and pig proteins. We have also listed pseudogenes, as requested by Reviewer 2. Thirdly, we re-annotated the gene functions and gene families, and re-constructed phylogenetic relationships, using the new gene annotations. Finally, we added discussion of potential shortcomings and limitations of our results in the revised manuscript.<br><br>I note that you indicate several corresponding authors. OUP has a policy of only taking one - most responsive - author as corresponding author. The definition of corresponding authorship is one of responsiveness rather than seniority. The corresponding author is the one individual who takes primary responsibility for communication with the journal during the manuscript submission, peer review, and publication process. Please refer to the information below on our homepage and please decide based on these guidelines who should be corresponding author.<br>You indicate several "co-first" authors with equal contributions. Please explain in more detail in how far the contributions of all three first authors are exactly equal. See also CASRAI guidelines to define authorship roles:<br>Reply: We have carefully read the EDITORIAL POLICIES & REPORTING STANDARDS in the author guidelines provided on GigaScience's website. Our authorship was based on the criteria listed in the guidelines and all the authors meet the requirements. Following the policy of GigaScience that there could be at most two correspondent authors, with the approval of all the authors, now we have only listed two correspondent authors. Nowadays most genome projects are done by collaboration of several groups, both correspondent authors contributed to the design of the project and organization of the manuscript. Now Dr, Qiang Qiu is the primary correspondent author to communicate with the journal during the manuscript submission, peer review and publication process. We have modified the description of each author's contributions, as follows, and showed that the first three were approximately equal.<br>"Q.Q. W.W. and G.Z. conceived the study. C.Z. and L.C. designed the scientific objectives. LGC and OAR evaluated and provided samples from San Diego Zoo Global. Y.Z. collected the samples, extracted the genomic DNA and constructed the DNA libraries. C.Z. and Y.Z. estimated the milu genome size and constructed the genome assembly. C.Z., L.C. and Y.Z. carried out the quality assessment, repeat annotation and gene annotation. C.Z. and K.W. were responsible for finding species-specific genes and phylogenetic relationship construction. L.C. and C.Z. uploaded the raw reads data, genome assembly and annotation to NCBI and GigaScience GigaDB databases. C.Z., Q.Q. and W.W. wrote the manuscript. Q.Q., W.W. and G.Z. supervised all aspects of the work to ensure the accuracy and integrity of the research and data. OAR contributed to editing the final manuscript. All authors read and approved the final manuscript."<br><br>Reviewer reports:<br>Reviewer #1: Summary: In this manuscript, Zhang et al. present a new genome assembly for the Milu deer (Elaphurus davidianus) consisting entirely of short read Illumina data. They generated the reference assembly using sequence data libraries of different insert sizes input into the SOAPdenovo assembly pipeline. The reported assembly quality metrics appear to be good for an assembly of this type; however, there are some details missing from the authors' report that need to be clarified.

Powered by Editorial Manager® and ProduXion Manager® from Aries Systems Corporation

	Reply: We are grateful for the reviewer’s helpful suggestion, and have tried to address all the major and minor points raised.<br><br>Major points:<br>1. Line 44: For non-speakers, can you provide a translation of "Sibuxiang?" The context of the sentence implies that this name has some significance related to the animal's phenotype.<br>Reply: “Sibuxiang” is a colloquial name for milu that has been widely used in China, and could be translated as “the four unlikes”, because it has the hooves of a cow but is not a cow, the head of a horse but is not a horse, antlers of a deer but is not a deer, and the tail of a donkey but is not a donkey. In ancient times, most Chinese people regarded the milu as a mythical creature due to its rarity and strange appearance. We have briefly provided this information in the revised manuscript, as follows:<br>“…Milu also has a colloquial name in China, Sibuxiang, which could be translated as "the four unlikes", because it has the hooves of a cow but is not a cow, the head of a horse but is not a horse, antlers of a deer but is not a deer, and the tail of a donkey but is not a donkey...” (p4 lines 44-47).<br><br>2. Line 64: Were proper animal care and use protocols followed in the extraction of the sample from the reference animal?<br>Reply: We thank the reviewer for pointing out this. We apologized for the omitting of the details on this part. We contacted with our collaborators of San Diego Zoo again and we verified the animal care and use protocols were performed in accordance with the San Diego Zoo Global's Institutional Animal Care and Use Committee policies and the ethical guidelines of Northwestern Polytechnical University and BGI-Shenzhen Laboratory Animal Care and Use Committee. We added an ethics statement and the authorship of our San Diego Zoo collaborators in the revised manuscript (p1 lines 3-4 and 10; p13 lines 252-259).<br><br>3. Line 69: Why were the short-insert library read pairs only sequenced to 49 bp in length? This seems to be very counterintuitive given that these libraries were eventually used to close gaps in SOAPdenovo scaffolds.<br>Reply: We thank the reviewer for spotting this mistake: the read lengths for the short-insert and long-insert libraries were 100 bp and 49 bp, respectively. We have corrected this error (p5 line73).<br><br>4. Line 84: The provided equation terms (e.g. "k-mer_number") do not match the definitions in the following sentence (e.g. "N"). Please reformat the equation to display consistent terms.<br>Reply: We thank the reviewer for pointing out this. The “N” and “K_depth” refer to “k-mer_number” and “k-mer_depth”, respectively, in the manuscript, which we have reworded accordingly (p6 lines 88-89).<br><br>5. Line 84: How does this genome size estimate compare against estimates made with more sophisticated models? For example, the Vurture et al. 2017, GenomeScope package uses a far more sophisticated, kmer-based, estimation model.<br>Reply: We appreciate this important comment and agree that more sophisticated models could potentially provide more accurate indications of genome size. However, the estimate obtained with GenomeScope was 2.0 Gb, which is not reasonable. We also utilized the GCE software package, and obtained a genome size of 3.00 GB for milu, which is very close to our previous estimation. Moreover, our estimated genome size is within the C value interval (2.22 to 3.44) of Cervidae reported in the ANIMAL GENOME SIZE DATABASE (http://www.genomesize.com/). Thus, the GenomeScope model did not work in this case, for unknown reasons. We have added a new Table displaying this finding in the revised manuscript (Table S2; p6 lines 89-94).<br><br>6. Line 93: I don't understand why or how scaffolds generated with GapCloser were chosen to be split into new contigs. I believe - from the context of this paragraph - that GapCloser was used to fill SOAPdenovo scaffold gaps, but that unfilled gaps were broken into separate contigs. The split contigs and scaffolds were then subjected to another round of scaffolding using SSPACE. Is this correct? If so, the paragraph needs to be reworded to best reflect the authors' exact protocols.<br>Reply: The reviewer’s understanding is correct and we apologize for the unclear description in this paragraph, which we have amended as follows:

Powered by Editorial Manager® and ProduXion Manager® from Aries Systems Corporation

“Then we used GapCloser (version 1.12) [10] to fill the gaps of initial scaffolds using short-insert sizes PE reads (170, 500 and 800 bp). The initial scaffolds were then divided into scaff-tigs by the unfilled gaps. The divided scaff-tigs were connected to final scaffolds using SSPACE (version 3.0) [14] with the following parameters: -x 0, -z 200, -g 2, -k 2, -n 10. These final scaffolds’ gaps were also closed by GapCloser” (p6 lines 99-104).

7. Line 102: Was this validation dataset separate from the short reads used to construct the reference assembly itself, or were these reads used to generate the reference assembly? Using sequence data from libraries that were not included in the preparation of the reference assembly would give an unbiased estimate of assembly quality.
Reply: The reviewer raises a good point, and we agree that using sequence data from libraries that were not included in preparation of the reference assembly could give an unbiased estimation. Since all the previous data had been used in the reference assembly and there was also no more tissue from the de novo sequencing individual, we therefore re-sequenced another male milu deer and mapped the clean reads on this genome assembly using SOAPaligner with the same parameters. The results demonstrate that <2% of bases had a sequencing depth less than 10. This alignment also confirmed the accuracy at base level of this reference assembly. We have re-written the relevant sentence in the revised manuscript to make it clear for reader. (Fig. S3 and p7 lines 112-116).

8. Line 111: Which version of the goat genome was used here? The reference genome for this species has changed recently, so a specific accession ID is needed. Please provide literature references for each reference assembly used, if possible.
Reply: We thank the reviewer for this important point. We used the recently published version of the goat genome, ARS1. We have added the accession ID for this goat genome assembly and cited relevant literature for each reference assembly in our revised manuscript (p7 lines 122-124).

9. Line 112: What was the percent identity cutoff for MUMs in the NUCmer alignments used to determine synteny? A matching synteny block with less than 50% ID is meaningless in terms of sequence-to-sequence comparisons even at a large scale.
Reply: Yes, as mentioned in the revised manuscript, the percent identity cutoff for MUMs was 50% (p7 lines 126-127).

10. Line 117: How were structural variants assessed? By strict alignment metrics between the two assemblies? Were gaps considered when making structural variant calls? More details are needed here. The inclusion of structural variant size metrics (ie. average size and standard deviations) in Table S2 is necessary to assess the structural fidelity of this assembly against the goat reference.
Reply: We thank the reviewer for raising this point. The aim of this comparison was to estimate the quality of our genome assembly rather than find the variation events. As it was not easy for us to classify each structural variation (SV) event between a chromosome-level and a scaffold-level genome sequences from synteny alignment, we had chosen to scan the edge of every SV event (breakpoint) instead of a complete SV event. We then summarized the densities of these breakpoints, which were comparable to findings in another study (Kun Wang, 2016). To clarify this, we have reworded the description in the revised manuscript (p7-8 lines 128-133).

Reviewer #2: This data note describes the generation of the draft genome of the Milu. This is an interesting species of deer that has a unique phenotype and experienced a bottleneck as they were almost extinct, but the population has started to recover and has been re-introduced to China. This paper does deliver what it sets out to describe.
Reply: We appreciate the reviewer’s positive comments on this manuscript and we appreciate your suggestions, which have been highly useful for improving our manuscript.

The main issues I have are with the following section:
Gene annotation:
1. The main problem I have is that for the homology based predictions the Ensembl gene annotation used is very old. Human e60 is over 7 years old and doesn't contain the finished first pass manual annotation. I would expect the predicted number of

	genes to be falsely high. The analyses should be re-run for the latest version of Ensembl.<br>Reply: We thank the reviewer for raising this important issue. We re-ran the homology predictions using the latest Ensembl version (release 89), as suggested. The homology predictions results were integrated with our de novo predictions, and a total of 20,125 genes were obtained. We also applied BUSCO to estimate the annotation quality of the new version, which indicated 93.7% of completeness. The tables, figures and evolutionary analysis have been updated accordingly.<br><br>2. There was also no mention of pseudogenes. I am assuming that the "frame-shifted and prematurely terminated genes" that were removed from the gene set could well be pseudogenes. It would have been nice to have an idea of numbers of these, as these could be interesting data?<br>Reply: The reviewer makes a good point. In our revised manuscript, we regard the frame-shifted and prematurely terminated genes as pseudogenes. Candidate pseudogenes were printed out together with candidate true genes when we ran GeneWise alignment, and were filtered out if they were overlapped with the regions of the final true gene set. We identified a total of 2,803 pseudogenes (1,358 frame-shifted and 2,801 prematurely terminated genes). We have added a new table showing these results in the revised manuscript (Table S7 and S8; p9 lines 166-168).<br><br>3. Putative noncoding RNAs are discussed, but these are in fact short ncRNAs which need to be added as a definition as noncoding RNA is too big a category and doesn't describe the work that was undertaken here. lncRNAs were not addressed at all, despite their rarely being conserved between species and the homology-based approach would not have worked, some mention should have been made of these to show that the full repertoire of RNAs has been considered.<br>Reply: We agree with the referee that lncRNAs are rarely conserved between species, and a homology-based approach rarely detects them. The putative noncoding RNAs discussed in this paper were short ncRNAs, as explicitly stated now in the revised manuscript (p10 lines 180 and 184).<br><br>4. Nomenclature has not been mentioned at all. Perhaps some known genes could have been used to add context to table S6? Has the naming convention for human been followed? I'd like to know more about genes and gene families in general that have been found. Figure 2a needs more defining. What are the gene families that are specific to Milu? I'd like to know.<br>Reply: We thank the reviewer for raising this important issue. The nomenclature followed the naming convention for humans and we have added the name of each gene in a gff file. We reconstructed phylogenetic relationships using the new gene set and re-analyzed species-specific genes. We found 69 milu species-specific gene families corresponding to 167 milu species-specific genes, which were enriched in 4 GO categories related to hormone activity, structural constituent of ribosome and etc. What's more, we also added the analysis of the milu expanded gene families, which were enriched in 34 GO categories, and their functions were mainly associated with motor activity, ATPase activity, calcium ion binding and etc. Three new tables were added to show these results (Table S12-S14; p10-11 lines 192-198; p18 lines 396-399).<br><br>Minor points:<br>Library construction:<br>5. ……..male milu tissue…….: Which tissue?<br>Reply: We have added the following details to the revised manuscript to clarify this: "Genomic DNA was extracted from a male milu bred at the San Diego Zoo Safari Park, Escondido, California, USA utilizing heart tissue collected at necropsy, (NCBI Taxonomy ID, 43332)" (p5 lines 67-68).<br><br>6. There are several typos and sections that would benefit from some re-writing (detailed below).<br>Reply: We have rewritten these sentences.<br><br>Background:<br>7. …….due to intense human an natural pressures………….: such as? what does this mean?

Powered by Editorial Manager® and ProduXion Manager® from Aries Systems Corporation

	Reply: We have rewritten this sentence (p4 lines47-48 "Due to intense human and natural pressures, such as excessive hunting by humans and habitat degradation, milu became extinct.")<br><br>8. This sentence needs re-writing as its too long and a bit muddled.<br>Reply: Done.<br><br>9. …..Special phenotype……: I think specific phenotype would be better.<br>Reply: The "Special phenotype" here mainly referred to its unusual appearance. We have modified the text as follows:<br>"…little is still known about the genetic architecture underlying its unique phenotypic features, such us its unusual appearance, and the population dynamics during the milu's recovery from the severe bottleneck..." (p4 lines 59-61)<br><br>10. Table S6: This doesn't make sense. What is it showing me?<br>Reply: We apologize for omitting the total number and the percentage of annotated genes in Table S6. We have added total values to this table. We also re-ran the gene function annotation using the new gene set, and revised the statistics presented in the table accordingly (Table S9).<br><br>11. Table S8: Need to add total for number of miRNAs.<br>Reply: Changed as suggested (Table S11).
**Additional Information:**	
**Question**	**Response**
Are you submitting this manuscript to a special series or article collection?	No
**Experimental design and statistics**<br><br>Full details of the experimental design and statistical methods used should be given in the Methods section, as detailed in our Minimum Standards Reporting Checklist. Information essential to interpreting the data presented should be made available in the figure legends.<br><br>Have you included all the information requested in your manuscript?	Yes
**Resources**<br><br>A description of all resources used, including antibodies, cell lines, animals and software tools, with enough information to allow them to be uniquely identified, should be included in the Methods section. Authors are strongly encouraged to cite Research Resource Identifiers (RRIDs) for antibodies, model organisms and tools, where possible.<br><br>Have you included the information requested as detailed in our Minimum Standards Reporting Checklist?	Yes

**Availability of data and materials**<br><br>All datasets and code on which the conclusions of the paper rely must be either included in your submission or deposited in publicly available repositories (where available and ethically appropriate), referencing such data using a unique identifier in the references and in the "Availability of Data and Materials" section of your manuscript.<br><br>Have you have met the above requirement as detailed in our Minimum Standards Reporting Checklist?	Yes

Manuscript Click here to download Manuscript Milu-revised MS.docx

Draft genome of the milu (*Elaphurus davidianus)*

Chenzhou Zhang[1, †], Lei Chen[1, †], Yang Zhou[2, 3, †], Kun Wang[1], Leona G. Chemnick[4], Oliver A. Ryder[4], Wen Wang[1], Guojie Zhang[2, 3, 5, *], Qiang Qiu[1, *]

[1] Center for Ecological and Environmental Sciences, Key Laboratory for Space Bioscience & Biotechnology, Northwestern Polytechnical University, Xi'an, 710072, China.

[2] China National Genebank, BGI-Shenzhen, Shenzhen 518083, China

[3] BGI-Shenzhen, Shenzhen 518083, China

[4] San Diego Zoo Institute for Conservation Research, Escondido, CA 92027, USA

[5] Centre for Social Evolution, Department of Biology, Universitetsparken 15, University of Copenhagen, Copenhagen 2100, Denmark

[*]Correspondence: qiuqiang@lzu.edu.cn (QQ), zhanggj@genomics.cn (GZ)

[†]These authors contributed equally to this work.

Abstract

Background: Milu, also known as Père David's deer (*Elaphurus davidianus*), had been widely distributed in East Asia but experienced a severe bottleneck (only 18 survived by the end of 19th century), and the current 4500 individual population was propagated from only 11 individuals reared by the 11th British Duke of Bedford. This species is known for its distinguishable appearance, the driving force behind which is still a mystery. The draft genome reported in this study will provide valuable resources to investigate deeper into its evolutionary history and population dynamics of severely bottlenecked species.

Findings: In total, we generated 321.86 gigabases (Gb) of raw DNA sequence from whole-genome sequencing of the male milu deer using an Illumina HiSeq 2000 platform. Assembly gave a final genome with scaffold N50 of 3.03 megabases (Mb), and total length of 2.52 Gb. Moreover, we identified 20,125 protein-coding genes and 988.1 Mb of repetitive sequences. In addition, homology-based searches detected 280 rRNA, 1,335 miRNA, 1,441 snRNA and 893 tRNA sequences in the genome of *E. davidianus.* The divergence time between *E. davidianus* and the ancestors *Bos taurus* and *Capra hircus*, was estimated to be about 28.20 million years ago (Mya). We identified 167 species-specific genes and 293 expanded gene families in the milu lineage.

Conclusions: We report the first reference genome of milu. The genome will provide a valuable resource for studying the species' demographic history and the population genetic dynamics for severely bottlenecked species.

Keywords: *Elaphurus davidianus*, Reference genome, Evolution

Data description

Background

Père David's deer (*Elaphurus davidianus*), named after its western finder (Father *Armand David*) and called "milu" in China, was an endemic species that was once widely distributed in East Asia [1, 2]. Milu also has a colloquial name in China, *Sibuxiang*, which could be translated as "the four unlikes", because it has the hooves of a cow but is not a cow, the head of a horse but is not a horse, antlers of a deer but is not a deer, and the tail of a donkey but is not a donkey (**Fig. 1**). Due to intense human and natural pressures, such as excessive hunting by humans and habitat degradation, milu became extinct in China by the end of the 19th century and only 18 individuals survived in several European zoos at that time. The 18 survived individuals were collected by the 11th British Duke of Bedford and kept at Woburn Abbey (UK) and only 11 milu took part in subsequent reproduction [3]. After this severe bottle-neck, the milu population started to recover. In the 1980s, dozens were reintroduced into China, and there were over 1,500 in China and more than 3,000 worldwide by 2004 [4]. As a surviving cervid that luckily escaped extinction, milu has highly specialized traits compared with other cervid species such as relatively long tail and unique branched antlers. Due to these specific characteristics, scientists once identified it as the root of the subfamily Cervinae, but subsequent molecular analysis indicated that milu is closer to genus *Cervus* [5-9]. However, little is still known about the genetic architecture underlying its unique phenotypic features, such us its unusual appearance, and the population dynamics during the milu's recovery from the severe bottleneck. A good quality reference genome of milu will provide an opportunity to investigate

effects of the severe recent bottleneck and the molecular mechanism of special phenotypic evolution.

Library construction, and filtering

Genomic DNA was extracted from a male milu bred at the San Diego Zoo Safari Park, Escondido, California, USA utilizing heart tissue collected at necropsy, (NCBI Taxonomy ID, 43332). The extracted DNA was used to construct short-insert libraries (170, 500 and 800 base pair, bp) and subsequently long-insert libraries (2, 5, 10 and 20 kilo base, kb). After that, a HiSeq 2000 platform (Illumina; CA, USA) was used to sequence paired end reads of each library based on a whole genome shotgun sequencing strategy, generating 100 bp and 49 bp reads from the short-insert and long-insert libraries, respectively. In total, 321.86 Gb raw data was obtained.

Raw reads were filtered according to the following criteria: (1) reads had > 5% uncalled ("N") bases or polyA structure; (2) reads had 30 and 60 bases with quality scores ≤ 7 for reads generated from the long-insert and short-library sequences, respectively; (3) more than 10 bp aligned to the adapter sequence; (4) read1 and read2 (of a short-insert PE read) overlapped by ≥ 10bp, allowing 10% mismatch; (5) duplicated PCR sequences. The low quality bases at heads or tails of reads were also trimmed. Then, a 249.01 Gb clean data was obtained, representing about 100-fold genome coverage. After that, the short-insert library reads were corrected using SOAPec [10], a k-mer-based error correction package.

Estimation of milu genome size

The milu genome size (G) was estimated by K-mer frequency distribution analysis of the short-insert libraries, with a 1-bp slide and k set at 17, using the formula G = k-mer_number/k-mer_depth [10]. Here, 'k-mer_number' is 1,592,668,741 and the expected 'k-mer_depth' is 25 (**Fig. S1**). The estimated milu genome size, with these parameters, is about 3.04 Gb (**Table S1**). For comparison, we also applied GCE software to estimate the milu genome size, and obtained an estimate of 3.00 Gb [11]. Both of our estimated genome sizes are within the C value interval (2.22 to 3.44) of Cervidae reported in the ANIMAL GENOME SIZE DATABASE (http://www.genomesize.com/), indicating our estimations is credible [12] (**Table S2**).

Genome assembly

SOAPdenovo software (version 2.04) [13] with parameters (pregraph-K 79; contig -M 1; scaff –L 200 -b 1.5 -p 40) were applied to construct the original contigs and initial scaffolds using corrected reads for the assembly of milu genome. Then we used GapCloser (version 1.12) [10] to fill the gaps of initial scaffolds using short-insert sizes PE reads (170, 500 and 800 bp). The initial scaffolds were then divided into scaff-tigs by the unfilled gaps. The divided scaff-tigs were connected to final scaffolds using SSPACE (version 3.0) [14] with the following parameters: -x 0, -z 200, -g 2, -k 2, -n 10. These final scaffolds' gaps were also closed by GapCloser. The total length of our final milu genome assembly is 2.52 Gb accounting for 83.05% of the estimated genome size. The final contig N50 and scaffold N50 (>2 kb) sizes are 32.71 kb and

3.03 Mb, respectively (**Table 1**).

Quality assessment

To evaluate the quality of the milu genome assembly, the filtered reads (≥ 49 bp) were aligned to the assembled genome sequences using SOAPaligner (version 2.20) [13] allowing 3 mismatches. We also re-sequenced another male milu deer (unpublished data). The clean reads of this re-sequenced deer was also aligned to the assembled genome by SOAPaligner with the same parameters. Both alignments showed high coverage of each genome base, confirming accuracy at the base level (**Fig. S2 and Fig. S3**). In addition, analysis with BUSCO (benchmarking universal single-copy orthologs, version 2.0) [15] showed that the assembly included complete matches for 3820 of 4104 mammalian BUSCOs (indicating 93.00% completeness) (**Table S3**). FRC (Feature-response curves, version 1.3.0) [16] was then used to evaluate the trade-off between its contiguity and correctness. FRC curves generated by the software showed that our milu genome assembly has similar correctness to the published genomes of another three ruminants: domestic goat (*Capra hircus*, ARS1, GenBank ID: GCF_001704415.1) [17], sheep (*Ovis aries*, Oar_v3.1) and cattle (*Bos taurus* UMD3.1) (**Fig. S4**) [18, 19]. Subsequently, synteny analysis was applied to identify differences between the assembled genome and the domestic goat (*Capra hircus*) genome using MUMmer (version 3.23) [20], with a 50% identity cutoff for MUMs in the NUCmer alignments used to determine synteny **(Fig. S5**). 99.35% of the two genome sequences could be 1:1 aligned. In addition, we compared the milu

and goat genomes using LAST (version 3) [21] to find the breakpoints (edges of structural variation). The overall density of different types of breakpoints was about 54.76 per Mb, comparable to densities reported in another study (**Table S4**) [22], and the average nuclear distance (percentage of different base pairs in the syntenic regions) was 6.56% (**Fig. S6**). The results indicated that the milu genome assembly has good completeness and continuity.

Repeat annotation

To annotate repeats, we first searched the milu genome for tandem repeats using Tandem Repeats Finder (version 4.04) [23] with the following settings: Match = 2, Mismatch = 7, Delta = 7, PM = 80, PI = 10, Minscore = 50. Then, RepeatMasker (version 3.3.0) and RepeatProteinMask (version 3.3.0, a package within RepeatMasker) [24] were used to find known transposable element (TE) repeats in the Repbase TE library (version 16.01) [25]. In addition, RepeatModeler (version 1.0.5) and LTR_FINDER (version 1.0.5) [26] were used to construct a *de novo* repeat library and RepeatMasker was employed to find homolog repeats in the genome and to classify the detected repeats. The results indicated that the identified repeat sequences accounted for 41.04% milu genome, and predominated by the long interspersed elements (27.05%) (**Table S5**).

Gene annotation

To annotate structures and functions of putative genes in our milu genome assembly

we used both homology-based and *de novo* predictions. For homology-based predictions, homologous proteins of *Homo sapiens* (Ensembl 89 release), *Bos taurus* and *Sus scrofa* (Ensembl 89 release) were aligned to the repeat-masked milu genome using TblastN (Blastall 2.2.23) with an E-value cutoff 1e-5. Then aligned sequences and corresponding query proteins were filtered and passed to GeneWise (version 2.2.0) [27] for accurate spliced alignments. Gene sequences shorter than 150 bp, and frame-shifted or prematurely terminated genes, were removed. *De novo* predictions were obtained from analysis of the repeat-masked genome using Augustus (version 2.5.5) [28] and Genscan (version 1.0) [29], with parameters generated from training with *Homo sapiens* genes. The filter processes applied in the homology-based prediction procedure were also applied in the *de novo* predictions. Next, the obtained results were integrated using GLEAN (version 1.0.1) [30], then genes with few exons (≤ 3), which could not be aligned well in SwissProt or TrEMBL were filtered to produce a final consensus gene set containing 20,125 genes. The number of genes, gene length distribution, exon number per gene and intron length distribution were similar to those of other mammals **(Fig. S7 and Table S6).** We also identified a total of 2,803 pseudogenes from GeneWise alignment, of which 2,801 had prematurely terminated mutations, and 1,358 had frame-shifted mutations **(Table S7 and S8)** [30].

Then, the KEGG, SwissProt and TrEMBL databases were searched for best matches to the final gene set using BLASTP (version 2.2.26) with an E-value of 1e-5. Subsequently, InterProScan software (version 5.18-57.0) was applied to map putative encoded protein sequences against entries in the Pfam, PRINTS, ProDom and

SMART databases to identify known motifs and domains. In total, at least one function was allocated to 17,913 (89.31%) of the genes in this manner (**Table S9**). Next, about 27-fold genome coverage reads form short-insert libraries were mapped to the milu genome with BWA (version 0.7.15-r1140) [31] and called variants by SAMtools (version 1.3.1) [32] subsequently. Finally, SnpEff (version 4.10) [33] was applied to identify the distribution of single nucleotide variant (SNV) in the milu genome (**Table S10**).

In addition, putative short noncoding RNAs were identified by BLASTN alignment of human rRNA sequences with milu homologs. We employed Infernal (version 0.81) with Rfam database (release 9.1) to annotate the miRNA and snRNA genes. The tRNAs were annotated using tRNAscan-SE (version 1.3.1) software with default parameters. In total, 3,949 short noncoding RNA sequences were identified in the milu deer genome (**Table S11**).

Species-specific genes and phylogenetic relationship

The detected milu genes were clustered in families by employing OrthoMCL (version 2.0.9) [34] with an E-value cutoff of 1e-5, and a Markov Chain Clustering with default inflation parameter in an all-to-all BLASTP analysis of entries for five species (*Homo sapiens, Equus caballus, Capra hircus, Bos taurus*, and *Elaphurus davidianus*). The result indicated that 69 gene families and 167 genes were specific to milu while 293 gene families that had expanded in milu lineage using CAFÉ (Computational Analysis of gene Family Evolution) [35] (Fig. 2a, Table S12). The milu

species-specific gene families were enriched in 4 GO categories related to hormone activity, structural constituent of ribosome and etc. And the expanded gene families were enriched in 34 GO categories and their functions were mainly associated with motor activity, ATPase activity, calcium ion binding and etc (Table S13 and S14). Subsequently, 7,906 one to one single orthologs were identified from these species and were aligned using PRANK (version 3.8.31) [36]. Next, we extracted 4D-sites (four-fold degenerated sites) to construct a phylogenetic tree by RAxML (version 7.2.8) [37] with GTR+G+I model. Finally, phylogenetic analysis applying PAML MCMCtree (version 4.5) [38], calibrated with published timings of the divergence of the reference species (http://www.timetree.org/), showed that *Elaphurus davidianus*, *Bos taurus* and *Capra hircus* diverged from a common ancestor approximately 28.20 million years ago (**Fig. 2b**).

In summary, we report the first sequencing, assembly, and annotation of the milu genome. The assembled draft genome will provide a valuable resource for studying the species' evolutional history, as well as genetic changes and associated phenomena, such as genetic load and selection pressures that occurred during its severe bottle-neck period or other unknown historical events. It should be noted that this draft assembly was generated by NGS data and there might exist some errors in highly GC-biased or repeated regions. Moreover, this genome assembly should be elevated to chromosomal level in future with Hi-C, optical mapping or genetic mapping technologies.

Supporting data

The raw reads of each sequencing library have been deposited at NCBI with the project ID: PRJNA391565, Sample ID: SAMN07270940 and the Genome Sequence Archive [39] under BIG Data Center [40], Beijing Institute Genomics (BIG), Chinese Academy of Science, Project ID: PRJCA000448, Sample ID: SAMC013083. The assembly and annotation of the milu genome are available in the the *GigaScience* GigaDB database. Supplementary Figures and Tables are provided in Additional file 1.

Abbreviations

Gb: giga base; bp: base pair; kb: kilo base; Mb: mega base; TE: transposable element; BUSCO: benchmarking universal single-copy orthologs; FRC: feature-response curves; SNV: single nucleotide variant;

Acknowledgements

This study was supported by Talents Team Construction Fund of Northwestern Polytechnical University (NWPU) to QQ and WW. We thank Nowbio Biotech lnc,. Kunming, China for the remarkable work on DNA libraries constructions and the assistance during the genome sequencing. This project was initiated under the auspices of the Genome 10K Project.

Authors' contributions

Q.Q. W.W. and G.Z. conceived the study. C.Z. and L.C. designed the scientific objectives. LGC and OAR evaluated and provided samples from San Diego Zoo Global. Y.Z. collected the samples, extracted the genomic DNA and constructed the DNA libraries. C.Z. and Y.Z. estimated the milu genome size and constructed the genome assembly. C.Z., L.C. and Y.Z. carried out the quality assessment, repeat annotation and gene annotation. C.Z. and K.W. were responsible for finding species-specific genes and phylogenetic relationship construction. L.C. and C.Z. uploaded the raw reads data, genome assembly and annotation to NCBI and *GigaScience* GigaDB databases. C.Z., Q.Q. and W.W. wrote the manuscript. Q.Q., W.W. and G.Z. supervised all aspects of the work to ensure the accuracy and integrity of the research and data. OAR contributed to editing the final manuscript. All authors read and approved the final manuscript.

Ethics statement

Animal collection and utility protocols were approved by the Northwestern Polytechnical University and BGI-Shenzhen Laboratory Animal Care and Use Committee, and were in accordance with guidelines from the China Council on Animal Care. Samples provided by San Diego Zoo Global (SDZG) were collected in accordance with SDZG's Institutional Animal Care and Use Committee policies, which meet or exceed U.S. regulatory standards for the humane care and treatment of animals in research.

Competing interests

The authors declare that they have no competing interests

References:

1. Harrison RJ, Hamilton WJ: The reproductive tract and the placenta and membranes of Père David's deer (*Elaphurus davidianus* Milne Edwards). Journal of Anatomy. 1952;86 2:203-225.
2. Cao K. On the time of extinction of the wild Mi-deer in China (in Chinese). ACTA ZOOLOGICA SINICA. 1978;24 3:289-291.
3. JONES F: A contribution to the history and anatomy of Père David's Deer (*Elaphurus davidianus*). Journal of Zoology. 1951;2 121:319-370, doi:10.1111/j.1096-3642.1951.tb00800.x.
4. Ding Y. Chinese milu research (in Chinese). Changchun, China: Jilin Publishing House for the Science and Technology; 2004.
5. Tate ML, Mathias HC, Fennessy PF, Dodds KG, Penty JM, Hill DF: A new gene mapping resource: interspecies hybrids between Pere David's deer (*Elaphurus davidianus*) and red deer (*Cervus elaphus*). Genetics. 1995;139 3:1383-1391.
6. Slate J, Van Stijn TC, Anderson RM, McEwan KM, Maqbool NJ, Mathias HC, Bixley MJ, Stevens DR, Molenaar AJ, Beever JE *et al*: A deer (subfamily Cervinae) genetic linkage map and the evolution of ruminant genomes. Genetics. 2002;160 4:1587-1597.
7. Pitra C, Fickel J, Meijaard E, Groves PC: Evolution and phylogeny of old world deer. Molecular Phylogenetics and Evolution. 2004;33 3:880-895, doi:10.1016/j.ympev.2004.07.013.
8. Maqbool NJ, Tate ML, Dodds KG, Anderson RM, McEwan KM, Mathias HC, McEwan JC, Hall RJ: A QTL study of growth and body shape in the inter-species hybrid of Pere David's deer (*Elaphurus davidianus*) and red deer (*Cervus elaphus*). Animal Genetics. 2007;38 3:270-276, doi:10.1111/j.1365-2052.2007.01597.x.
9. Emerson BC, Tate ML: Genetic analysis of evolutionary relationships among deer (subfamily Cervinae). Journal of Heredity. 1993;84 4:266-273.
10. Li R, Fan W, Tian G, Zhu H, He L, Cai J, Huang Q, Cai Q, Li B, Bai Y *et al*: The sequence and de novo assembly of the giant panda genome. Nature. 2010;463 7279:311-317, doi:10.1038/nature08696.
11. Liu B, Shi Y, Fan W: Estimation of genomic characteristics by analyzing k-mer frequency in de novo Estimation of genomic characteristics by analyzing k-mer frequency in de novo genome projects. arXiv preprint. 2013; arXiv:1308.2012.
12. Gregory, T.R. Animal Genome Size Database. http://www.genomesize.com, 2017.
13. Li R, Zhu H, Ruan J, Qian W, Fang X, Shi Z, Li Y, Li S, Shan G, Kristiansen K *et al*: De novo assembly of human genomes with massively parallel short read sequencing. Genome Research. 2010;20 2:265-272, doi:10.1101/gr.097261.109.
14. Boetzer M, Henkel CV, Jansen HJ, Butler D, Pirovano W: Scaffolding pre-assembled contigs using SSPACE. Bioinformatics. 2011;27 4:578-579,

doi:10.1093/bioinformatics/btq683.
15. Simao FA, Waterhouse RM, Ioannidis P, Kriventseva EV, Zdobnov EM: BUSCO: assessing genome assembly and annotation completeness with single-copy orthologs. Bioinformatics. 2015;31 19:3210-3212, doi:10.1093/bioinformatics/btv351.
16. Vezzi F, Narzisi G, Mishra B: Reevaluating assembly evaluations with feature response curves: GAGE and assemblathons. PLoS One. 2012;7 12:e52210, doi:10.1371/journal.pone.0052210.
17. Bickhart DM, Rosen BD, Koren S, Sayre BL, Hastie AR, Chan S, Lee J, Lam ET, Liachko I, Sullivan ST *et al*: Single-molecule sequencing and chromatin conformation capture enable de novo reference assembly of the domestic goat genome. Nature Genetics. 2017, doi:10.1038/ng.3802.
18. Jiang Y, Xie M, Chen W, Talbot R, Maddox JF, Faraut T, Wu C, Muzny DM, Li Y, Zhang W *et al*: The sheep genome illuminates biology of the rumen and lipid metabolism. Science. 2014;344 6188:1168-1173, doi:10.1126/science.1252806.
19. Elsik CG, Tellam RL, Worley KC, Gibbs RA, Muzny DM, Weinstock GM, Adelson DL, Eichler EE, Elnitski L, Guigo R *et al*: The genome sequence of taurine cattle: a window to ruminant biology and evolution. Science. 2009;324 5926:522-528, doi:10.1126/science.1169588.
20. Delcher AL, Salzberg SL, Phillippy AM: Using MUMmer to identify similar regions in large sequence sets. Curr Protoc Bioinformatics. 2003;Chapter 10:10-13, doi:10.1002/0471250953.bi1003s00.
21. Kielbasa SM, Wan R, Sato K, Horton P, Frith MC: Adaptive seeds tame genomic sequence comparison. Genome Research. 2011;21 3:487-493, doi:10.1101/gr.113985.110.
22. Wang K, Wang L, Lenstra JA, Jian J, Yang Y, Hu Q, Lai D, Qiu Q, Ma T, Du Z *et al*: The genome sequence of the wisent (Bison bonasus). Gigascience. 2017, doi:10.1093/gigascience/gix016.
23. Benson G: Tandem repeats finder: a program to analyze DNA sequences. Nucleic Acids Research. 1999;27 2:573-580.
24. Tarailo-Graovac M, Chen N: Using RepeatMasker to identify repetitive elements in genomic sequences. Curr Protoc Bioinformatics. 2009;Chapter 4:4-10, doi:10.1002/0471250953.bi0410s25.
25. Jurka J, Kapitonov VV, Pavlicek A, Klonowski P, Kohany O, Walichiewicz J: Repbase Update, a database of eukaryotic repetitive elements. Cytogenetic and Genome Research. 2005;110 1-4:462-467, doi:10.1159/000084979.
26. Xu Z, Wang H: LTR_FINDER: an efficient tool for the prediction of full-length LTR retrotransposons. Nucleic Acids Research. 2007;35 Web Server issue:W265-W268, doi:10.1093/nar/gkm286.
27. Birney E, Clamp M, Durbin R: GeneWise and Genomewise. Genome Research. 2004;14 5:988-995, doi:10.1101/gr.1865504.
28. Stanke M, Keller O, Gunduz I, Hayes A, Waack S, Morgenstern B: AUGUSTUS: ab initio prediction of alternative transcripts. Nucleic Acids

Research. 2006;34 Web Server issue:W435-W439, doi:10.1093/nar/gkl200.
29. Burge C, Karlin S: Prediction of complete gene structures in human genomic DNA. Journal of Molecular Biology. 1997;268 1:78-94, doi:10.1006/jmbi.1997.0951.
30. Elsik CG, Mackey AJ, Reese JT, Milshina NV, Roos DS, Weinstock GM: Creating a honey bee consensus gene set. Genome Biology. 2007;8 1:R13, doi:10.1186/gb-2007-8-1-r13.
31. Li H: Aligning sequence reads, clone sequences and assembly contigs with BWA-MEM. arXiv preprint arXiv:13033997. 2013.
32. Li H, Handsaker B, Wysoker A, Fennell T, Ruan J, Homer N, Marth G, Abecasis G, Durbin R: The Sequence Alignment/Map format and SAMtools. Bioinformatics. 2009;25 16:2078-2079, doi:10.1093/bioinformatics/btp352.
33. Cingolani P, Platts A, Wang LL, Coon M, Nguyen T, Wang L, Land SJ, Lu X, Ruden DM: A program for annotating and predicting the effects of single nucleotide polymorphisms, SnpEff: SNPs in the genome of Drosophila melanogaster strain w1118; iso-2; iso-3. Fly (Austin). 2012;6 2:80-92, doi:10.4161/fly.19695.
34. Li L, Stoeckert CJ, Roos DS: OrthoMCL: identification of ortholog groups for eukaryotic genomes. Genome Research. 2003;13 9:2178-2189, doi:10.1101/gr.1224503.
35. De Bie T, Cristianini N, Demuth JP, Hahn MW: CAFE: a computational tool for the study of gene family evolution. Bioinformatics. 2006;22 10:1269-1271, doi:10.1093/bioinformatics/btl097.
36. Loytynoja A, Goldman N: An algorithm for progressive multiple alignment of sequences with insertions. Proc Natl Acad Sci U S A. 2005;102 30:10557-10562, doi:10.1073/pnas.0409137102.
37. Stamatakis A: RAxML version 8: a tool for phylogenetic analysis and post-analysis of large phylogenies. Bioinformatics. 2014;30 9:1312-1313, doi:10.1093/bioinformatics/btu033.
38. Yang Z: PAML 4: phylogenetic analysis by maximum likelihood. Molecular Biology and Evolution. 2007;24 8:1586-1591, doi:10.1093/molbev/msm088.
39. Wang Y, Song F, Zhu J, Zhang S, Yang Y, Chen T, Tang B, Dong L, Ding N, Zhang Q *et al*: GSA: Genome Sequence Archive. Genomics Proteomics Bioinformatics. 2017;15 1:14-18, doi:10.1016/j.gpb.2017.01.001.
40. The BIG Data Center: from deposition to integration to translation. Nucleic Acids Research. 2017;45 D1:D18-D24, doi:10.1093/nar/gkw1060.

Figure legends

Figure 1: Photo of two fighting Père David's deer in Dafeng Milu National Reserves, Jiangsu, China. A red wound was spotted on the body of the deer to the right, and winning such fights generally increases mating chances.

Figure 2. Phylogenetic relationships and genomic comparisons between *Elaphurus davidianus. Equus caballus, Capra hircus, Bos taurus,* and *Homo sapiens.* (a) A Veen diagram of the shared orthologues among *Elaphurus davidianus. Equus caballus, Capra hircus, Bos taurus* and *Homo sapiens.* Each number represents a gene family number and the sum of the numbers located in each color, green, yellow, brown, red and blue areas represented the total numbers of the gene families of milu, horse, human, goat and cattle respectively. (b) Divergence time estimates for the five species generated using MCMCtree and the 4-fold degenerate sites; the dots correspond to calibration points and the divergence times were obtained from http://www.timetree.org/; blue nodal bars indicate 95% confidence intervals.

Table 1: Statistics of the assembled sequence length.

	Contig		Scaffold	
	**Size (bp)**	**Number**	**Size (bp)**	**Number**
**N90**	8,530	77,768	520,987	978
**N80**	14,483	55,968	1,045,447	647
**N70**	20,193	41,646	1,614,103	455
**N60**	26,169	30,975	2,222,401	322
**N50**	32,707	22,564	3,039,716	223
**Longest**	292,964	----	17,945,643	----
**Total Size**	2,460,119,591	----	2,524,831,955	----
**Percent of unknown bases**	----	----	2.56%	
**Total Number (≥100 bp)**	----	189,067	----	46,381
**Total Number (≥2 kb)**	----	118,986	----	4,772

Additional files

Figure S1: K-mer (k=25) distribution in the milu genome.

Figure S2: Sequence depth distribution the assembly data.

Figure S3: Sequence depth distribution of the re-sequencing individual (unpublished data).

Figure S4: Feature-response (FR) curves of four ruminant genome assemblies.

Figure S5: Visualized synteny between the milu and goat genomes.

Figure S6: DNA sequence divergence between milu and goat.

Figure S7: Comparison of gene lengths, intron lengths, exon lengths and exon numbers in milu, cattle, human and sheep genomes.

Table S1: 17-mer depth distribution.

Tbale S2: Summary of the C values of Cervidae and the estimated genome sizes of milu.

Table S3: Summary of BUSCO analysis of matches to the 4,104 mammalian BUSCOs.

Table S4: Summary of breakpoints between milu and goat genomes.

Table S5: TE contents in the assembled milu genome.

Table S6: General statistics of predicted protein-coding genes.

Table S7: Summary of the predicted pseudogenes.

Table S8: List of predicted pseudogenes.

Table S9: Summary statistics of gene function annotation.

Table S10: Distribution of single nucleotide variants (SNV) in the milu genome.

Table S11: Summary of short ncRNA annotation.

Table S12: List of milu species-specific genes.

Table S13: GO enrichment results of milu species-specific genes.

Table S14: GO enrichment results of milu expanded families.

Figure 1 Click here to download Figure Figure 1.jpg

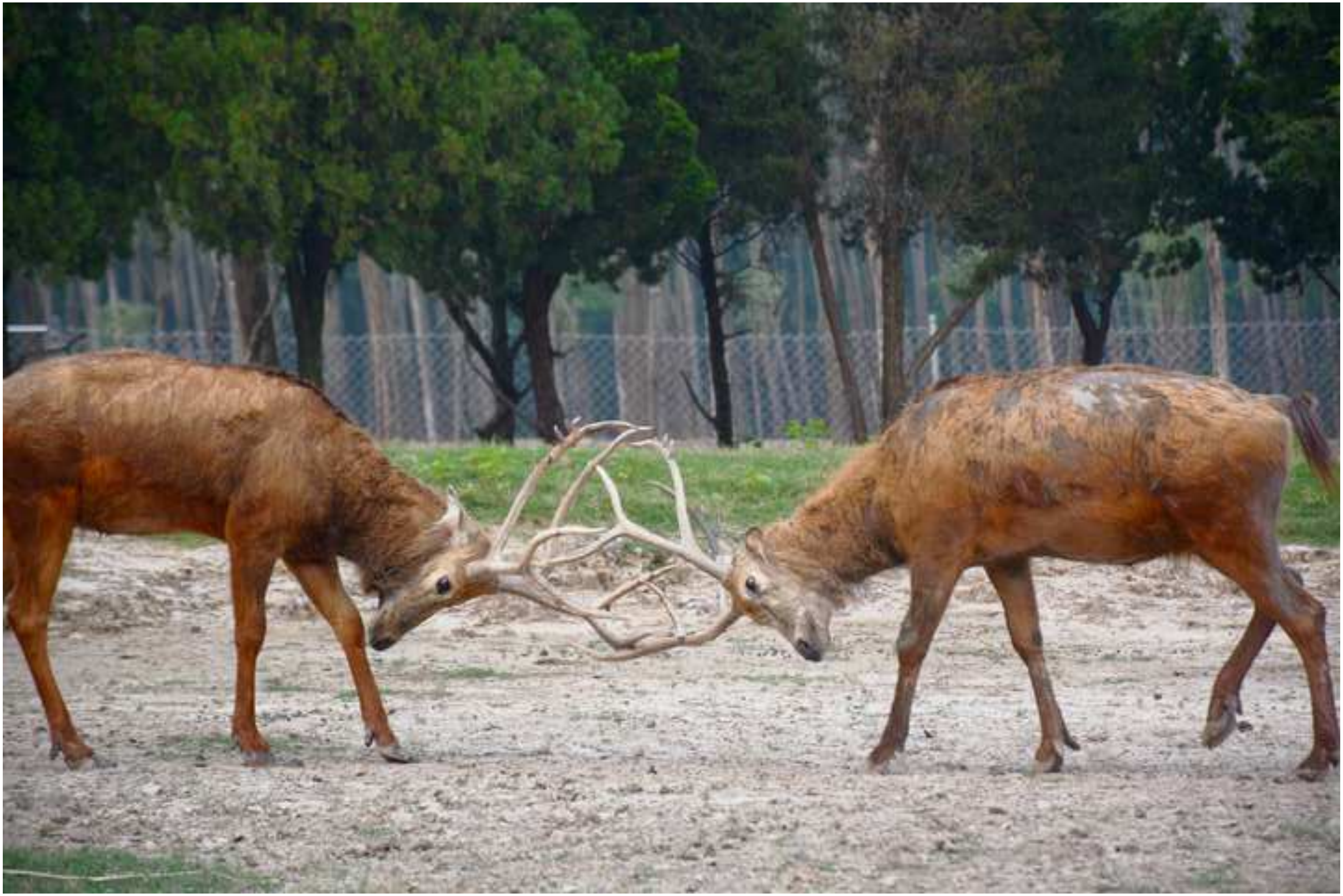

Figure 2

Click here to download Figure Figure 2.jpg

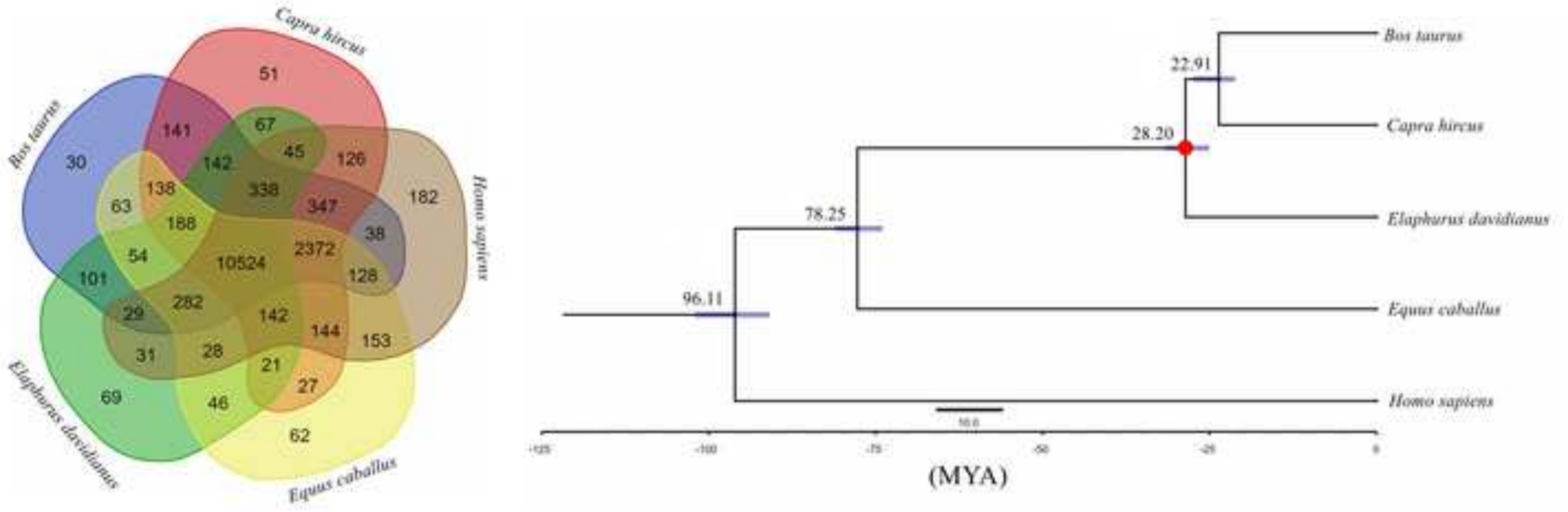

(a) (b)

Click here to access/download
Supplementary Material
Suplementary files.pdf

Click here to access/download
Supplementary Material
Table_S8.xlsx

Click here to access/download
Supplementary Material
Table_S12.xlsx

Click here to access/download
Supplementary Material
Table_S13.xlsx

Click here to access/download
Supplementary Material
Table_S14.xlsx

Click here to access/download
Supplementary Material
Milu-response letter.docx

 Click here to download Personal Cover cover letter.docx

Dear Dr. Hans Zauner,

Thank you very much for returning our manuscript (GIGA-D-17-00161) entitled "Draft genome of the milu (*Elaphurus davidianus*)", together with your helpful comments and those of the referees.

We are grateful for the reviewers' constructive and thoughtful comments, which have helped us to improve our study. We have revised the manuscript according to their suggestions and respond to their comments point-by-point (our responses are indicated in **BOLD** type).

We submit here the revised manuscript and hope that it is now suitable for publication in *GigaScience*. If you have any questions, please do not hesitate to contact the corresponding author at any time.

Thank you again for your time and effort in handling our manuscript.

Qiang Qiu
Center for Ecological and Environmental Sciences,
Northwestern Polytechnical University,
Xi'an, China.
