# Supplementary material for: Draft genome of the milu (Elaphurus davidianus)
Source: Gigascience. 2017 Dec 18;7(2):gix130. doi: 10.1093/gigascience/gix130 (PMC5824821; doi:10.1093/gigascience/gix130)
Supplement: GIGA-D-17-00161_Revision_2.pdf [file gix130_giga-d-17-00161_revision_2.pdf]

GigaScience

Draft genome of the milu (Elaphurus davidianus)

--Manuscript Draft--

**Manuscript Number:**	GIGA-D-17-00161R2
**Full Title:**	Draft genome of the milu (Elaphurus davidianus)
**Article Type:**	Data Note
**Funding Information:**	Talents Team Construction Fund of Northwestern Polytechnical University (NWPU) — Dr. Wen Wang, Dr. Qiang Qiu
**Abstract:**	Abstract<br>Background: Milu, also known as Père David's deer (Elaphurus davidianus), was widely distributed in East Asia but recently experienced a severe bottleneck. Only 18 survived by the end of the 19th century, and the current population of 4500 individuals was propagated from just 11 kept by the 11th British Duke of Bedford. This species is known for its distinguishable appearance, the driving force behind which is still a mystery. To aid efforts to explore these phenomena we constructed a draft genome of the species.<br>Findings: In total, we generated 321.86 gigabases (Gb) of raw DNA sequence from whole-genome sequencing of a male milu deer using an Illumina HiSeq 2000 platform. Assembly yielded a final genome with a scaffold N50 size of 3.03 megabases (Mb), and total length of 2.52 Gb. Moreover, we identified 20,125 protein-coding genes and 988.1 Mb of repetitive sequences. In addition, homology-based searches detected 280 rRNA, 1,335 miRNA, 1,441 snRNA and 893 tRNA sequences in the milu genome. The divergence time between E. davidianus and Bos taurus was estimated to be about 28.20 million years ago (Mya). We identified 167 species-specific genes and 293 expanded gene families in the milu lineage.<br>Conclusions: We report the first reference genome of milu, which will provide a valuable resource for studying the species' demographic history of severely bottlenecked and genetic mechanism of special phenotypic evolution.<br><br>Keywords: Elaphurus davidianus, Reference genome, Evolution
**Corresponding Author:**	Qiang Qiu, Ph.D.<br><br>CHINA
**Corresponding Author Secondary Information:**	
**Corresponding Author's Institution:**	
**Corresponding Author's Secondary Institution:**	
**First Author:**	Chenzhou Zhang
**First Author Secondary Information:**	
**Order of Authors:**	Chenzhou Zhang<br>Lei Chen, Ph.D.<br>Yang Zhou<br>Kun Wang, Ph.D.<br>Leona G. Chemnick, Ph.D.<br>Oliver A. Ryder, Ph.D.<br>Wen Wang, Ph.D.<br>Guojie Zhang, Ph.D.<br>Qiang Qiu, Ph.D.

Powered by Editorial Manager® and ProduXion Manager® from Aries Systems Corporation

Order of Authors Secondary Information:	
Response to Reviewers:	Editor's comments:<br><br>1) I agree with Reviewer 1 that the sequence data of the unrelated milu should also be made publicly available.<br>Reply: Thanks for pointing out that. We have released the short reads obtained from sequencing the unrelated milu deer in the NCBI database, and added the SRA accession numbers in the revised manuscript (p. 10, line 214).<br><br>2) Our data curators will contact you to prepare the supporting dataset that will be posted in our repository, GigaDB.<br>Reply: We have received the curator's mail and provided all the required information.<br><br>3) Please provide all relevant SRA (Short Reads Archive) accession numbers in the "data availability" section of your manuscript<br>Reply: The SRA ID of both individuals have been added in the manuscript (See Table S1 and line 213).<br><br>I noticed that you submitted data to the Genome Sequence Archive at the BIG data center. Please note that this database is not part of the community-accepted INSDC collaboration (http://www.insdc.org/). All sequence data reported in the manuscript must be made available via INSDC databases. Referencing the BIG data center (citations #39/#40) is not necessary, please remove these from the reference list.<br>Reply: The references to the BIG data center have been removed from the reference list and the relevant description in the supporting data paragraph are also deleted.<br><br>4) Please also address all other additional comments of both reviewers in a revised manuscript (e.g. regarding the FRC plots).<br>Reply: In our revised manuscript, all the reviewers' suggestions have been fully considered and addressed.<br><br>5) Please carefully check your revised manuscript for grammatical mistakes and unclear wording (ideally with the help of a native English speaker).<br>Reply: As requested, the revised manuscript has been edited by a professional, native English-speaking editor with a PhD in a relevant discipline, who has addressed both grammatical errors and unclear phrasing.<br><br>Reviewer reports:<br>Reviewer #1: Summary: In this revision, the authors have addressed most of my previous points. There are still minor points within the manuscript that need additional clarity, and I would highly recommend that the authors deposit any additional data they generated in public repositories. After these corrections, I think that the published data will be useful to the Artiodactyla research community.<br><br>Minor points:<br>1. Line 112: I would request that the authors deposit the sequence data from this unrelated Milu deer in a public repository so that other groups can reproduce their results.<br>Reply: We have released the short reads obtained from sequencing the other milu deer in the NCBI database (SRA ID: SRR6287186) and added the SRA ID in the manuscript (p10 line 211).<br><br>2. Line 120: The FRC plot shows a clear distinction between the Milu deer assembly and other published references. What is the reason for this discrepancy? The FRC_align software used to generate the input data for this plot requires alignments of short-insert and, optionally, long-insert libraries against each reference. What short read datasets were used for each reference genome in the calculation?<br>Reply: We thank the reviewer for pointing this out. We expected to obtain a better FRCurve for the milu deer assembly because the reads used for the analysis were generated from the milu sequencing. FRC_align analysis was applied to assess the error in our genome assembly, and it did not indicate that the milu deer assembly is absolutely more accurate than genome assemblies for cattle, goat and sheep. Short-insert and long-insert reads providing ca. 27- and 14-fold coverage, respectively, were randomly selected from the milu sequencing data, and aligned to the milu, cattle,

goat and sheep assemblies to generate bam files for FRC_align analysis.

3. Line 89: It would be useful to include the GenomeScope estimate of genome size here and briefly mention why it is not an accurate measure of genome size in this species.
Reply: We agree. Previously, we obtained the GenomeScope estimate strictly following instructions on the website, with recommended parameters and the kmer length ranging from 17 to 21. The first two steps were carried out by Jellyfish with parameters (jellyfish count -C -m 17 –t 20; jellyfish histo –t 20). The value of the parameter, "–m", was the same as the kmer length of the GenomeScope online run. These two steps were applied to obtain the k-mer spectrum. In the final step, running GenomeScope online, the modeling failed to converge with the kmer length set at 19 to 21. We only obtained convergence with the kmer length set at 17, but the estimated genome sizes were much lower than normal. (See Fig. R1; "len" represents the estimated genome size with kmer length set at 17 and default Max kmer coverage).

We carefully re-read the GenomeScope online guidance and FAQ pages. In one presented case the estimated genome size tended be higher with the parameter Max kmer coverage (default value: 10,000) set at 100,000 or 1,000,000. Thus, we tried setting Max kmer coverage at values ranging from 50,000 to 1,000,000 with the kmer length set at 17. Unfortunately, the genome size estimates were only slightly improved and still far from normal (see Fig. R2, presenting results for an analysis with kmer length set at 17 and Max kmer coverage at 100,000).

To our surprise, there were marked improvements when we set the Max kmer coverage at 200,000 and kmer length at 21 unintentionally. The modeling converged successfully in the final step and the estimated genome size was 2.606 Gb; much closer to estimates obtained by the other approaches than previous GenomeScope estimates (See Fig. R3). Then we realized that both the kmer length and Max kmer coverage were key factors for GenomeScope estimates. Subsequently, we tried a higher kmer length (21-39) with Max kmer coverage ranging from 50,000 to 500,000. The estimated genome sizes we obtained were 2.575 to 2.784 Gb (See Fig. R4, showing results obtained with the kmer length set at 39 and Max kmer coverage at 100,000). Despite up to 210 Mbp discrepancies among the new GenomeScope estimates, and at least 216 Mbp discrepancy between them and GCE estimates, they were more acceptable than previous estimates.

We consulted the GenomeScope publishers regarding this issue, and obtained the following explanation from Michael Schatz, director of the GenomeScope project.
"…The model fits look pretty good for the higher kmer lengths (k=21 and above) in that the main peaks fit the model closely and lead to an estimated genome size of about 2.5 to 2.7Gbp. For k=17, it looks like the modeling got very confused about which peak represented the homozygous kmers so I would not trust that result at all. Other than k=17, the wide range in genome size this is largely because of the max kmer coverage value - we find that often very high frequency kmers are not part of the genome, but instead are some sort of contaminating sequence: phiX spikein, high coverage mitochondria sequences, microbial contaminates, etc. Even though these genomes are typically small, they can contribute to the overall genome size estimate if they occur in hundreds of thousands to millions of copies. So the goal of the max kmer coverage threshold is to exclude these very high coverage kmers from consideration. This is usually pretty effective, but can underestimate the overall genome size if there are real high coverage kmers from the genome, such as from the centromeres or telomeres … And I think it would be fair to estimate the genome size at around 2.7Gb as this is supported by multiple methods."

Accordingly, we regard the estimate obtained with Kmer set at 39 as the best estimate yielded by this approach. We have added this estimate and the corresponding parameters in our revised manuscript (Lines 85-87, Fig. S2)

4. There are several grammar errors within the manuscript which need to be corrected. I believe the manuscript would benefit from light type-editing.
Reply: We thank the reviewer for pointing out this. The paper has been edited by a professional, native English-speaking editor who has a PhD in a relevant discipline.

Powered by Editorial Manager® and ProduXion Manager® from Aries Systems Corporation

	(Please see Fig. R1-R4 in the docx file, "Milu-response-R2 letter".)<br><br>Reviewer #2: The authors have taken on board the comments that I made in my review and added additional information.<br>1. I think there is still a little confusion regarding gene nomenclature. By this I am referring to the actual symbol and description, for example:BRCA2:BRCA2, DNA repair associated. Has an actual gene set been produced and given symbols?<br>For point 4 from the reviewers comments the authors have revised and added to the gene ontology, which is useful, but not what I was referring to.<br>Reply: We thank the reviewer for highlighting this issue. For the nomenclature, we aligned the milu deer genes against coding genes of cattle. Then we regarded the gene symbol of the best match (coding gene of cattle) as the gene symbol for each aligned Milu deer gene. These gene symbols and related descriptions have been added to the gff file of this assembly (See Fig. R5, a screenshot of the mRNA lines in gff file stored in GigaDB).<br><br>Regarding point 4 of the last round of reviewer's comments, the previous version of the paper reported analyses of milu species-specific gene families and GO analysis conducted to examine their possible functions. In the revised version we have also provided a gene list of milu's specific gene families (See Table S13).<br><br>2. Related to this, point 10, a new table has been generated (S9) which shows the percentage coverage from different resources that the authors found in their gene set. This is a higher level analysis than I was referring to, but is valid and in the context of this paper seems useful.<br>The new table S11 is exactly what Is was referring to and is much more helpful.<br>Reply: We appreciate the reviewer's positive comments on our added work.<br><br>3. There is a typo on line 395: Veen (should be Venn).<br>Reply: Corrected.<br><br>(Please see Fig. R5 in the docx file, "Milu-response-R2 letter".)
**Additional Information:**	
**Question**	**Response**
Are you submitting this manuscript to a special series or article collection?	No
**Experimental design and statistics**<br><br>Full details of the experimental design and statistical methods used should be given in the Methods section, as detailed in our Minimum Standards Reporting Checklist. Information essential to interpreting the data presented should be made available in the figure legends.<br><br>Have you included all the information requested in your manuscript?	Yes
**Resources**<br><br>A description of all resources used, including antibodies, cell lines, animals and software tools, with enough information to allow them to be uniquely identified, should be included in the	Yes

Powered by Editorial Manager® and ProduXion Manager® from Aries Systems Corporation

Methods section. Authors are strongly encouraged to cite Research Resource Identifiers (RRIDs) for antibodies, model organisms and tools, where possible.<br><br>Have you included the information requested as detailed in our Minimum Standards Reporting Checklist?	
**Availability of data and materials**<br><br>All datasets and code on which the conclusions of the paper rely must be either included in your submission or deposited in publicly available repositories (where available and ethically appropriate), referencing such data using a unique identifier in the references and in the “Availability of Data and Materials” section of your manuscript.<br><br>Have you have met the above requirement as detailed in our Minimum Standards Reporting Checklist?	Yes

Manuscript

Click here to download Manuscript Milu-revised-R2 MS_FinalEd.docx

Draft genome of the milu (*Elaphurus davidianus*)

Chenzhou Zhang[1, †], Lei Chen[1, †], Yang Zhou[2, 3, †], Kun Wang[1], Leona G. Chemnick[4], Oliver A. Ryder[4], Wen Wang[1], Guojie Zhang[2, 3, 5, *], Qiang Qiu[1, *]

[1] Center for Ecological and Environmental Sciences, Key Laboratory for Space Bioscience & Biotechnology, Northwestern Polytechnical University, Xi'an, 710072, China.

[2] China National Genebank, BGI-Shenzhen, Shenzhen 518083, China

[3] BGI-Shenzhen, Shenzhen 518083, China

[4] San Diego Zoo Institute for Conservation Research, Escondido, CA 92027, USA

[5] Centre for Social Evolution, Department of Biology, Universitetsparken 15, University of Copenhagen, Copenhagen 2100, Denmark

[*]Correspondence: qiuqiang@lzu.edu.cn (QQ), zhanggj@genomics.cn (GZ)

[†]These authors contributed equally to this work.

Abstract

Background: Milu, also known as Père David's deer (*Elaphurus davidianus*), was widely distributed in East Asia but recently experienced a severe bottleneck. Only 18 survived by the end of the 19th century, and the current population of 4500 individuals was propagated from just 11 kept by the 11th British Duke of Bedford. This species is known for its distinguishable appearance, the driving force behind which is still a mystery. To aid efforts to explore these phenomena we constructed a draft genome of the species.

Findings: In total, we generated 321.86 gigabases (Gb) of raw DNA sequence from whole-genome sequencing of a male milu deer using an Illumina HiSeq 2000 platform. Assembly yielded a final genome with a scaffold N50 size of 3.03 megabases (Mb), and total length of 2.52 Gb. Moreover, we identified 20,125 protein-coding genes and 988.1 Mb of repetitive sequences. In addition, homology-based searches detected 280 rRNA, 1,335 miRNA, 1,441 snRNA and 893 tRNA sequences in the milu genome. The divergence time between *E. davidianus* and *Bos taurus* was estimated to be about 28.20 million years ago (Mya). We identified 167 species-specific genes and 293 expanded gene families in the milu lineage.

Conclusions: We report the first reference genome of milu, which will provide a valuable resource for studying the species' demographic history of severely bottlenecked and genetic mechanism of special phenotypic evolution.

Keywords: *Elaphurus davidianus*, Reference genome, Evolution

Data description

Background

Père David’s deer (*Elaphurus davidianus*), named after its western finder (Father Armand David) and called "milu" in China, was an endemic species that was once widely distributed in East Asia [1, 2]. Milu also has a colloquial name in China, *Sibuxiang*, which could be translated as "the four unlikes", because it has the hooves of a cow, head of a horse, antlers of a deer, and tail of a donkey, but is not any of these animals (**Fig. 1**). Due to intense human and natural pressures, such as excessive hunting by humans and habitat degradation, milu became extinct in China by the end of the 19th century and only 18 individuals survived in several European zoos at that time. The 18 survived individuals were collected by the 11th British Duke of Bedford, kept at Woburn Abbey (UK) and only 11 participated in subsequent reproduction [3]. After this severe bottleneck, the milu population started to recover. In the 1980s, dozens were reintroduced into China, and there were over 1,500 in China and more than 3,000 globally by 2004 [4]. Milu is highly interesting partly because of this bottleneck and partly because it has atypical features for a cervid, such as a relatively long tail and unique branched antlers. Due to these traits, scientists once identified it as the root of the subfamily Cervinae, but subsequent molecular analysis indicated that milu is closer to the genus *Cervus* [5-9]. However, little is still known about the genetic architecture underlying milu’s unique phenotypic features, and the population dynamics during its recovery from the severe bottleneck. Thus, we constructed a draft genome for the species to facilitate investigation of effects of the severe recent bottleneck, and the molecular mechanisms involved in its phenotypic evolution.

Library construction and filtering

Genomic DNA was extracted from a male milu bred at the San Diego Zoo Safari Park, Escondido, California, USA, utilizing heart tissue collected at necropsy (NCBI Taxonomy ID, 43332). The extracted DNA was used to construct short-insert libraries (170, 500 and 800 base pair, bp) and subsequently long-insert libraries (2, 5, 10 and 20 kilo base, kb). A HiSeq 2000 platform (Illumina; CA, USA) was subsequently used to sequence paired end reads of each library based on a whole genome shotgun sequencing strategy, generating 100 bp and 49 bp reads from the short-insert and long-insert libraries, respectively. In total, a 321.86 Gb raw dataset was obtained (**Table S1**).

Raw reads were filtered out that had: (1) > 5% uncalled ("N") bases or polyA structure; (2) ≥60 bases with quality scores ≤ 7 for reads generated from the short-insert library sequences; (3) ≥30 such bases for reads generated from the long-insert library sequences; (4) more than 10 bp aligned to the adapter sequence; (5) read1 and read2 (of a short-insert PE read) overlapping by ≥ 10bp, allowing 10% mismatch; (6) duplicated PCR sequences. The low-quality bases at heads or tails of reads were also trimmed. After that, the short-insert library reads were corrected using SOAPec [10], a k-mer-based error correction package. This resulted in a 244.84 Gb qualified dataset, representing about 82-fold genome coverage (**Table S1**).

Estimation of milu genome size

The milu genome size (G) was estimated by K-mer frequency distribution analysis of the short-insert library, with a 1-bp slide and k set at 17, using the formula G = k-

mer_number/k-mer_depth [10]. Here, ‘k-mer_number’ is 1,592,668,741 and the expected ‘k-mer_depth’ is 25 (**Fig. S1**). The estimated milu genome size, with these parameters, is about 3.04 Gb (**Table S2**). For comparison, we also used GCE software and the GenomeScope package to estimate the milu genome size, and obtained estimates of 3.00 Gb and 2.78 Gb, respectively [11-13] (**Fig. S2**). All these estimated genome sizes are within the range of C values (2.22 to 3.44) reported for Cervidae in the Animal Genome Size Database (http://www.genomesize.com/), indicating our estimations are credible [14] (**Table S3**).

Genome assembly

SOAPdenovo software version 2.04 (SOAPdenovo, RRID:SCR_010752) [15] was applied (with parameter settings pregraph-K 79; contig -M 1; scaff –L 200 -b 1.5 -p 40) to construct the original contigs and initial scaffolds using corrected reads for the milu genome assembly. Then we used GapCloser version 1.12 (GapCloser, RRID:SCR_015026) [10] to fill the gaps of initial scaffolds using short-insert size PE reads (170, 500 and 800 bp). The initial scaffolds were then divided into scaff-tigs by the unfilled gaps. The divided scaff-tigs were connected to final scaffolds using SSPACE version 3.0 (SSPACE, RRID:SCR_005056) [16] with the following parameters: -x 0, -z 200, -g 2, -k 2, -n 10. These final scaffolds’ gaps were also closed by GapCloser. The total length of our final milu genome assembly is 2.52 Gb, accounting for 85.71% of the estimated genome size. The final contig N50 and scaffold N50 (>2 kb) sizes are 32.71 kb and 3.03 Mb, respectively (**Table 1**).

Quality assessment

To evaluate the quality of the milu genome assembly, the filtered reads (≥ 49 bp) were aligned to the assembled genome sequences using SOAPaligner version 2.20 (SOAPaligner/soap2, RRID:SCR_005503) [15] allowing three mismatches. We also sequenced the genome of another male milu deer. The clean reads obtained from this sequencing were also aligned to the assembled genome by SOAPaligner with the same parameters. Both alignments showed high coverage of each genome base, confirming accuracy at the base level (**Fig. S3 and Fig. S4**). In addition, analysis with BUSCO version 2.0 (benchmarking universal single-copy orthologs, RRID:SCR_015008) [17] showed that the assembly included complete matches for 3,820 of 4,104 mammalian BUSCOs (indicating 93.00% completeness) (**Table S4**). FRC (Feature-response curves, version 1.3.0) [18] was then used to evaluate the trade-off between its contiguity and correctness. FRC curves generated by the software showed that our milu genome assembly has similar correctness to published genomes of another three ruminants: domestic goat (*Capra hircus*, ARS1, GenBank ID: GCF_001704415.1) [19], sheep (*Ovis aries*, Oar_v3.1) and cattle (*Bos taurus* UMD3.1) (**Fig. S5**) [20, 21]. Subsequently, synteny analysis was applied to identify differences between the assembled genome and the domestic goat (*Capra hircus*) genome using MUMmer (version 3.23) [22], with a 50% identity cutoff for MUMs in the NUCmer alignments used to determine synteny **(Fig. S6**). 99.35% of the two genome sequences could be 1:1 aligned. In addition, we compared the milu and goat genomes using LAST version 3 (LAST,

RRID:SCR_006119) [23] to find the breakpoints (edges of structural variation). The overall density of different types of breakpoints was about 54.76 per Mb, comparable to densities reported in another study (**Table S5**) [24], and the average nuclear distance (percentage of different base pairs in the syntenic regions) was 6.56% (**Fig. S7**). The results indicated that the milu genome assembly has good completeness and continuity.

Repeat annotation

To annotate repeats, we first searched the milu genome for tandem repeats using Tandem Repeats Finder (version 4.04) [25] with the following settings: Match = 2, Mismatch = 7, Delta = 7, PM = 80, PI = 10, Minscore = 50. Then, RepeatMasker version 3.3.0 (RepeatMasker, RRID:SCR_012954) and RepeatProteinMask (version 3.3.0, a package in RepeatMasker) [26] were used to find known transposable element (TE) repeats in the Repbase TE library (version 16.01) [27]. In addition, RepeatModeler version 1.0.5 (RepeatModeler, RRID:SCR_015027) and LTR_FINDER version 1.0.5 (LTR_Finder, RRID:SCR_015247) [28] were used to construct a *de novo* repeat library and RepeatMasker was employed to find homolog repeats in the genome and classify the detected repeats. The results indicated that long interspersed elements accounted for 27.05% of the milu genome, and other identified repeat sequences for a further 13.99% **(Table S6).**

Gene annotation

To annotate structures and functions of putative genes in our milu genome assembly we

used both homology-based and *de novo* predictions. For homology-based predictions, homologous proteins of *Homo sapiens* (Ensembl 89 release), *Bos taurus* and *Sus scrofa* (Ensembl 89 release) were aligned to the repeat-masked milu genome using TblastN (Blastall 2.2.23) with an E-value cutoff of 1e-5. Then aligned sequences and corresponding query proteins were filtered and passed to GeneWise version 2.2.0 (GeneWise, RRID:SCR_015054) [29] for accurate spliced alignments. Gene sequences shorter than 150 bp, and frame-shifted or prematurely terminated genes, were removed. *De novo* predictions were obtained from analysis of the repeat-masked genome using Augustus version 2.5.5 (Augustus: Gene Prediction, RRID:SCR_008417) [30] and GENSCAN version 1.0 (GENSCAN, RRID:SCR_012902) [31], with parameters generated from training with *Homo sapiens* genes. The filter processes applied in the homology-based prediction procedure were also applied in the *de novo* predictions. Next, the obtained results were integrated using GLEAN (version 1.0.1) [32], then genes with few exons (≤ 3), which could not be aligned well in SwissProt or TrEMBL, were filtered to produce a final consensus gene set containing 20,125 genes. The number of genes, gene length distribution, exon number per gene and intron length distribution were similar to those of other mammals **(Fig. S8 and Table S7).** We also identified a total of 2,803 pseudogenes from GeneWise alignment, of which 2,801 had prematurely terminating mutations, and 1,358 had frame-shifted mutations **(Table S8 and S9)** [29].

Then, the KEGG, SwissProt and TrEMBL databases were searched for best matches to the final gene set using BLASTP (version 2.2.26) with an E-value of 1e-5.

Subsequently, InterProScan software version 5.18-57.0 (InterProScan, RRID:SCR_005829) was applied to map putative encoded protein sequences against entries in the Pfam, PRINTS, ProDom and SMART databases to identify known motifs and domains. In total, at least one function was allocated to 17,913 (89.31%) of the genes in this manner (**Table S10**). Next, reads from the short-insert library with about 27-fold genome coverage were mapped to the milu genome using BWA version 0.7.15-r1140 (BWA, RRID:SCR_010910) [33] and subsequently called variants by SAMtools (version 1.3.1) [34]. Finally, SnpEff (version 4.10) [35] was applied to identify the distribution of single nucleotide variants (SNVs) in the milu genome (**Table S11**).

In addition, putative short noncoding RNAs were identified by BLASTN alignment of human rRNA sequences with milu homologs. We employed Infernal version 0.81 (Infernal, RRID:SCR_011809) with the Rfam database (release 9.1) to annotate the miRNA and snRNA genes. The tRNAs were annotated using tRNAscan-SE version 1.3.1 (tRNAscan-SE, RRID:SCR_010835) with default parameters. In total, 3,949 short noncoding RNA sequences were identified in the milu deer genome (**Table S12**).

Species-specific genes and phylogenetic relationships

The detected milu genes were clustered in families using OrthoMCL version 2.0.9 (OrthoMCL DB: Ortholog Groups of Protein Sequences, RRID:SCR_007839) [36] with an E-value cutoff of 1e-5, and Markov Chain Clustering with a default inflation parameter in an all-to-all BLASTP analysis of entries for five species (*Homo sapiens*,

Equus caballus, *Capra hircus*, *Bos taurus*, and *Elaphurus davidianus*). The results indicated that 69 gene families and 167 genes were specific to milu (**Fig. 2a, Table S13**). We also detected 293 gene families that had apparently expanded in the milu lineage using CAFÉ (Computational Analysis of gene Family Evolution, version 4.0.1) [37]. The milu species-specific gene families were enriched in four GO categories related to hormone activity, pinocytosis, ribosome and structural constituent of ribosomes **(Table S14)**. The expanded gene families were enriched in 34 GO categories: motor activity, ATPase activity, calcium ion binding and 31 others (**for details, see Table S15**). Subsequently, 7,906 one-to-one orthologs were identified from these species and aligned using PRANK (version 3.8.31) [38]. Next, we extracted 4D-sites (four-fold degenerate sites) to construct a phylogenetic tree by RAxML version 7.2.8 (RAxML, RRID:SCR_006086) [39] with the GTR+G+I model. Finally, phylogenetic analysis by PAML MCMCtree (version 4.5) [40], calibrated with published timings for the divergence of the reference species (http://www.timetree.org/), showed that *Elaphurus davidianus*, *Bos taurus* and *Capra hircus* diverged from a common ancestor approximately 28.20 million years ago (**Fig. 2b**).

In summary, we report the first sequencing, assembly, and annotation of the milu genome. The assembled draft genome will provide a valuable resource for studying the species' evolutionary history, as well as genetic changes and associated phenomena, such as genetic load and selection pressures that occurred during its severe bottleneck or other unknown historical events. It should be noted that this draft assembly was generated by NGS data and there may be some errors in highly GC-biased or repeated

regions. Moreover, this genome assembly should be elevated to chromosomal level in the future with Hi-C, optical mapping or genetic mapping technologies.

Supporting data

The raw reads of each sequencing library have been deposited at NCBI, Project ID: PRJNA391565. For the assembled individual (Sample ID: SAMN07270940), please refer to the SRA accession numbers in Table S1. The SRA accession number for the other sequenced individual (Sample ID: SAMN08014286) is SRR6287186. The assembly and annotation of the milu genome, together with further supporting data, are available via the *GigaScience* GigaDB database [41]. Supplementary Figures and Tables are provided in additional file 1.

Abbreviations

Gb, giga base; bp, base pair; kb, kilo base; Mb, mega base; TE, transposable element; BUSCO, benchmarking universal single-copy orthologs; FRC, feature-response curves; SNV, single nucleotide variant;

Acknowledgements

This study was supported by grants from the Talents Team Construction Fund of Northwestern Polytechnical University (NWPU) to QQ and WW. We thank Nowbio Biotech Inc., Kunming, China, for excellent work on DNA library construction and assistance during the genome sequencing. This project was initiated under the auspices

of the Genome 10K Project.

Authors’ contributions

Q.Q. W.W. and G.Z. conceived the study. C.Z. and L.C. designed the scientific objectives. L.G.C and O.A.R evaluated and provided samples from San Diego Zoo Global. Y.Z. collected the samples, extracted the genomic DNA and constructed the DNA libraries. C.Z. and Y.Z. estimated the milu genome size and assembled the genome. C.Z., L.C. and Y.Z. carried out the quality assessment, repeat annotation and gene annotation. C.Z. and K.W. were responsible for finding species-specific genes and phylogenetic relationship construction. L.C. and C.Z. uploaded the raw read data, genome assembly and annotation in NCBI and *GigaScience* GigaDB databases. C.Z., Q.Q. and W.W. wrote the manuscript. Q.Q., W.W. and G.Z. supervised all aspects of the work to ensure the accuracy and integrity of the research and data. O.A.R contributed to editing the final manuscript. All authors read and approved the final manuscript.

Ethics statement

Animal collection and utility protocols were approved by the Northwestern Polytechnical University and BGI-Shenzhen Laboratory Animal Care and Use Committee, and were in accordance with guidelines from the China Council on Animal Care. Samples provided by San Diego Zoo Global (SDZG) were collected in accordance with SDZG's Institutional Animal Care and Use Committee policies, which

meet or exceed U.S. regulatory standards for the humane care and treatment of animals in research.

Competing interests

The authors declare that they have no competing interests

References:

1. Harrison RJ, Hamilton WJ. The reproductive tract and the placenta and membranes of Père David's deer (*Elaphurus davidianus* Milne Edwards). Journal of Anatomy. 1952; 86(2):203-225.
2. Cao K. On the time of extinction of the wild Mi-deer in China (in Chinese). Acta Zoologica Sinica. 1978; 24(3):289-291.
3. JONES F. A contribution to the history and anatomy of Père David's Deer (*Elaphurus davidianus*). Journal of Zoology. 1951; 2(121):319-370, doi:10.1111/j.1096-3642.1951.tb00800.x.
4. Ding Y. Chinese milu research (in Chinese). Changchun, China: Jilin Publishing House for the Science and Technology; 2004.
5. Tate ML, Mathias HC, Fennessy PF, Dodds KG, Penty JM, Hill DF. A new gene mapping resource: interspecies hybrids between Pere David's deer (*Elaphurus davidianus*) and red deer (*Cervus elaphus*). Genetics. 1995; 139(3):1383-1391.
6. Slate J, Van Stijn TC, Anderson RM, McEwan KM, Maqbool NJ, Mathias HC, Bixley MJ, Stevens DR, Molenaar AJ, Beever JE *et al*. A deer (subfamily Cervinae) genetic linkage map and the evolution of ruminant genomes. Genetics. 2002; 160(4):1587-1597.
7. Pitra C, Fickel J, Meijaard E, Groves PC. Evolution and phylogeny of old world deer. Molecular Phylogenetics and Evolution. 2004; 33(3):880-895, doi:10.1016/j.ympev.2004.07.013.
8. Maqbool NJ, Tate ML, Dodds KG, Anderson RM, McEwan KM, Mathias HC, McEwan JC, Hall RJ. A QTL study of growth and body shape in the inter-species hybrid of Pere David's deer (*Elaphurus davidianus*) and red deer (*Cervus elaphus*). Animal Genetics. 2007; 38(3):270-276, doi:10.1111/j.1365-2052.2007.01597.x.
9. Emerson BC, Tate ML. Genetic analysis of evolutionary relationships among deer (subfamily Cervinae). Journal of Heredity. 1993; 84(4):266-273.
10. Li R, Fan W, Tian G, Zhu H, He L, Cai J, Huang Q, Cai Q, Li B, Bai Y *et al*. The sequence and de novo assembly of the giant panda genome. Nature. 2010; 463(7279):311-317, doi:10.1038/nature08696.
11. Liu B, Shi Y, Fan W. Estimation of genomic characteristics by analyzing k-mer frequency in de novo Estimation of genomic characteristics by analyzing k-mer frequency in de novo genome projects. arXiv preprint. 2013; arXiv:1308.2012.
12. Vurture GW, Sedlazeck FJ, Nattestad M, Underwood CJ, Fang H, Gurtowski J, Schatz MC. GenomeScope: fast reference-free genome profiling from short reads. Bioinformatics. 2017; 33(14):2202-2204, doi:10.1093/bioinformatics/btx153.
13. Marcais G, Kingsford C. A fast, lock-free approach for efficient parallel counting of occurrences of k-mers. Bioinformatics. 2011; 27(6):764-770, doi:10.1093/bioinformatics/btr011.
14. Gregory, T.R. Animal Genome Size Database. http://www.genomesize.com,

2017.

15. Li R, Zhu H, Ruan J, Qian W, Fang X, Shi Z, Li Y, Li S, Shan G, Kristiansen K *et al*. De novo assembly of human genomes with massively parallel short read sequencing. Genome Research. 2010; 20(2):265-272, doi:10.1101/gr.097261.109.

16. Boetzer M, Henkel CV, Jansen HJ, Butler D, Pirovano W. Scaffolding pre-assembled contigs using SSPACE. Bioinformatics. 2011; 27(4):578-579, doi:10.1093/bioinformatics/btq683.

17. Simao FA, Waterhouse RM, Ioannidis P, Kriventseva EV, Zdobnov EM. BUSCO: assessing genome assembly and annotation completeness with single-copy orthologs. Bioinformatics. 2015; 31(19):3210-3212, doi:10.1093/bioinformatics/btv351.

18. Vezzi F, Narzisi G, Mishra B. Reevaluating assembly evaluations with feature response curves: GAGE and assemblathons. PLoS One. 2012; 7(12):e52210, doi:10.1371/journal.pone.0052210.

19. Bickhart DM, Rosen BD, Koren S, Sayre BL, Hastie AR, Chan S, Lee J, Lam ET, Liachko I, Sullivan ST *et al*. Single-molecule sequencing and chromatin conformation capture enable de novo reference assembly of the domestic goat genome. Nature Genetics. 2017; 49(4):643-650, doi:10.1038/ng.3802.

20. Jiang Y, Xie M, Chen W, Talbot R, Maddox JF, Faraut T, Wu C, Muzny DM, Li Y, Zhang W *et al*. The sheep genome illuminates biology of the rumen and lipid metabolism. Science. 2014; 344(6188):1168-1173, doi:10.1126/science.1252806.

21. Elsik CG, Tellam RL, Worley KC, Gibbs RA, Muzny DM, Weinstock GM, Adelson DL, Eichler EE, Elnitski L, Guigo R *et al*. The genome sequence of taurine cattle: a window to ruminant biology and evolution. Science. 2009; 324(5926):522-528, doi:10.1126/science.1169588.

22. Delcher AL, Salzberg SL, Phillippy AM. Using MUMmer to identify similar regions in large sequence sets. Current Protocols in Bioinformatics. 2003; Chapter 10:10-13, doi:10.1002/0471250953.bi1003s00.

23. Kielbasa SM, Wan R, Sato K, Horton P, Frith MC. Adaptive seeds tame genomic sequence comparison. Genome Research. 2011; 21(3):487-493, doi:10.1101/gr.113985.110.

24. Wang K, Wang L, Lenstra JA, Jian J, Yang Y, Hu Q, Lai D, Qiu Q, Ma T, Du Z *et al*. The genome sequence of the wisent (Bison bonasus). Gigascience. 2017, 6(4):1-5, doi:10.1093/gigascience/gix016.

25. Benson G. Tandem repeats finder: a program to analyze DNA sequences. Nucleic Acids Research. 1999; 27(2):573-580.

26. Tarailo-Graovac M, Chen N. Using RepeatMasker to identify repetitive elements in genomic sequences. Current Protocols in Bioinformatics. 2009; Chapter 4:4-10, doi:10.1002/0471250953.bi0410s25.

27. Jurka J, Kapitonov VV, Pavlicek A, Klonowski P, Kohany O, Walichiewicz J. Repbase Update, a database of eukaryotic repetitive elements. Cytogenetic and Genome Research. 2005; 110(1-4):462-467, doi:10.1159/000084979.

28. Xu Z, Wang H. LTR_FINDER: an efficient tool for the prediction of full-length LTR retrotransposons. Nucleic Acids Research. 2007; 35:W265-W268, doi:10.1093/nar/gkm286.
29. Birney E, Clamp M, Durbin R. GeneWise and Genomewise. Genome Research. 2004; 14(5):988-995, doi:10.1101/gr.1865504.
30. Stanke M, Keller O, Gunduz I, Hayes A, Waack S, Morgenstern B. AUGUSTUS: ab initio prediction of alternative transcripts. Nucleic Acids Research. 2006; 34:W435-W439, doi:10.1093/nar/gkl200.
31. Burge C, Karlin S. Prediction of complete gene structures in human genomic DNA. Journal of Molecular Biology. 1997; 268(1):78-94, doi:10.1006/jmbi.1997.0951.
32. Elsik CG, Mackey AJ, Reese JT, Milshina NV, Roos DS, Weinstock GM. Creating a honey bee consensus gene set. Genome Biology. 2007; 8(1):R13, doi:10.1186/gb-2007-8-1-r13.
33. Li H. Aligning sequence reads, clone sequences and assembly contigs with BWA-MEM. arXiv preprint arXiv:13033997. 2013.
34. Li H, Handsaker B, Wysoker A, Fennell T, Ruan J, Homer N, Marth G, Abecasis G, Durbin R. The Sequence Alignment/Map format and SAMtools. Bioinformatics. 2009; 25(16):2078-2079, doi:10.1093/bioinformatics/btp352.
35. Cingolani P, Platts A, Wang LL, Coon M, Nguyen T, Wang L, Land SJ, Lu X, Ruden DM. A program for annotating and predicting the effects of single nucleotide polymorphisms, SnpEff: SNPs in the genome of Drosophila melanogaster strain w1118; iso-2; iso-3. Fly (Austin). 2012; 6(2):80-92, doi:10.4161/fly.19695.
36. Li L, Stoeckert CJ, Roos DS. OrthoMCL: identification of ortholog groups for eukaryotic genomes. Genome Research. 2003; 13(9):2178-2189, doi:10.1101/gr.1224503.
37. De Bie T, Cristianini N, Demuth JP, Hahn MW. CAFE: a computational tool for the study of gene family evolution. Bioinformatics. 2006; 22(10):1269-1271, doi:10.1093/bioinformatics/btl097.
38. Loytynoja A, Goldman N. An algorithm for progressive multiple alignment of sequences with insertions. Proceedings of the National Academy of Sciences of the United States of America. 2005; 102(30):10557-10562, doi:10.1073/pnas.0409137102.
39. Stamatakis A. RAxML version 8: a tool for phylogenetic analysis and post-analysis of large phylogenies. Bioinformatics. 2014; 30(9):1312-1313, doi:10.1093/bioinformatics/btu033.
40. Yang Z. PAML 4: phylogenetic analysis by maximum likelihood. Molecular Biology and Evolution. 2007; 24(8):1586-1591, doi:10.1093/molbev/msm088.
41. Zhang C, Chen L, Zhou Y, Wang K, Chemnick LG, Ryder OA, et al. Supporting data for "Draft genome of the milu (Elaphurus davidianus)". *GigaScience* Database 2017. http://dx.doi.org/10.5524/100383

.

Figure legends

Figure 1: Photo of two fighting Père David's deer in Dafeng Milu National Reserves, Jiangsu, China. A red wound was spotted on the body of the deer to the right, and winning such fights generally increases mating chances.

Figure 2. Phylogenetic relationships and genomic comparisons. (a) A Venn diagram of the orthologues shared among *Elaphurus davidianus, Equus caballus, Capra hircus, Bos taurus,* and *Homo sapiens.* Each number represents a gene family number and the sum of the numbers in the green, yellow, brown, red and blue areas indicate total numbers of the gene families in milu, horse, human, goat and cattle genomes, respectively. (b) Divergence time estimates for the five species generated using MCMCtree and the 4-fold degenerate sites; the dots correspond to calibration points and the divergence times were obtained from http://www.timetree.org/; blue nodal bars indicate 95% confidence intervals.

Table 1: Statistics of the assembled sequence length.

	Contig		Scaffold	
	**Size (bp)**	**Number**	**Size (bp)**	**Number**
**N90**	8,530	77,768	520,987	978
**N80**	14,483	55,968	1,045,447	647
**N70**	20,193	41,646	1,614,103	455
**N60**	26,169	30,975	2,222,401	322
**N50**	32,707	22,564	3,039,716	223
**Longest**	292,964	----	17,945,643	----
**Total Size**	2,460,119,591	----	2,524,831,955	----
**Percent of unknown bases**	----	----	2.56%	
**Total Number (≥100 bp)**	----	189,067	----	46,381
**Total Number (≥2 kb)**	----	118,986	----	4,772

Additional files

Figure S1: K-mer (k=25) distribution in the milu genome.

Figure S2: GenomeScope K-mer profile plot of the milu genome.

Figure S3: Sequence depth distribution of the assembly data.

Figure S4: Sequence depth distribution of the assembly data for the genome of the other sequenced individual.

Figure S5: Feature-response (FR) curves of four ruminant genome assemblies.

Figure S6: Visualized synteny between the milu and goat genomes.

Figure S7: DNA sequence divergence between milu and goat.

Figure S8: Comparison of gene lengths, intron lengths, exon lengths and exon numbers in milu, cattle, human and sheep genomes.

Table S1: Summary of sequenced reads.

Table S2: 17-mer depth distribution.

Table S3: Summary of the C values of Cervidae and estimated milu genome sizes.

Table S4: Summary of BUSCO analysis of matches to the 4,104 mammalian BUSCOs.

Table S5: Summary of breakpoints between milu and goat genomes.

Table S6: TE contents in the assembled milu genome.

Table S7: General statistics of predicted protein-coding genes.

Table S8: Summary of the predicted pseudogenes.

Table S9: List of predicted pseudogenes.

Table S10: Summary statistics of gene function annotation.

Table S11: Distribution of single nucleotide variants (SNVs) in the milu genome.

Table S12: Summary of short ncRNA annotation.

Table S13: List of milu species-specific genes.

Table S14: GO term enrichment in milu species-specific genes.

Table S15: GO term enrichment in gene families that have expanded in milu.

Figure 1 Click here to download Figure Figure 1.jpg

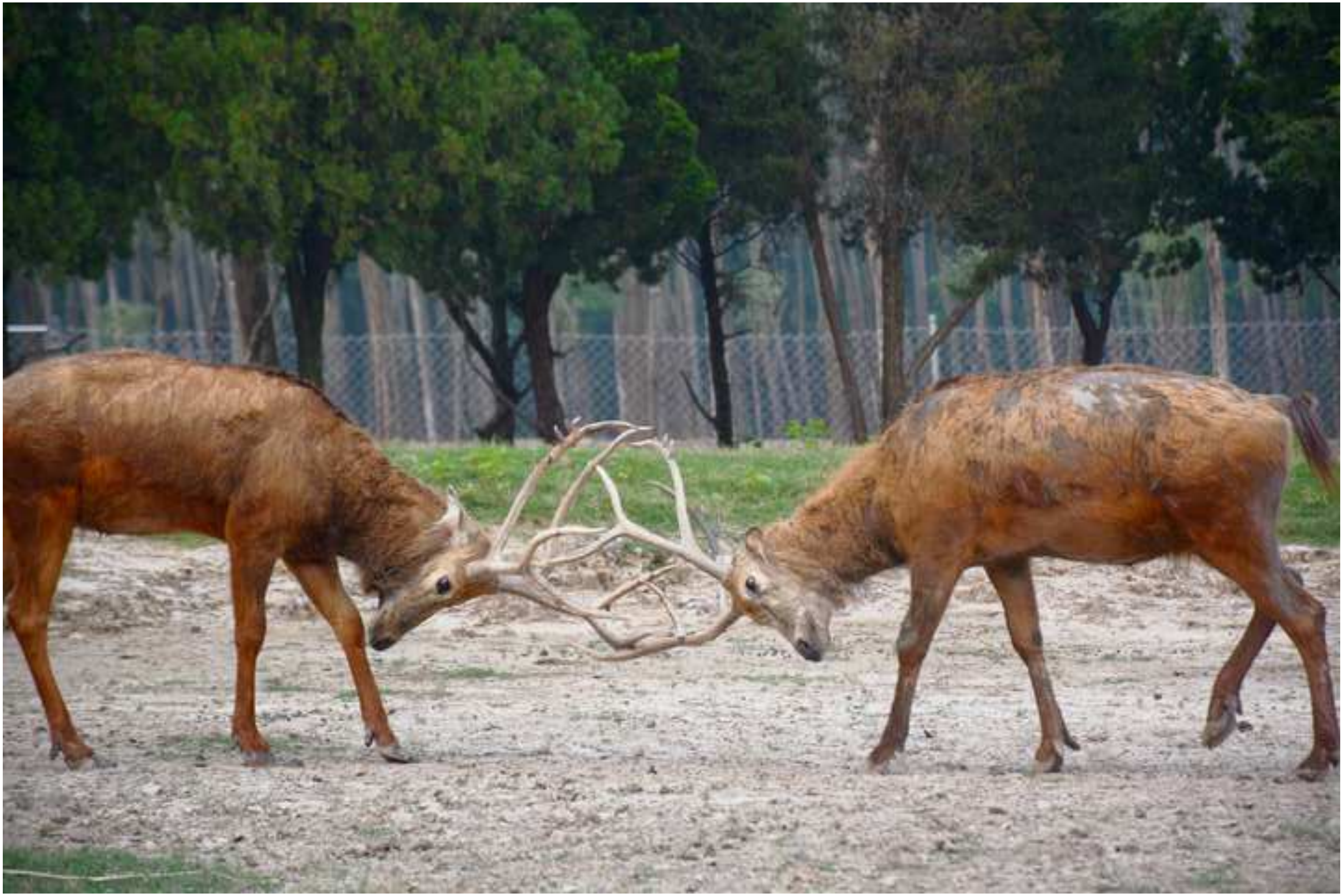

Figure 2

Click here to download Figure Figure 2.jpg

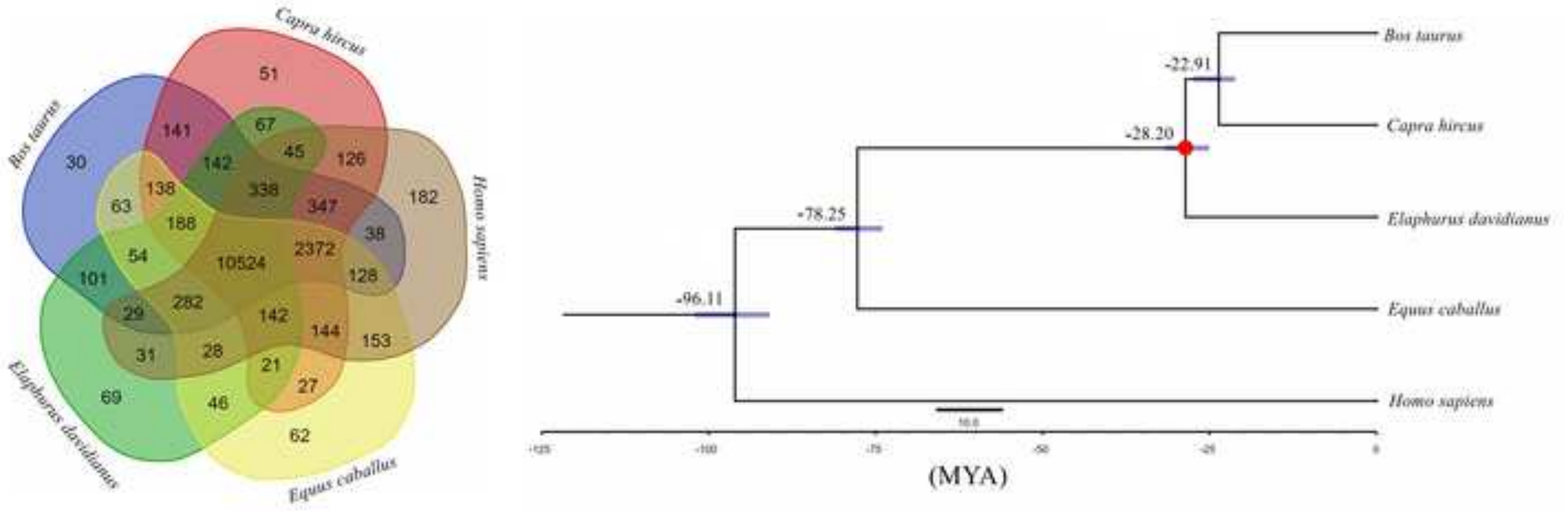

(a)

(b)

Click here to access/download

Supplementary Material

Suplementary files.pdf

Click here to access/download
Supplementary Material
Table_S9.xlsx

Supplementary Material

Click here to access/download
Supplementary Material
Table_S13.xlsx

Click here to access/download
Supplementary Material
Table_S14.xlsx

Click here to access/download
Supplementary Material
Table_S15.xlsx

Response letter

Click here to access/download
Supplementary Material
Milu-response-R2 letter.docx

Dear Dr. Hans Zauner,

Thank you very much for returning our manuscript (GIGA-D-17-00161R1) entitled "Draft genome of the milu (*Elaphurus davidianus*)", together with your helpful comments and those of the referees.

We are grateful for the reviewers' constructive and thoughtful comments, which have helped us to improve our study. We have revised the manuscript according to their suggestions and respond to their comments point-by-point (our responses are indicated in **BOLD** type).

We submit here the revised manuscript and hope that it is now suitable for publication in *GigaScience*. If you have any questions, please do not hesitate to contact the corresponding author at any time.

Thank you again for your time and effort in handling our manuscript.

Qiang Qiu
Center for Ecological and Environmental Sciences,
Northwestern Polytechnical University,
Xi'an, China.
