# Supplementary material for: Draft genome of the milu (Elaphurus davidianus)
Source: Gigascience. 2017 Dec 18;7(2):gix130. doi: 10.1093/gigascience/gix130 (PMC5824821; doi:10.1093/gigascience/gix130)
Supplement: Supplemental Figures and Tables [file gix130_supp.pdf]

Additional files

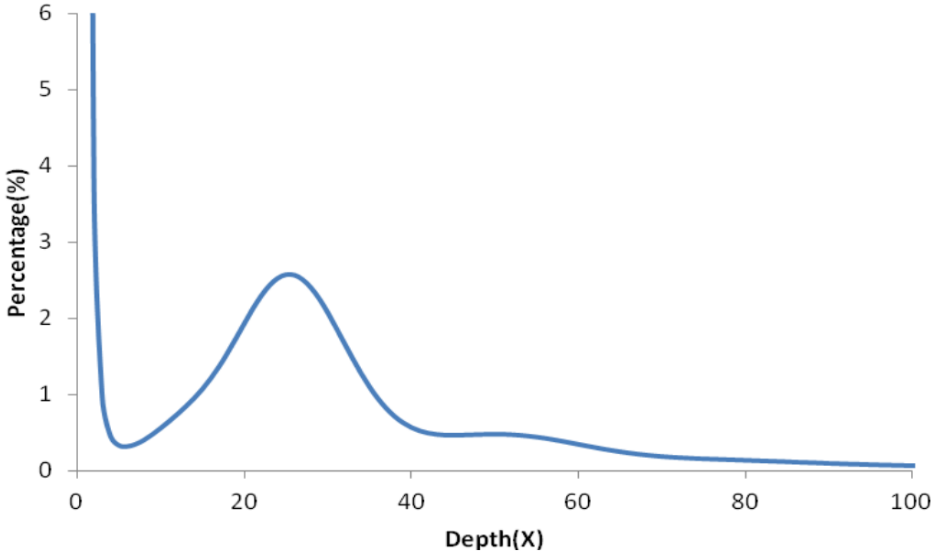

Figure S1: K-mer (k=25) distribution in the milu genome. The x-axis is depth (X); the y-axis is the proportion which represents the frequency at that depth divide by the total frequency of all the depth.

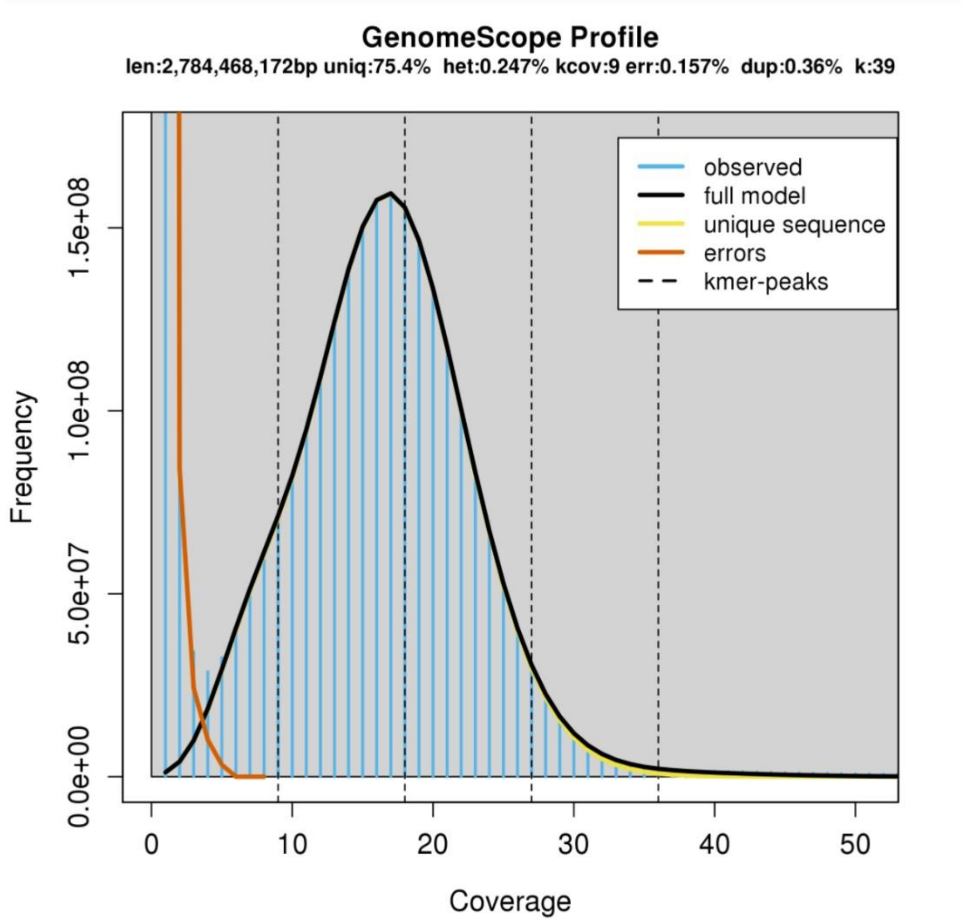

Figure S2: GenomeScope K-mer profile plot of milu genome. "len" represents the estimated genome size. The first two steps of this estimate were carried out by Jellyfish with parameters (jellyfish count –C –disk –U 100,000 -m 39 –t 20; jellyfish histo –h 100,000) inputting all clean short-insert reads. These two steps were ran to obtain the k-mer spectrum. The last step was performed by GenomeScope online running with parameters (Kmer length 21, Max kmer coverage 100,000).

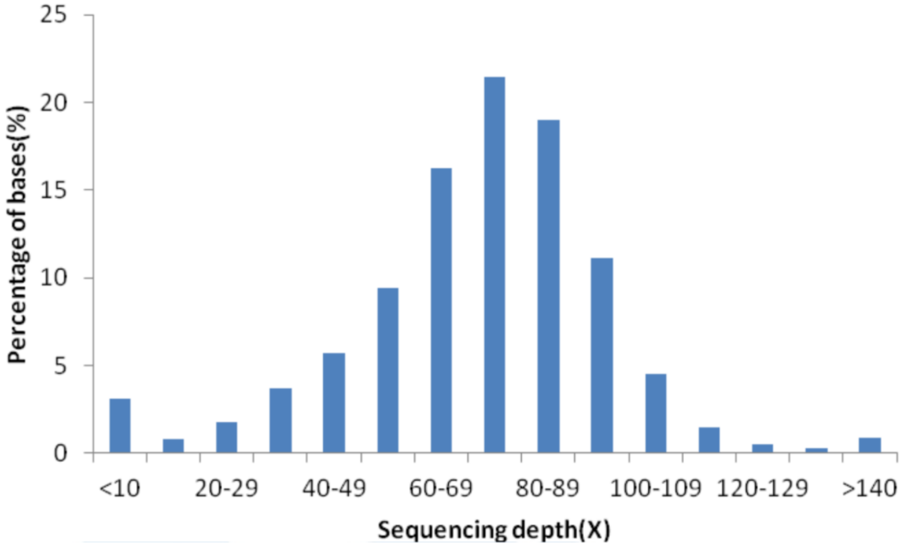

Figure S3: Sequence depth distribution of the assembly data. The x-axis shows the depth and the y-axis shows the proportion of total bases at given depths. The results demonstrate that <4% of bases had a sequencing depth less than 10, confirming accuracy at base level.

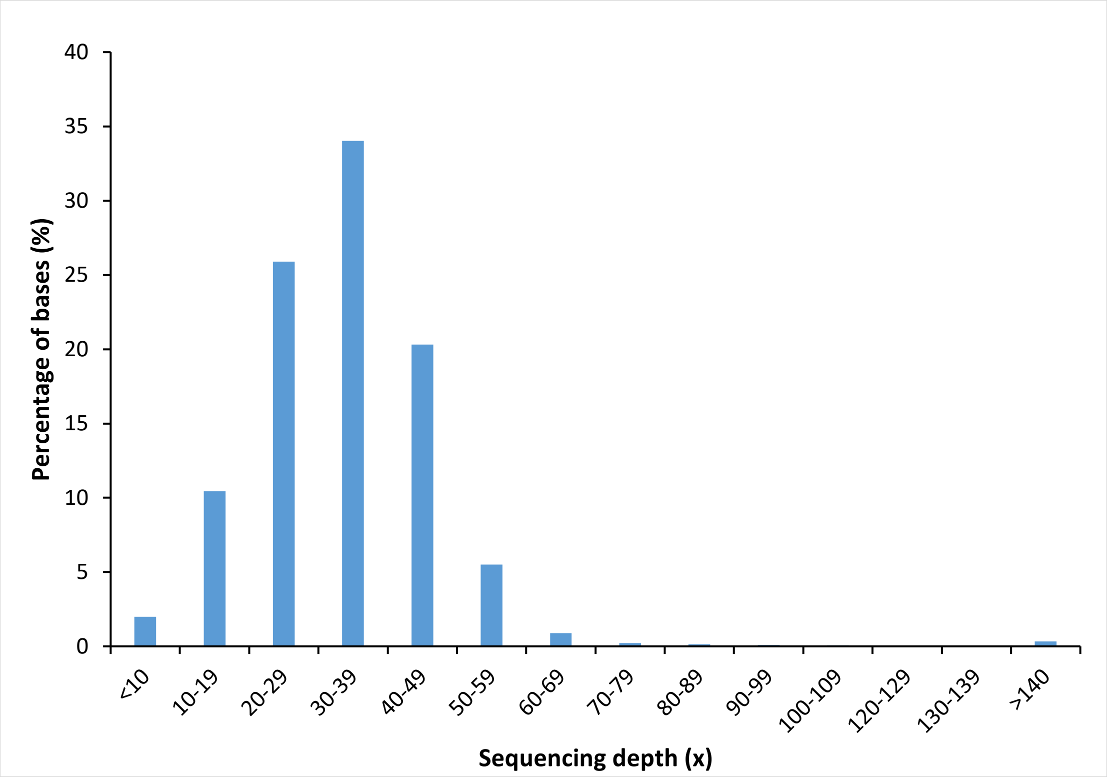

Figure S4: Sequence depth distribution of the assembly data for the genome of the other sequenced individual. The results showed that 2% of bases had a sequencing depth less than 10.

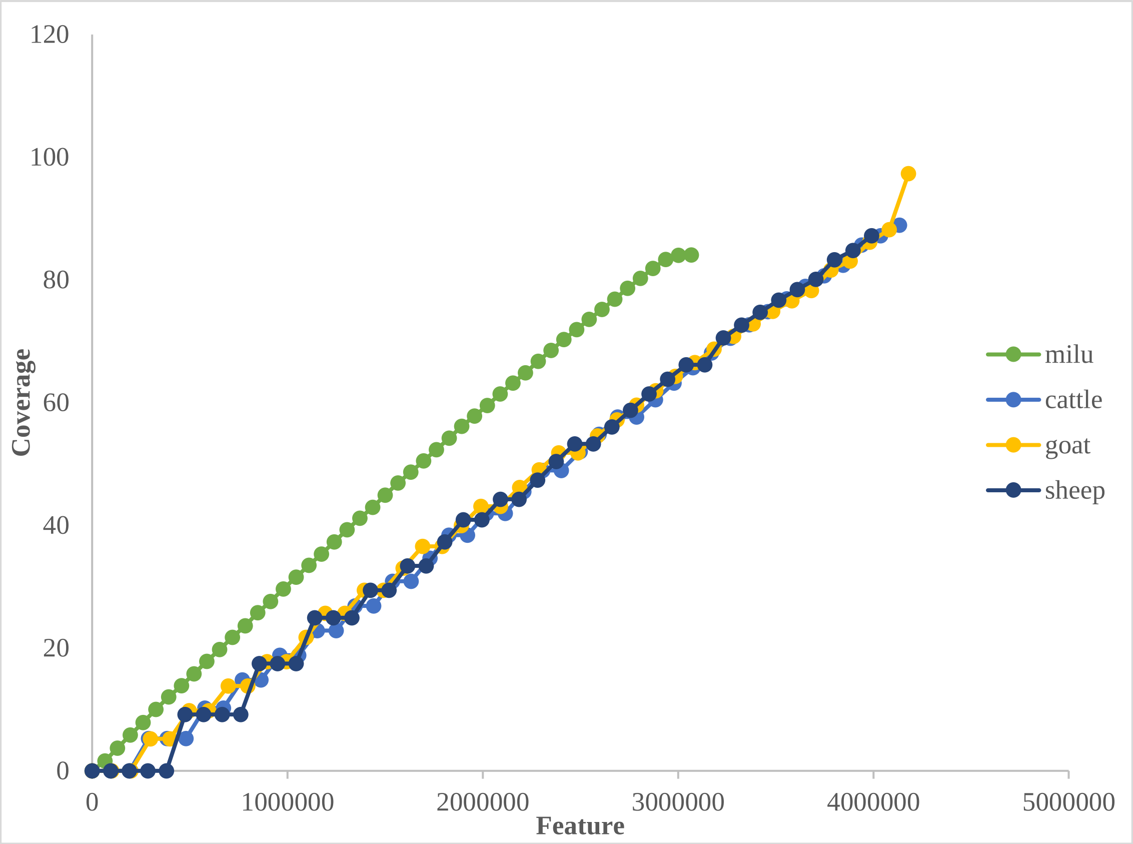

Figure S5: Feature-response (FR) curves of four ruminant genome assemblies. The curves were calculated using FRC_align (https://github.com/vezzi/FRC_align) software. The accumulation shape of the milu genome assembly is sharper than the others.

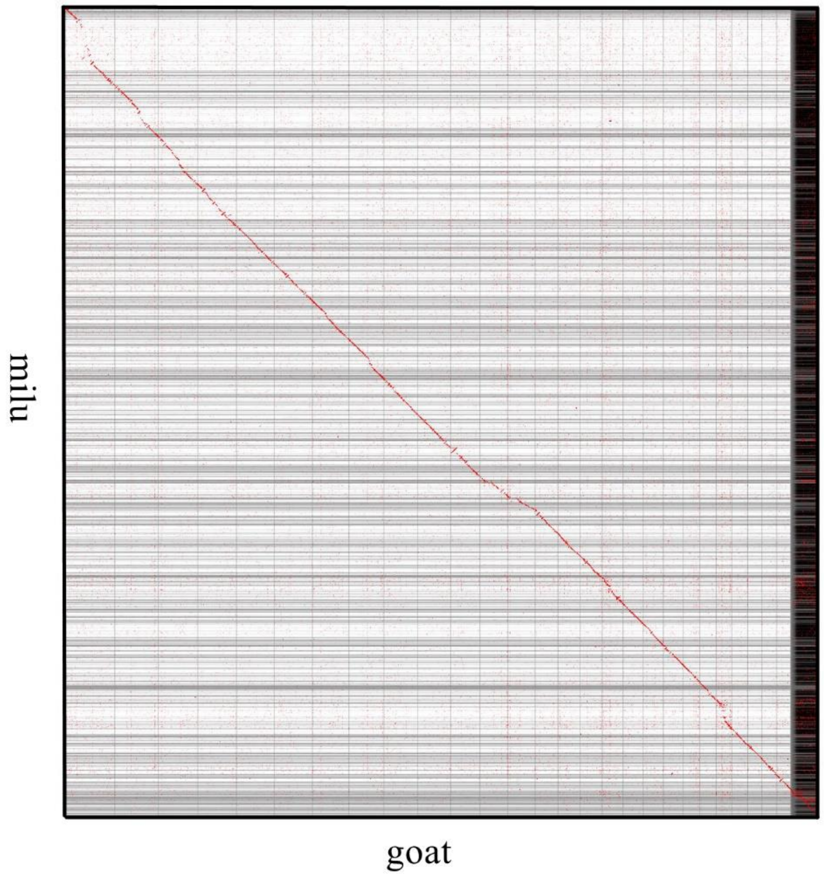

Figure S6: Visualized synteny between the milu and goat genomes. The results show that most of the milu genome is homologous to sequences in the goat genome.

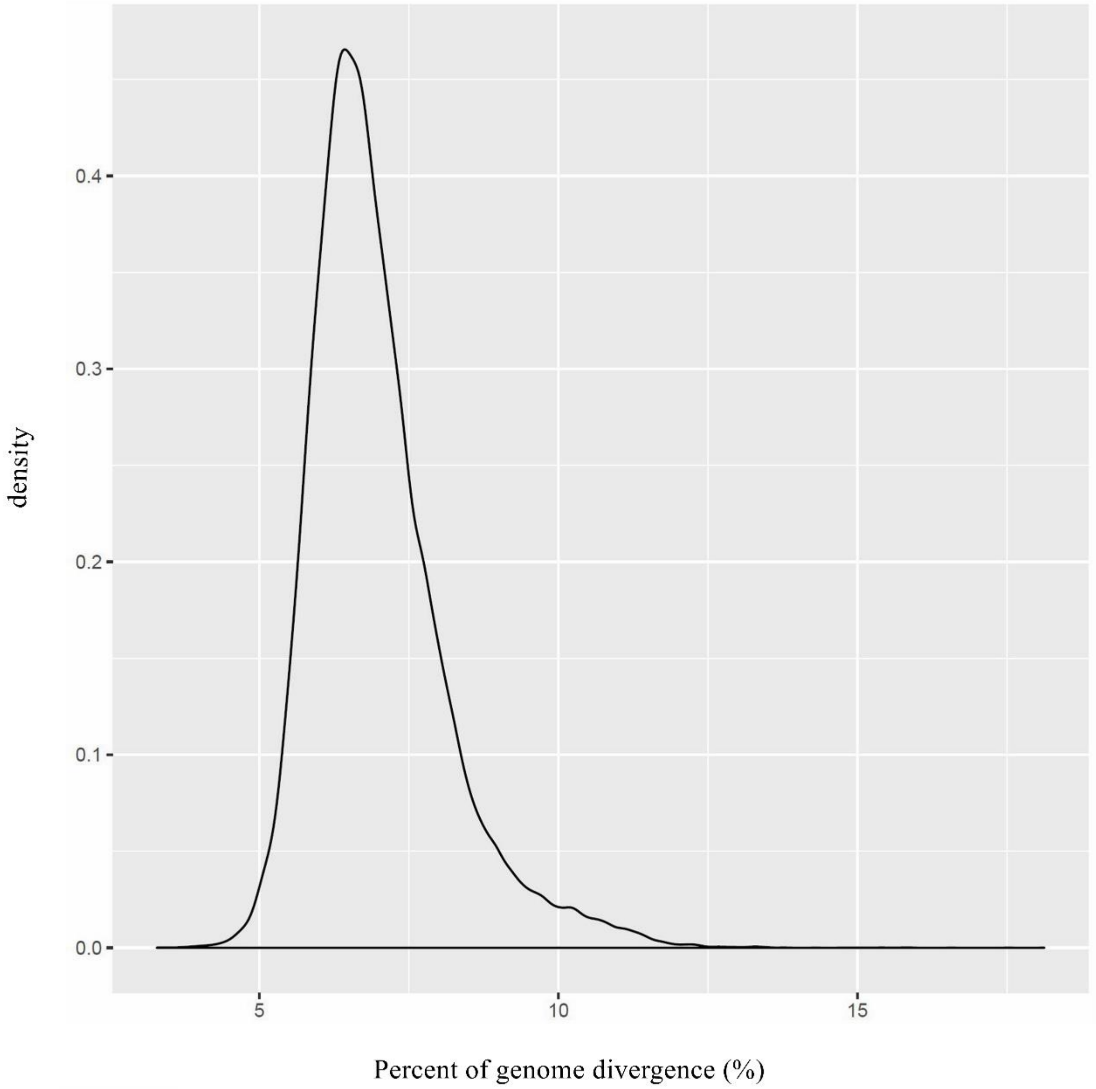

Figure S7: DNA sequence divergence between milu and goat. The x-axis shows the percentage of different base pairs in syntenous regions. The results indicate that the divergence between milu and goat genomes is 6.56%.

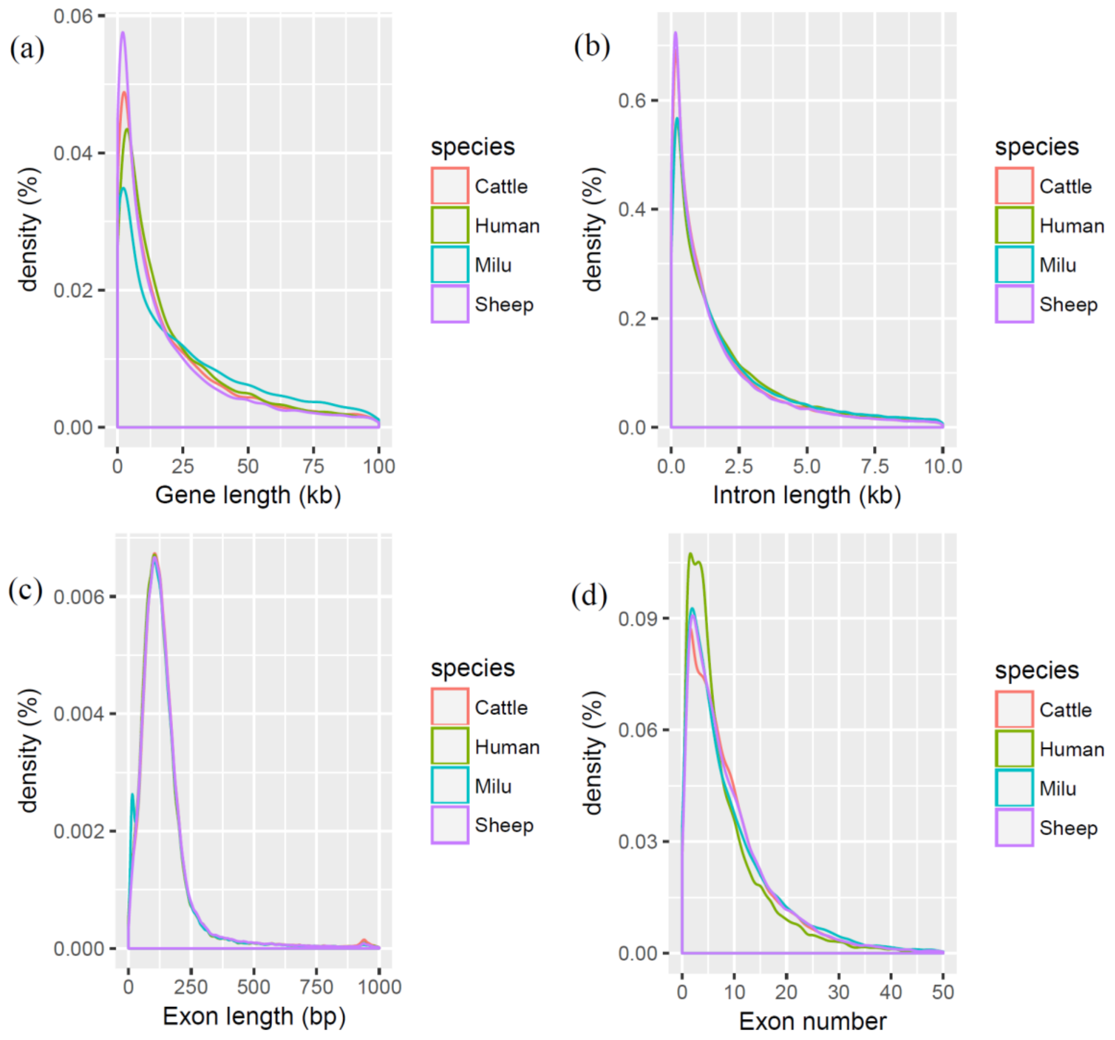

Figure S8: Comparison of gene lengths, intron lengths, exon lengths and exon numbers in the milu, cattle, human and sheep genomes. (a) Gene length, (b) Intron length, (c) Exon length, (d) Exon number per gene. The x-axis represents length or number and the y-axis represents the density of genes.

Table S1: Summary of sequenced reads.

		Raw Reads			Qualified Reads[1]			
**library Insert Size (bp)**	**Reads Length (bp)**	**Total Data (Gb)**	**Sequence Depth (X)[2]**	**Physical Depth (X)[2]**	**Total Data (Gb)**	**Sequence Depth (X)[2]**	**Physical Depth (X)[2]**	**SSR number**
170	100	89.75	29.92	25.43	80.55	26.85	23.23	SRR5762667<br>SRR5762666<br>SRR5762669<br>SRR5762668<br>SRR5762663
500	100	53.73	17.91	44.78	48.04	16.01	40.83	SRR5762662<br>SRR5762665<br>SRR5762664
800	100	37.19	12.4	49.59	30.37	10.12	42.67	SRR5762661<br>SRR5762660<br>SRR5762659
2,000	49	57.11	19.04	388.5	44.06	14.69	299.76	SRR5762658<br>SRR5762657<br>SRR5762656<br>SRR5762655<br>SRR5762654
5,000	49	29.24	9.75	497.27	18.20	6.07	309.5	SRR5762653<br>SRR5762652
10,000	49	25.31	8.44	860.92	16.68	5.56	567.19	SRR5762651<br>SRR5762650
20,000	49	13.49	4.5	917.46	2.61	0.87	177.79	SRR5762673<br>SRR5762674<br>SRR5762671
40,000	49	16.04	5.35	2,181.98	4.33	1.44	589.77	SRR5762672<br>SRR5762670
**Total**		**321.86**	**107.31**	**4,965.93**	**244.84**	**81.61**	**2,050.74**	

[1]Qualified reads were generated by filtering the low quality reads, base-calling duplicate and adapter contamination from the raw reads. For the short insert libraries we further filtered employed the kmer-based correction.

[2]Coverage was calculated under the assumption of a genome size of 3 Gb for Milu. Sequence coverage refers to the total length of generated reads, and physical coverage refers to the total cloned DNA used for the paired reads.

Table S2: 17-mer depth distribution.

K	K-mer_Num	Peak_Depth	Genome Size	Used Bases	Used Reads	X
17	76,043,217,531	25	3,041,728,701	102,132,932,205	1,592,668,741	33.31

Table S3: Summary of the C values of Cervidae and estimated milu genome sizes.

Species	C value	Estimated genome size (Gb)
Muntiacus muntjak	3.44	
Muntiacus muntjak muntjak	3.32	
Muntiacus muntjak vaginalis	2.22	
Muntiacus reevesi	2.85	
Rangifer tarandus	3.41	
***Elaphurus davidianus* (k-mer frequency distribution analysis)**		3.04
***Elaphurus davidianus* (GenomeScope analysis)**		2.78
***Elaphurus davidianus* (GCE analysis)**		3.00

Our estimated genome sizes are within the C value interval (2.22 to 3.44) of Cervidae reported in the ANIMAL GENOME SIZE DATABASE (http://www.genomesize.com/). It indicates our estimations are highly credible.

Table S4: Summary of BUSCO analysis of matches to the 4,104 mammalian BUSCOs.

	Count	Ratio
**Complete BUSCOs**	3,820	93.00%
**Complete and single-copy BUSCOs**	3,773	91.90%
**Complete and duplicated BUSCOs**	47	1.10%
**Fragmented BUSCOs**	162	3.90%
**Missing BUSCOs**	122	3.10%

Table S5: Summary of breakpoints between milu and goat genomes.

Type of breakpoints	Numbers
**Inter-chromosomal**	29,492
**Inversion**	3,412
**Translocation**	9,127
**Insertion**	38,635
**Deletion**	85,865
**Total**	166,531

Table S6: TE contents in the assembled milu genome.

	RepBase Tes		TE Proteins		De novo		Combined Tes	
	Length (bp)	%in Genome	Length (bp)	% in Genome	Length (bp)	% in Genome	Length (bp)	% in Genome
**DNA**	61,617,345	2.44	6,547,616	0.26	32,042,812	1.27	63,758,382	2.53
**LINE**	598,971,239	23.72	388,561,935	15.39	519,186,385	20.56	682,952,607	27.05
**LTR**	122,169,833	4.84	11,809,923	0.47	75,556,696	2.99	131,057,305	5.19
**SINE**	225,961,185	8.95	0	0	143,506,487	5.68	240,276,191	9.52
**Other**	938	0.000037	0	0	0	0	938	0.000037
**Unknown**	775,383	0.03	0	0	108,743,697	4.31	109,463,272	4.34
**Total**	988,095,609	39.14	406,859,243	16.11	856,669,162	33.93	1,036,273,835	41.04

Table S7: General statistics of predicted protein-coding genes.

Gene Set		Number	Average Transcript Length (bp)	Average CDS Length (bp)	Average Exon Number per Gene	Average Exon Length (bp)	Average Intron Length (bp)
**De novo**	AUGUSTUS	29,709	19,590	1,117	6.04	185	3,665
	GENSCAN	27,196	40,343	1,586	9.47	167	4,576
**Homolog**	*H.sapiens*	27,005	20,048	1,203	6.92	174	3,182
	B.taurus	25,252	20,206	1,275	7.36	173	2,977
	S.scrofa	25,856	16,893	1,138	6.47	176	2,879
**GLEAN**	--	20,324	50,392	1,570	9.86	159	5,509
**Final**	--	20,125	46,124	1,512	9.76	155	5,088

Table S8: Summary of the predicted pseudogenes.

Types	Numbers	Percent (%)
**Frame-shifted genes**	1,358	48.45
**Prematurely terminated genes**	2,801	99.93
**Frame-shifted and prematurely terminated genes**	1,357	48.41
**Total**	2,803	100.00

Table S10: Summary statistics of gene function annotation.

	Numbers of annotated genes	Percent (%) to all genes (20,125)
**InterPro**	16,954	84.24
**GO**	13,767	68.40
**KEGG**	13,961	69.37
**Swissprot**	17,745	88.17
**TrEMBL**	17,943	89.16
**Number of annotated genes**	17,973	89.31
**Number of unannotated genes**	2,152	10.69

Table S11: Distribution of single nucleotide variant (SNV) in the milu genome.

Type (alphabetical order)	Count	Percent
**Downstream gene variant**	17,476	3.59%
**Intergenic region**	344,975	70.94%
**Intron variant**	101,399	20.85%
**Missense variant**	2,309	0.48%
**Missense variant and splice region variant**	37	0.01%
**Splice acceptor variant and intron variant**	12	0.00%
**Splice donor variant and intron variant**	35	0.01%
**Splice region variant and intron variant**	213	0.04%
**Splice region variant and stop retained variant**	1	0%
**Splice region variant and synonymous variant**	32	0.01%
**Start lost**	8	0.00%
**Stop gained**	38	0.01%
**Stop lost and splice region variant**	13	0.00%
**Synonymous variant**	1,851	0.38%
**Upstream gene variant**	17,883	3.68%
**Total**	486,282	100.00%

Table S12: Summary of short ncRNA annotation.

Type		Copy(w)	Average length(bp)	Total length(bp)	% of genome
**miRNA**		1,335	98.29	131,213	0.005197
**tRNA**		893	74.04	66,118	0.002619
**rRNA**	rRNA	280	128.63	36,016	0.001426
	18S	12	228.67	2,744	0.000109
	28S	101	178.94	18,073	0.000716
	5.8S	1	118.00	118	0.000005
	5S	166	90.85	15,081	0.000597
**snRNA**	snRNA	1,441	121.19	174,631	0.006917
	CD-box	114	137.25	15,647	0.000620
	HACA-box	284	137.21	38,968	0.001543
	splicing	1,007	114.23	115,028	0.004556
**Total**		3,949	105.54	407,978	0.016159
