# Supplementary material for: Draft genome of the milu (Elaphurus davidianus)
Source: Gigascience. 2017 Dec 18;7(2):gix130. doi: 10.1093/gigascience/gix130 (PMC5824821; doi:10.1093/gigascience/gix130)
Supplement: Response_to_Reviewer_Comments_Original_Submission.pdf [file gix130_response_to_reviewer_comments_original_submission.pdf]

Author's Response To Reviewer Comments

Editor's comments:
In particular, the reviewers raise some questions regarding the quality of the assembly and the annotation, and whether the validation steps follow best practice (for example, with regard to using the same short-read data in the assembly process as a validation dataset). Please carefully consider all points of the reviewers and revise your manuscript (and your analyses, e.g. annotations, where necessary) to address these concerns. We agree that the dataset will be useful for the community, but the methods, including potential shortcomings and limitations, need to be clearly explained and discussed in the manuscript.
Reply: We appreciate the editor's positive comments on the value of the genomic resources of milu in this work. In our revised manuscript, all the reviewers' suggestions have been fully considered. Firstly, we re-estimated the milu genome size using several more models, as described below. Secondly, we re-annotated the genes using the most recent Ensembl releases (version 89) for human, cattle and pig proteins. We have also listed pseudogenes, as requested by Reviewer 2. Thirdly, we re-annotated the gene functions and gene families, and re-constructed phylogenetic relationships, using the new gene annotations. Finally, we added discussion of potential shortcomings and limitations of our results in the revised manuscript.

I note that you indicate several corresponding authors. OUP has a policy of only taking one - most responsive - author as corresponding author. The definition of corresponding authorship is one of responsiveness rather than seniority. The corresponding author is the one individual who takes primary responsibility for communication with the journal during the manuscript submission, peer review, and publication process. Please refer to the information below on our homepage and please decide based on these guidelines who should be corresponding author.
You indicate several "co-first" authors with equal contributions. Please explain in more detail in how far the contributions of all three first authors are exactly equal. See also CASRAI guidelines to define authorship roles:
Reply: We have carefully read the EDITORIAL POLICIES & REPORTING STANDARDS in the author guidelines provided on GigaScience's website. Our authorship was based on the criteria listed in the guidelines and all the authors meet the requirements. Following the policy of GigaScience that there could be at most two correspondent authors, with the approval of all the authors, now we have only listed two correspondent authors. Nowadays most genome projects are done by collaboration of several groups, both correspondent authors contributed to the design of the project and organization of the manuscript. Now Dr, Qiang Qiu is the primary correspondent author to communicate with the journal during the manuscript submission, peer review and publication process. We have modified the description of each author's contributions, as follows, and showed that the first three were approximately equal.
"Q.Q. W.W. and G.Z. conceived the study. C.Z. and L.C. designed the scientific objectives. LGC and OAR evaluated and provided samples from San Diego Zoo Global. Y.Z. collected the samples, extracted the genomic DNA and constructed the DNA libraries. C.Z. and Y.Z. estimated the milu genome size and constructed the genome assembly. C.Z., L.C. and Y.Z. carried out the quality assessment, repeat annotation and gene annotation. C.Z. and K.W. were responsible for finding species-specific genes and phylogenetic relationship construction. L.C. and C.Z. uploaded the raw reads data, genome assembly and annotation to NCBI and GigaScience GigaDB databases. C.Z., Q.Q. and W.W. wrote the manuscript. Q.Q., W.W. and

G.Z. supervised all aspects of the work to ensure the accuracy and integrity of the research and data. OAR contributed to editing the final manuscript. All authors read and approved the final manuscript."

Reviewer reports:
Reviewer #1: Summary: In this manuscript, Zhang et al. present a new genome assembly for the Milu deer (Elaphurus davidianus) consisting entirely of short read Illumina data. They generated the reference assembly using sequence data libraries of different insert sizes input into the SOAPdenovo assembly pipeline. The reported assembly quality metrics appear to be good for an assembly of this type; however, there are some details missing from the authors' report that need to be clarified.
Reply: We are grateful for the reviewer's helpful suggestion, and have tried to address all the major and minor points raised.

Major points:
1. Line 44: For non-speakers, can you provide a translation of "Sibuxiang?" The context of the sentence implies that this name has some significance related to the animal's phenotype.
Reply: "Sibuxiang" is a colloquial name for milu that has been widely used in China, and could be translated as "the four unlikes", because it has the hooves of a cow but is not a cow, the head of a horse but is not a horse, antlers of a deer but is not a deer, and the tail of a donkey but is not a donkey. In ancient times, most Chinese people regarded the milu as a mythical creature due to its rarity and strange appearance. We have briefly provided this information in the revised manuscript, as follows:
"…Milu also has a colloquial name in China, Sibuxiang, which could be translated as "the four unlikes", because it has the hooves of a cow but is not a cow, the head of a horse but is not a horse, antlers of a deer but is not a deer, and the tail of a donkey but is not a donkey..." (p4 lines 44-47).

2. Line 64: Were proper animal care and use protocols followed in the extraction of the sample from the reference animal?
Reply: We thank the reviewer for pointing out this. We apologized for the omitting of the details on this part. We contacted with our collaborators of San Diego Zoo again and we verified the animal care and use protocols were performed in accordance with the San Diego Zoo Global's Institutional Animal Care and Use Committee policies and the ethical guidelines of Northwestern Polytechnical University and BGI-Shenzhen Laboratory Animal Care and Use Committee. We added an ethics statement and the authorship of our San Diego Zoo collaborators in the revised manuscript (p1 lines 3-4 and 10; p13 lines 252-259).

3. Line 69: Why were the short-insert library read pairs only sequenced to 49 bp in length? This seems to be very counterintuitive given that these libraries were eventually used to close gaps in SOAPdenovo scaffolds.
Reply: We thank the reviewer for spotting this mistake: the read lengths for the short-insert and long-insert libraries were 100 bp and 49 bp, respectively. We have corrected this error (p5 line73).

4. Line 84: The provided equation terms (e.g. "k-mer_number") do not match the definitions in

the following sentence (e.g. "N"). Please reformat the equation to display consistent terms.
Reply: We thank the reviewer for pointing out this. The “N” and “K_depth” refer to “k-mer_number” and “k-mer_depth”, respectively, in the manuscript, which we have reworded accordingly (p6 lines 88-89).

5. Line 84: How does this genome size estimate compare against estimates made with more sophisticated models? For example, the Vurture et al. 2017, GenomeScope package uses a far more sophisticated, kmer-based, estimation model.
Reply: We appreciate this important comment and agree that more sophisticated models could potentially provide more accurate indications of genome size. However, the estimate obtained with GenomeScope was 2.0 Gb, which is not reasonable. We also utilized the GCE software package, and obtained a genome size of 3.00 GB for milu, which is very close to our previous estimation. Moreover, our estimated genome size is within the C value interval (2.22 to 3.44) of Cervidae reported in the ANIMAL GENOME SIZE DATABASE (http://www.genomesize.com/). Thus, the GenomeScope model did not work in this case, for unknown reasons. We have added a new Table displaying this finding in the revised manuscript (Table S2; p6 lines 89-94).

6. Line 93: I don't understand why or how scaffolds generated with GapCloser were chosen to be split into new contigs. I believe - from the context of this paragraph - that GapCloser was used to fill SOAPdenovo scaffold gaps, but that unfilled gaps were broken into separate contigs. The split contigs and scaffolds were then subjected to another round of scaffolding using SSPACE. Is this correct? If so, the paragraph needs to be reworded to best reflect the authors' exact protocols.
Reply: The reviewer’s understanding is correct and we apologize for the unclear description in this paragraph, which we have amended as follows:
“Then we used GapCloser (version 1.12) [10] to fill the gaps of initial scaffolds using short-insert sizes PE reads (170, 500 and 800 bp). The initial scaffolds were then divided into scaff-tigs by the unfilled gaps. The divided scaff-tigs were connected to final scaffolds using SSPACE (version 3.0) [14] with the following parameters: -x 0, -z 200, -g 2, -k 2, -n 10. These final scaffolds’ gaps were also closed by GapCloser” (p6 lines 99-104).

7. Line 102: Was this validation dataset separate from the short reads used to construct the reference assembly itself, or were these reads used to generate the reference assembly? Using sequence data from libraries that were not included in the preparation of the reference assembly would give an unbiased estimate of assembly quality.
Reply: The reviewer raises a good point, and we agree that using sequence data from libraries that were not included in preparation of the reference assembly could give an unbiased estimation. Since all the previous data had been used in the reference assembly and there was also no more tissue from the de novo sequencing individual, we therefore re-sequenced another male milu deer and mapped the clean reads on this genome assembly using SOAPaligner with the same parameters. The results demonstrate that $<2\%$ of bases had a sequencing depth less than 10. This alignment also confirmed the accuracy at base level of this reference assembly. We have re-written the relevant sentence in the revised manuscript to make it clear for reader. (Fig. S3 and p7 lines 112-116).

8. Line 111: Which version of the goat genome was used here? The reference genome for this

species has changed recently, so a specific accession ID is needed. Please provide literature references for each reference assembly used, if possible.
Reply: We thank the reviewer for this important point. We used the recently published version of the goat genome, ARS1. We have added the accession ID for this goat genome assembly and cited relevant literature for each reference assembly in our revised manuscript (p7 lines 122-124).

9. Line 112: What was the percent identity cutoff for MUMs in the NUCmer alignments used to determine synteny? A matching synteny block with less than 50% ID is meaningless in terms of sequence-to-sequence comparisons even at a large scale.
Reply: Yes, as mentioned in the revised manuscript, the percent identity cutoff for MUMs was 50% (p7 lines 126-127).

10. Line 117: How were structural variants assessed? By strict alignment metrics between the two assemblies? Were gaps considered when making structural variant calls? More details are needed here. The inclusion of structural variant size metrics (ie. average size and standard deviations) in Table S2 is necessary to assess the structural fidelity of this assembly against the goat reference.
Reply: We thank the reviewer for raising this point. The aim of this comparison was to estimate the quality of our genome assembly rather than find the variation events. As it was not easy for us to classify each structural variation (SV) event between a chromosome-level and a scaffold-level genome sequences from synteny alignment, we had chosen to scan the edge of every SV event (breakpoint) instead of a complete SV event. We then summarized the densities of these breakpoints, which were comparable to findings in another study (Kun Wang, 2016). To clarify this, we have reworded the description in the revised manuscript (p7-8 lines 128-133).

Reviewer #2: This data note describes the generation of the draft genome of the Milu. This is an interesting species of deer that has a unique phenotype and experienced a bottleneck as they were almost extinct, but the population has started to recover and has been re-introduced to China. This paper does deliver what it sets out to describe.
Reply: We appreciate the reviewer's positive comments on this manuscript and we appreciate your suggestions, which have been highly useful for improving our manuscript.

The main issues I have are with the following section:
Gene annotation:
1. The main problem I have is that for the homology based predictions the Ensembl gene annotation used is very old. Human e60 is over 7 years old and doesn't contain the finished first pass manual annotation. I would expect the predicted number of genes to be falsely high. The analyses should be re-run for the latest version of Ensembl.
Reply: We thank the reviewer for raising this important issue. We re-ran the homology predictions using the latest Ensembl version (release 89), as suggested. The homology predictions results were integrated with our de novo predictions, and a total of 20,125 genes were obtained. We also applied BUSCO to estimate the annotation quality of the new version, which indicated 93.7% of completeness. The tables, figures and evolutionary analysis have been updated accordingly.

2. There was also no mention of pseudogenes. I am assuming that the "frame-shifted and prematurely terminated genes" that were removed from the gene set could well be pseudogenes. It would have been nice to have an idea of numbers of these, as these could be interesting data?
Reply: The reviewer makes a good point. In our revised manuscript, we regard the frame-shifted and prematurely terminated genes as pseudogenes. Candidate pseudogenes were printed out together with candidate true genes when we ran GeneWise alignment, and were filtered out if they were overlapped with the regions of the final true gene set. We identified a total of 2,803 pseudogenes (1,358 frame-shifted and 2,801 prematurely terminated genes). We have added a new table showing these results in the revised manuscript (Table S7 and S8; p9 lines 166-168).

3. Putative noncoding RNAs are discussed, but these are in fact short ncRNAs which need to be added as a definition as noncoding RNA is too big a category and doesn't describe the work that was undertaken here. lncRNAs were not addressed at all, despite their rarely being conserved between species and the homology-based approach would not have worked, some mention should have been made of these to show that the full repertoire of RNAs has been considered.
Reply: We agree with the referee that lncRNAs are rarely conserved between species, and a homology-based approach rarely detects them. The putative noncoding RNAs discussed in this paper were short ncRNAs, as explicitly stated now in the revised manuscript (p10 lines 180 and 184).

4. Nomenclature has not been mentioned at all. Perhaps some known genes could have been used to add context to table S6? Has the naming convention for human been followed? I'd like to know more about genes and gene families in general that have been found. Figure 2a needs more defining. What are the gene families that are specific to Milu? I'd like to know.
Reply: We thank the reviewer for raising this important issue. The nomenclature followed the naming convention for humans and we have added the name of each gene in a gff file. We reconstructed phylogenetic relationships using the new gene set and re-analyzed species-specific genes. We found 69 milu species-specific gene families corresponding to 167 milu species-specific genes, which were enriched in 4 GO categories related to hormone activity, structural constituent of ribosome and etc. What’s more, we also added the analysis of the milu expanded gene families, which were enriched in 34 GO categories, and their functions were mainly associated with motor activity, ATPase activity, calcium ion binding and etc. Three new tables were added to show these results (Table S12-S14; p10-11 lines 192-198; p18 lines 396-399).

Minor points:
Library construction:
5. ……..male milu tissue…….: Which tissue?
Reply: We have added the following details to the revised manuscript to clarify this: “Genomic DNA was extracted from a male milu bred at the San Diego Zoo Safari Park, Escondido, California, USA utilizing heart tissue collected at necropsy, (NCBI Taxonomy ID, 43332)” (p5 lines 67-68).

6. There are several typos and sections that would benefit from some re-writing (detailed below).
Reply: We have rewritten these sentences.

Background:

7. …….due to intense human an natural pressures………….: such as? what does this mean?
Reply: We have rewritten this sentence (p4 lines47-48 "Due to intense human and natural pressures, such as excessive hunting by humans and habitat degradation, milu became extinct.")

8. This sentence needs re-writing as its too long and a bit muddled.
Reply: Done.

9. …..Special phenotype……: I think specific phenotype would be better.
Reply: The "Special phenotype" here mainly referred to its unusual appearance. We have modified the text as follows:
"…little is still known about the genetic architecture underlying its unique phenotypic features, such us its unusual appearance, and the population dynamics during the milu's recovery from the severe bottleneck..." (p4 lines 59-61)

10. Table S6: This doesn't make sense. What is it showing me?
Reply: We apologize for omitting the total number and the percentage of annotated genes in Table S6. We have added total values to this table. We also re-ran the gene function annotation using the new gene set, and revised the statistics presented in the table accordingly (Table S9).

11. Table S8: Need to add total for number of miRNAs.
Reply: Changed as suggested (Table S11).
