# Supplementary material for: Draft genome of the milu (Elaphurus davidianus)
Source: Gigascience. 2017 Dec 18;7(2):gix130. doi: 10.1093/gigascience/gix130 (PMC5824821; doi:10.1093/gigascience/gix130)
Supplement: Response_to_Reviewer_Comments_Revision_1.pdf [file gix130_response_to_reviewer_comments_revision_1.pdf]

Author's Response To Reviewer Comments

Editor's comments:

1) I agree with Reviewer 1 that the sequence data of the unrelated milu should also be made publicly available.
Reply: Thanks for pointing out that. We have released the short reads obtained from sequencing the unrelated milu deer in the NCBI database, and added the SRA accession numbers in the revised manuscript (p. 10, line 214).

2) Our data curators will contact you to prepare the supporting dataset that will be posted in our repository, GigaDB.
Reply: We have received the curator's mail and provided all the required information.

3) Please provide all relevant SRA (Short Reads Archive) accession numbers in the "data availability" section of your manuscript
Reply: The SRA ID of both individuals have been added in the manuscript (See Table S1 and line 213).

I noticed that you submitted data to the Genome Sequence Archive at the BIG data center. Please note that this database is not part of the community-accepted INSDC collaboration (http://www.insdc.org/). All sequence data reported in the manuscript must be made available via INSDC databases. Referencing the BIG data center (citations #39/#40) is not necessary, please remove these from the reference list.
Reply: The references to the BIG data center have been removed from the reference list and the relevant description in the supporting data paragraph are also deleted.

4) Please also address all other additional comments of both reviewers in a revised manuscript (e.g. regarding the FRC plots).
Reply: In our revised manuscript, all the reviewers' suggestions have been fully considered and addressed.

5) Please carefully check your revised manuscript for grammatical mistakes and unclear wording (ideally with the help of a native English speaker).
Reply: As requested, the revised manuscript has been edited by a professional, native English-speaking editor with a PhD in a relevant discipline, who has addressed both grammatical errors and unclear phrasing.

Reviewer reports:
Reviewer #1: Summary: In this revision, the authors have addressed most of my previous points. There are still minor points within the manuscript that need additional clarity, and I would highly recommend that the authors deposit any additional data they generated in public repositories. After these corrections, I think that the published data will be useful to the Artiodactyla research community.

Minor points:

1. Line 112: I would request that the authors deposit the sequence data from this unrelated Milu deer in a public repository so that other groups can reproduce their results.
Reply: We have released the short reads obtained from sequencing the other milu deer in the NCBI database (SRA ID: SRR6287186) and added the SRA ID in the manuscript (p10 line 211).

2. Line 120: The FRC plot shows a clear distinction between the Milu deer assembly and other published references. What is the reason for this discrepancy? The FRC_align software used to generate the input data for this plot requires alignments of short-insert and, optionally, long-insert libraries against each reference. What short read datasets were used for each reference genome in the calculation?
Reply: We thank the reviewer for pointing this out. We expected to obtain a better FRCurve for the milu deer assembly because the reads used for the analysis were generated from the milu sequencing. FRC_align analysis was applied to assess the error in our genome assembly, and it did not indicate that the milu deer assembly is absolutely more accurate than genome assemblies for cattle, goat and sheep.
Short-insert and long-insert reads providing ca. 27- and 14-fold coverage, respectively, were randomly selected from the milu sequencing data, and aligned to the milu, cattle, goat and sheep assemblies to generate bam files for FRC_align analysis.

3. Line 89: It would be useful to include the GenomeScope estimate of genome size here and briefly mention why it is not an accurate measure of genome size in this species.
Reply: We agree. Previously, we obtained the GenomeScope estimate strictly following instructions on the website, with recommended parameters and the kmer length ranging from 17 to 21. The first two steps were carried out by Jellyfish with parameters (jellyfish count -C -m 17 –t 20; jellyfish histo –t 20). The value of the parameter, "–m", was the same as the kmer length of the GenomeScope online run. These two steps were applied to obtain the k-mer spectrum. In the final step, running GenomeScope online, the modeling failed to converge with the kmer length set at 19 to 21. We only obtained convergence with the kmer length set at 17, but the estimated genome sizes were much lower than normal. (See Fig. R1; "len" represents the estimated genome size with kmer length set at 17 and default Max kmer coverage).

We carefully re-read the GenomeScope online guidance and FAQ pages. In one presented case the estimated genome size tended be higher with the parameter Max kmer coverage (default value: 10,000) set at 100,000 or 1,000,000. Thus, we tried setting Max kmer coverage at values ranging from 50,000 to 1,000,000 with the kmer length set at 17. Unfortunately, the genome size estimates were only slightly improved and still far from normal (see Fig. R2, presenting results for an analysis with kmer length set at 17 and Max kmer coverage at 100,000).

To our surprise, there were marked improvements when we set the Max kmer coverage at 200,000 and kmer length at 21 unintentionally. The modeling converged successfully in the final step and the estimated genome size was 2.606 Gb; much closer to estimates obtained by the other approaches than previous GenomeScope estimates (See Fig. R3). Then we realized that both the kmer length and Max kmer coverage were key factors for GenomeScope estimates. Subsequently, we tried a higher kmer length (21-39) with Max kmer coverage ranging from 50,000 to 500,000. The estimated genome sizes we obtained were 2.575 to 2.784 Gb (See Fig.

R4, showing results obtained with the kmer length set at 39 and Max kmer coverage at 100,000). Despite up to 210 Mbp discrepancies among the new GenomeScope estimates, and at least 216 Mbp discrepancy between them and GCE estimates, they were more acceptable than previous estimates.

We consulted the GenomeScope publishers regarding this issue, and obtained the following explanation from Michael Schatz, director of the GenomeScope project.
"…The model fits look pretty good for the higher kmer lengths (k=21 and above) in that the main peaks fit the model closely and lead to an estimated genome size of about 2.5 to 2.7Gbp. For k=17, it looks like the modeling got very confused about which peak represented the homozygous kmers so I would not trust that result at all. Other than k=17, the wide range in genome size this is largely because of the max kmer coverage value - we find that often very high frequency kmers are not part of the genome, but instead are some sort of contaminating sequence: phiX spikein, high coverage mitochondria sequences, microbial contaminates, etc. Even though these genomes are typically small, they can contribute to the overall genome size estimate if they occur in hundreds of thousands to millions of copies. So the goal of the max kmer coverage threshold is to exclude these very high coverage kmers from consideration. This is usually pretty effective, but can underestimate the overall genome size if there are real high coverage kmers from the genome, such as from the centromeres or telomeres … And I think it would be fair to estimate the genome size at around 2.7Gb as this is supported by multiple methods."

Accordingly, we regard the estimate obtained with Kmer set at 39 as the best estimate yielded by this approach. We have added this estimate and the corresponding parameters in our revised manuscript (Lines 85-87, Fig. S2)

4. There are several grammar errors within the manuscript which need to be corrected. I believe the manuscript would benefit from light type-editing.
Reply: We thank the reviewer for pointing out this. The paper has been edited by a professional, native English-speaking editor who has a PhD in a relevant discipline.

(Please see Fig. R1-R4 in the docx file, "Milu-response-R2 letter".)

Reviewer #2: The authors have taken on board the comments that I made in my review and added additional information.
1. I think there is still a little confusion regarding gene nomenclature. By this I am referring to the actual symbol and description, for example:BRCA2:BRCA2, DNA repair associated. Has an actual gene set been produced and given symbols?
For point 4 from the reviewers comments the authors have revised and added to the gene ontology, which is useful, but not what I was referring to.
Reply: We thank the reviewer for highlighting this issue. For the nomenclature, we aligned the milu deer genes against coding genes of cattle. Then we regarded the gene symbol of the best match (coding gene of cattle) as the gene symbol for each aligned Milu deer gene. These gene symbols and related descriptions have been added to the gff file of this assembly (See Fig. R5, a screenshot of the mRNA lines in gff file stored in GigaDB).

Regarding point 4 of the last round of reviewer's comments, the previous version of the paper reported analyses of milu species-specific gene families and GO analysis conducted to examine their possible functions. In the revised version we have also provided a gene list of milu's specific gene families (See Table S13).

2. Related to this, point 10, a new table has been generated (S9) which shows the percentage coverage from different resources that the authors found in their gene set. This is a higher level analysis than I was referring to, but is valid and in the context of this paper seems useful.
The new table S11 is exactly what Is was referring to and is much more helpful.
Reply: We appreciate the reviewer's positive comments on our added work.

3. There is a typo on line 395: Veen (should be Venn).
Reply: Corrected.

(Please see Fig. R5 in the docx file, "Milu-response-R2 letter".)
