# Supplementary material for: Draft genome of the milu (Elaphurus davidianus)
Source: Gigascience. 2017 Dec 18;7(2):gix130. doi: 10.1093/gigascience/gix130 (PMC5824821; doi:10.1093/gigascience/gix130)
Supplement: Reviewer_1_Report_(Original_Submission) -- Derek Bickhart — 11 Jul 2017 Reviewed [file gix130_reviewer_1_report_(original_submission).pdf]

Reviewer Report

Title: Draft genome of the milu (Elaphurus davidianus)

Version: Original Submission **Date:** 7/11/2017

Reviewer name: Derek Bickhart

Reviewer Comments to Author:

Summary: In this manuscript, Zhang et al. present a new genome assembly for the Milu deer (Elaphurus davidianus) consisting entirely of short read Illumina data. They generated the reference assembly using sequence data libraries of different insert sizes input into the SOAPdenovo assembly pipeline. The reported assembly quality metrics appear to be good for an assembly of this type; however, there are some details missing from the authors' report that need to be clarified.Major points:Line 44: For non-speakers, can you provide a translation of "Sibuxiang?" The context of the sentence implies that this name has some significance related to the animal's phenotype.Line 64: Were proper animal care and use protocols followed in the extraction of the sample from the reference animal?Line 69: Why were the short-insert library read pairs only sequenced to 49 bp in length? This seems to be very counterintuitive given that these libraries were eventually used to close gaps in SOAPdenovo scaffolds.Line 84: The provided equation terms (e.g. "k-mer_number") do not match the definitions in the following sentence (e.g. "N"). Please reformat the equation to display consistent terms.Line 84: How does this genome size estimate compare against estimates made with more sophisticated models? For example, the Vurture et al. 2017, GenomeScope package uses a far more sophisticated, kmer-based, estimation model.Line 93: I don't understand why or how scaffolds generated with GapCloser were chosen to be split into new contigs. I believe - from the context of this paragraph - that GapCloser was used to fill SOAPdenovo scaffold gaps, but that unfilled gaps were broken into separate contigs. The split contigs and scaffolds were then subjected to another round of scaffolding using SSPACE. Is this correct? If so, the paragraph needs to be reworded to best reflect the authors' exact protocols.Line 102: Was this validation dataset separate from the short reads used to construct the reference assembly itself, or were these reads used to generate the reference assembly? Using sequence data from libraries that were not included in the preparation of the reference assembly would give an unbiased estimate of assembly quality.Line 111: Which version of the goat genome was used here? The reference genome for this species has changed recently, so a specific accession ID is needed. Please provide literature references for each reference assembly used, if possible.Line 112: What was the percent identity cutoff for MUMs in the NUCmer alignments used to determine synteny? A matching synteny block with less than 50% ID is meaningless in terms of sequence-to-sequence comparisons even at a large scale.Line 117: How were structural variants assessed? By strict alignment metrics between the two assemblies? Were gaps considered when making structural variant calls? More details are needed here. The inclusion of structural variant size metrics (ie. average size and standard deviations) in Table S2 is necessary to assess the structural fidelity of this assembly against the goat reference.

Level of Interest

Please indicate how interesting you found the manuscript: An article of limited interest

Quality of Written English

Please indicate the quality of language in the manuscript: Needs some language corrections before being published

Declaration of Competing Interests

Please complete a declaration of competing interests, considering the following questions:

- Have you in the past five years received reimbursements, fees, funding, or salary from an organisation that may in any way gain or lose financially from the publication of this manuscript, either now or in the future?
- Do you hold any stocks or shares in an organisation that may in any way gain or lose financially from the publication of this manuscript, either now or in the future?
- Do you hold or are you currently applying for any patents relating to the content of the manuscript?
- Have you received reimbursements, fees, funding, or salary from an organization that holds or has applied for patents relating to the content of the manuscript?
- Do you have any other financial competing interests?
- Do you have any non-financial competing interests in relation to this paper?

If you can answer no to all of the above, write 'I declare that I have no competing interests' below. If your reply is yes to any, please give details below.

I declare that I have no competing interests

I agree to the open peer review policy of the journal. I understand that my name will be included on my report to the authors and, if the manuscript is accepted for publication, my named report including any attachments I upload will be posted on the website along with the authors' responses. I agree for my report to be made available under an Open Access Creative Commons CC-BY license (http://creativecommons.org/licenses/by/4.0/). I understand that any comments which I do not wish to be included in my named report can be included as confidential comments to the editors, which will not be published.

I agree to the open peer review policy of the journal
