# Supplementary material for: Draft genome of the milu (Elaphurus davidianus)
Source: Gigascience. 2017 Dec 18;7(2):gix130. doi: 10.1093/gigascience/gix130 (PMC5824821; doi:10.1093/gigascience/gix130)
Supplement: Reviewer_1_Report_(Revision_1) -- Derek Bickhart — 24 Oct 2017 Reviewed [file gix130_reviewer_1_report_(revision_1).pdf]

Reviewer Report

Title: Draft genome of the milu (Elaphurus davidianus)

Version: Revision 1 **Date:** 10/24/2017

Reviewer name: Derek Bickhart

Reviewer Comments to Author:

Summary: In this revision, the authors have addressed most of my previous points. There are still minor points within the manuscript that need additional clarity, and I would highly recommend that the authors deposit any additional data they generated in public repositories. After these corrections, I think that the published data will be useful to the Artiodactyla research community.Minor points:Line 112: I would request that the authors deposit the sequence data from this unrelated Milu deer in a public repository so that other groups can reproduce their results.Line 120: The FRC plot shows a clear distinction between the Milu deer assembly and other published references. What is the reason for this discrepancy? The FRC_align software used to generate the input data for this plot requires alignments of short-insert and, optionally, long-insert libraries against each reference. What short read datasets were used for each reference genome in the calculation?Line 89: It would be useful to include the GenomeScope estimate of genome size here and briefly mention why it is not an accurate measure of genome size in this species.There are several grammar errors within the manuscript which need to be corrected. I believe the manuscript would benefit from light type-editing.

Level of Interest

Please indicate how interesting you found the manuscript: An article whose findings are important to those with closely related research interests

Quality of Written English

Please indicate the quality of language in the manuscript: Needs some language corrections before being published

Declaration of Competing Interests

Please complete a declaration of competing interests, considering the following questions:

- Have you in the past five years received reimbursements, fees, funding, or salary from an organisation that may in any way gain or lose financially from the publication of this manuscript, either now or in the future?
- Do you hold any stocks or shares in an organisation that may in any way gain or lose financially from the publication of this manuscript, either now or in the future?

- Do you hold or are you currently applying for any patents relating to the content of the manuscript?
- Have you received reimbursements, fees, funding, or salary from an organization that holds or has applied for patents relating to the content of the manuscript?
- Do you have any other financial competing interests?
- Do you have any non-financial competing interests in relation to this paper?

If you can answer no to all of the above, write 'I declare that I have no competing interests' below. If your reply is yes to any, please give details below.

I declare that I have no competing interests

I agree to the open peer review policy of the journal. I understand that my name will be included on my report to the authors and, if the manuscript is accepted for publication, my named report including any attachments I upload will be posted on the website along with the authors' responses. I agree for my report to be made available under an Open Access Creative Commons CC-BY license (http://creativecommons.org/licenses/by/4.0/). I understand that any comments which I do not wish to be included in my named report can be included as confidential comments to the editors, which will not be published.

I agree to the open peer review policy of the journal
