# Supplementary material for: Draft genome of the milu (Elaphurus davidianus)
Source: Gigascience. 2017 Dec 18;7(2):gix130. doi: 10.1093/gigascience/gix130 (PMC5824821; doi:10.1093/gigascience/gix130)
Supplement: Reviewer_2_Report_(Original_Submission) -- Jane Loveland — 17 Jul 2017 Reviewed [file gix130_reviewer_2_report_(original_submission).pdf]

Reviewer Report

Title: Draft genome of the milu (Elaphurus davidianus)

Version: Original Submission **Date:** 7/17/2017

Reviewer name: Jane Loveland

Reviewer Comments to Author:

This data note describes the generation of the draft genome of the Milu. This is an interesting species of deer that has a unique phenotype and experienced a bottleneck as they were almost extinct, but the population has started to recover and has been re-introduced to China. This paper does deliver what it sets out to describe.The main issues I have are with the following section:Gene annotation:The main problem I have is that for the homology based predictions the Ensembl gene annotation used is very old. Human e60 is over 7 years old and doesn't contain the finished first pass manual annotation. I would expect the predicted number of genes to be falsely high. The analyses should be re-run for the latest version of Ensembl.There was also no mention of pseudogenes. I am assuming that the "frame-shifted and prematurely terminated genes" that were removed from the gene set could well be pseudogenes. It would have been nice to have an idea of numbers of these, as these could be interesting data?Putative noncoding RNAs are discussed, but these are in fact short ncRNAs which need to be added as a definition as noncoding RNA is too big a category and doesn't describe the work that was undertaken here. lncRNAs were not addressed at all, despite their rarely being conserved between species and the homology-based approach would not have worked, some mention should have been made of these to show that the full repertoire of RNAs has been considered.Nomenclature has not been mentioned at all. Perhaps some known genes could have been used to add context to table S6? Has the naming convention for human been followed? I'd like to know more about genes and gene families in general that have been found. Figure 2a needs more defining. What are the gene families that are specific to Milu? I'd like to know.Minor points:Library construction:……..male milu tissue…….: Which tissue?There are several typos and sections that would benefit from some re-writing (detailed below).Background:…….due to intense human an natural pressures………….: such as? what does this mean?This sentence needs re-writing as its too long and a bit muddled……Special phenotype……: I think specific phenotype would be better.Table S6: This doesn't make sense. What is it showing me?Table S8: Need to add total for number of miRNAs.

Level of Interest

Please indicate how interesting you found the manuscript: An article whose findings are important to those with closely related research interests

Quality of Written English

Please indicate the quality of language in the manuscript: Needs some language corrections before being published

Declaration of Competing Interests

Please complete a declaration of competing interests, considering the following questions:

- Have you in the past five years received reimbursements, fees, funding, or salary from an organisation that may in any way gain or lose financially from the publication of this manuscript, either now or in the future?
- Do you hold any stocks or shares in an organisation that may in any way gain or lose financially from the publication of this manuscript, either now or in the future?
- Do you hold or are you currently applying for any patents relating to the content of the manuscript?
- Have you received reimbursements, fees, funding, or salary from an organization that holds or has applied for patents relating to the content of the manuscript?
- Do you have any other financial competing interests?
- Do you have any non-financial competing interests in relation to this paper?

If you can answer no to all of the above, write 'I declare that I have no competing interests' below. If your reply is yes to any, please give details below.

I declare that I have no competing interests

I agree to the open peer review policy of the journal. I understand that my name will be included on my report to the authors and, if the manuscript is accepted for publication, my named report including any attachments I upload will be posted on the website along with the authors' responses. I agree for my report to be made available under an Open Access Creative Commons CC-BY license (http://creativecommons.org/licenses/by/4.0/). I understand that any comments which I do not wish to be included in my named report can be included as confidential comments to the editors, which will not be published.

I agree to the open peer review policy of the journal
