# Supplementary material for: Draft genome of the milu (Elaphurus davidianus)
Source: Gigascience. 2017 Dec 18;7(2):gix130. doi: 10.1093/gigascience/gix130 (PMC5824821; doi:10.1093/gigascience/gix130)
Supplement: Reviewer_2_Report_(Revision_1) -- Jane Loveland — 06 Nov 2017 Reviewed [file gix130_reviewer_2_report_(revision_1).pdf]

Reviewer Report

Title: Draft genome of the milu (Elaphurus davidianus)

Version: Revision 1 **Date:** 11/6/2017

Reviewer name: Jane Loveland

Reviewer Comments to Author:

The authors have taken on board the comments that I made in my review and added additional information.I think there is still a little confusion regarding gene nomenclature. By this I am referring to the actual symbol and description, for example:BRCA2:BRCA2, DNA repair associated. Has an actual gene set been produced and given symbols?For point 4 from the reviewers comments the authors have revised and added to the gene ontology, which is useful, but not what I was referring to.Related to this, point 10, a new table has been generated (S9) which shows the percentage coverage from different resources that the authors found in their gene set. This is a higher level analysis than I was referring to, but is valid and in the context of this paper seems useful.The new table S11 is exactly what I was referring to and is much more helpful.There is a typo on line 395: Veen (should be Venn).

Level of Interest

Please indicate how interesting you found the manuscript: An article whose findings are important to those with closely related research interests

Quality of Written English

Please indicate the quality of language in the manuscript: Acceptable

Declaration of Competing Interests

Please complete a declaration of competing interests, considering the following questions:

- Have you in the past five years received reimbursements, fees, funding, or salary from an organisation that may in any way gain or lose financially from the publication of this manuscript, either now or in the future?
- Do you hold any stocks or shares in an organisation that may in any way gain or lose financially from the publication of this manuscript, either now or in the future?
- Do you hold or are you currently applying for any patents relating to the content of the manuscript?
- Have you received reimbursements, fees, funding, or salary from an organization that holds or has applied for patents relating to the content of the manuscript?

- Do you have any other financial competing interests?
- Do you have any non-financial competing interests in relation to this paper?

If you can answer no to all of the above, write 'I declare that I have no competing interests' below. If your reply is yes to any, please give details below.

I have no competing interests

I agree to the open peer review policy of the journal. I understand that my name will be included on my report to the authors and, if the manuscript is accepted for publication, my named report including any attachments I upload will be posted on the website along with the authors' responses. I agree for my report to be made available under an Open Access Creative Commons CC-BY license (http://creativecommons.org/licenses/by/4.0/). I understand that any comments which I do not wish to be included in my named report can be included as confidential comments to the editors, which will not be published.

I agree to the open peer review policy of the journal
